# Cancer risks related to intellectual disabilities: A systematic review

**DOI:** 10.1002/cam4.7210

**Published:** 2024-04-30

**Authors:** Amina Banda, Jenneken Naaldenberg, Aura Timen, Agnies van Eeghen, Geraline Leusink, Maarten Cuypers

**Affiliations:** ^1^ Department of Primary and Community Care Radboud university medical centre Nijmegen the Netherlands; ^2^ Emma Children's Hospital Amsterdam University Medical Centers Amsterdam the Netherlands; ^3^ 'S Heeren Loo Amersfoort the Netherlands

## Abstract

**Background:**

People with intellectual disabilities (ID) face barriers in cancer care contributing to poorer oncological outcomes. Yet, understanding cancer risks in the ID population remains incomplete.

**Aim:**

To provide an overview of cancer incidence and cancer risk assessments in the entire ID population as well as within ID‐related disorders.

**Methods:**

This systematic review examined cancer risk in the entire ID population and ID‐related disorders. We systematically searched PubMed (MEDLINE) and EMBASE for literature from January 1, 2000 to July 15, 2022 using a search strategy combining terms related to cancer, incidence, and ID.

**Results:**

We found 55 articles assessing cancer risks in the ID population at large groups or in subgroups with ID‐related syndromes, indicating that overall cancer risk in the ID population is lower or comparable with that of the general population, while specific disorders (e.g., Down's syndrome) and certain genetic mutations may elevate the risk for particular cancers.

**Discussion:**

The heterogeneity within the ID population challenges precise cancer risk assessment at the population level. Nonetheless, within certain subgroups, such as individuals with specific ID‐related disorders or certain genetic mutations, a more distinct pattern of varying cancer risks compared to the general population becomes apparent.

**Conclusion:**

More awareness, and personalized approach in cancer screening within the ID population is necessary.

## 1 INTRODUCTION

Intellectual disability (ID) is characterized by significant limitations in both intellectual and adaptive functioning in conceptual, social, and adaptive skills which are developed before adulthood.^1^ People with an ID face inequalities throughout the entire spectrum of cancer care,^2^ including barriers to participating in screening and detection of cancer at more advanced stages than in the general population.^3^ Consequently, resulting in higher cancer‐related mortality rates.^4^ It is important to recognize that at least some of these malignancies and deaths could be avoided through adequate prevention, and earlier interventions.^3^, ^5^

While similar disparities have been found in the broader population of people with mental illness,^6^ individuals with ID represent a distinct subgroup that face unique challenges due to their limitations in cognitive functioning and adaptive behavior.^1^ This is particularly significant because the onset and developmental patterns of cancer can be different in people with ID as compared with the general population. Moreover, the (genetic) cause of the ID may play a role in the pathway of tumor development or provide protective effects. For example, Down's syndrome (DS) has been linked to an increased risk of childhood leukemia,^7^, ^8^ but also to a reduced risk for solid tumors.^9^, ^10^ Similarly, patients with neurofibromatosis type 1 (NF1) are recognized to have a higher risk of several types of cancerous tumors.^11^ While many of so called genetic neurodevelopmental disorders (GNDs) are characterized by the development of ID,^12^ this does not have to be case for all GNDs. For example, in NF1 and xeroderma pigmentosum only a proportion of patients develop an ID.^13^, ^14^

Nongenetic cancer risk factors, such as smoking, substance use and misuse, obesity, and overall poorer health, are more prevalent in people with ID.^15^, ^16^, ^17^, ^18^ Furthermore, age is a disruptive factor in understanding cancer risks in people with ID, as their life expectancy is generally lower than people without ID.^19^ Particularly because age‐related health problems and frailty start at a younger age in this group.^20^ In addition, with increasing age, major health issues are more common.^21^

To ensure equitable cancer care for individuals with ID, a better understanding of the specific association between ID and cancer risks is essential. Currently, systematic knowledge in this area is scarce, primarily due to the heterogeneous nature of existing literature. Various studies employed different methodologies, including in vivo studies, care reports, surveys, and population‐based studies, leading to estimates of cancer prevalence within the ID population being lower, equal, or higher than in the general population.^5^, ^22^, ^23^, ^24^ This heterogeneity presents challenges in offering tailored advice and guidelines for cancer prevention and screening, which is essential for this vulnerable group. We aim to provide an overview of cancer incidence and cancer risks assessments in the entire ID population as well as within subgroups based on specific ID‐related disorders. This knowledge will enable us to recognize the possible association between cancer risks and ID, and possibly inform strategies for cancer care among individuals with ID. Such an overview is critical for informing physicians, facilitating accurate diagnoses, treatment, and prevention.

## 2 MATERIALS AND METHODS

### 2.1 Study design

This systematic review was conducted following the PRISMA guidelines extension for systematic reviews (Appendix S1). The methodological framework of the study was preregistered in the PROSPERO International Prospective Register of Systematic Reviews (CRD42022320627).

### 2.2 Search strategy

Articles for this systematic review were identified through PubMed (National Center for Biotechnology; including MEDLINE) and EMBASE searches executed on July 15, 2022. The search strategy was established in collaboration with a medical research librarian. To provide the articles needed for the research question, to identify the possible association between ID and cancer risks assessments through cancer incidence, the search string consisted of four concepts: cancer, incidence, a broad term for ID, and a list of specific ID‐related disorders. The list of ID‐related disorders was composed using the human phenotype ontology database (HPO), HPO:0001249 (Human Phenotype Ontology [jax.org]). We have included all ID‐related disorders that are known within this database. The complete overview of the search strategy is provided in supplement B. Finally, the search was restricted to articles published in the year 2000 or later to avoid including a high number of outdated articles, given the fast developments of genetic techniques in the past two decades. All searches were conducted by the medical research librarian and one author (A.B.).

### 2.3 Selection criteria

For the title and abstract screening, the following inclusion criteria were applied:

1. reporting on (occurrence of) malignant neoplasms as outcome measure;
2. patient population with ID, specification of association with ID, or genetic mutation causing an ID‐related disorder;
3. study design that could generate information on cancer incidence, risk differences, or recommendations on cancer prevention if available; and
4. studies including comparison groups consisting of individuals without ID.

Exclusion criteria:

1. Research where intellectual disability or ID‐related disorders were either excluded, or not clearly stated.
2. Reporting on benign neoplasm as outcome measure only;
3. studies without human subjects (i.e., in vitro studies and animal studies);
4. non‐English articles; or
5. case reports and case series.

#### 2.3.1 Data analysis

Initially, the title and abstract screening were assessed independently by two authors (A.B. and M.C.) using Rayyan.^25^ Following, full‐text articles were assessed for original data on prevalence, incidence rates, and/or risk estimates of malignant neoplasms in people with ID. The screening and full‐text assessments were performed independently by two authors (A.B. and M.C.). Discrepancies in the handling of an article were discussed until a consensus was reached about its inclusion or exclusion, and any remaining discrepancies were discussed with J.N., A.E., G.L.L., and A.T. until an agreement was reached within the team.

##### Data extraction

The patient characteristics, cancer incidence, cancer risks assessments, and screening recommendations in people with ID, if any, were extracted from the included articles. The data extraction was performed by A.B., and verified by M.C. Articles were divided on research within the overall ID population and ID‐related syndromes, and specific genetic mutation‐related disorders. From each article, data were extracted on study design, country (population), age, type of cancer, cause of the ID (if available), reported cancer incidence or prevalence with statistical measure (i.e., odds ratios; if available), and any recommendations made regarding cancer screening.

### 2.4 Methodological quality assessment and risk of bias

Quality of all included articles was assessed following the critical appraisal tools of the Joanna Briggs Institute.^72^ Assessment outcomes were summarized into five main topics: adequate sample size, study population, definition of ID and associated method to identify subjects with ID accuracy of cancer diagnoses, and appropriate statistical analysis. Each topic was scored as sufficient (+), moderate (+/−), or insufficient (−).

## 3 RESULTS

### 3.1 Article characteristics

**FIGURE 1:**
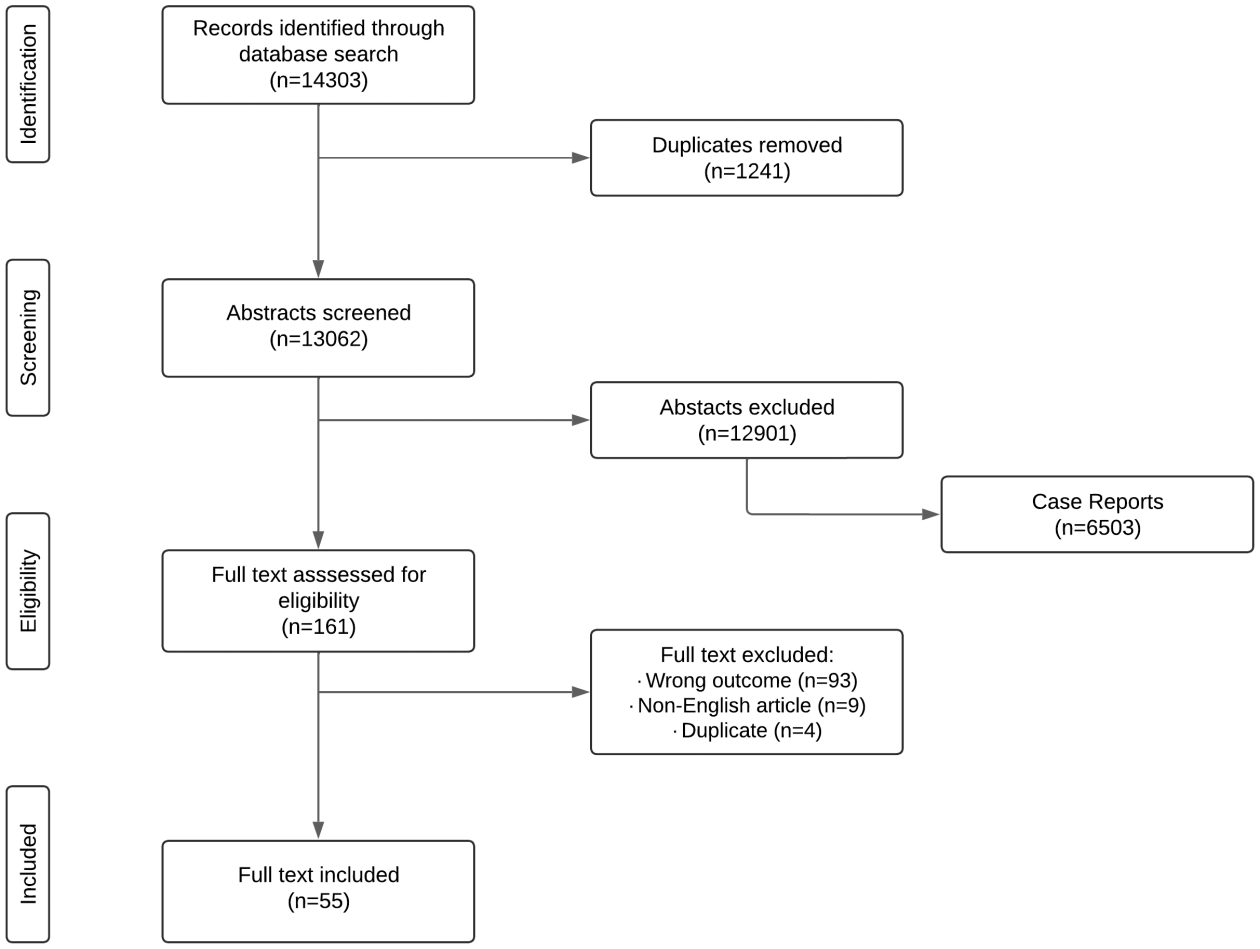
Search results and study selection flow chart.

A total of 14,303 articles (PubMed, *n* = 13,156; EMBASE, *n* = 1147) were identified in the database search, of which 55 met the inclusion criteria for full‐text screening (Figure 1). Seven papers focused on the overall ID population without specifying underlying causes of the ID (Table 1). The other 48 articles (Table 2) focused on specific ID‐related disorders, in which xeroderma pigmentosum (XP) was the most common (*n* = 12), followed by DS (*n* = 11), NF1 (*n* = 8), Fanconi anemia (FA) (*n* = 4), Fragile X syndrome (FXS) (*n* = 2), and Nijmegen breakage syndrome (NBS) (*n* = 2). One article was found for each of the following disorders: Bloom syndrome (BS), Rubinstein–Taybi syndrome, Prader–Willi syndrome, Klippel–Trenaunay syndrome, Saethre–Chotzen syndrome, Cowden syndrome, Costello syndrome, Noonan syndrome, and the sex development disorder (females carrying Y‐chromosome material; [*n* = 1]). Notably, a total of 6503 case reports were identified during the screening step. A total of 48 out of 55 papers were assessed as sufficient quality (+) while 7 were of moderate quality (+/−). No papers were found to be of insufficient quality (Supplement C).

**TABLE 1:** Overview of cancer rate in the general ID population.

Study	No. of participants	Age of the included cohorts	Study design	Time periods data collected	Population (country)	Type of cancer	Cancer incidence among people with ID	Recommendation on screening/general conclusions
Liu,Q ‘21^1^	27,956	>43 years	Population‐based cohort study	1974–2013	Sweden	All cancers	Risk for any cancer was significantly increased compared to the general population (HR = 1.57, 95% CI 1.35–1.82). Explicitly significant increase was depicted for the following cancers: esophagus, stomach, small intestine, colon, pancreas, uterus, kidney, central nervous system. The increased risk was higher among people with syndromic ID	The results suggest the need for early cancer detection and longer surveillance among individuals with ID
Patja,K ‘01^2^	2713	7–69 years	Cross‐sectional, multidisciplinary study	1967–1997	Finland	All cancers	Observed versus expected cancer incidence ratio 173/188, (SIR = 0.9; 95% CI 0.80–1.00). Neoplasms in gallbladder and thyroid gland were significantly increased. Moreover, neoplasms of the prostate, urinary tract and lung were significantly decreased	In this study the cancer incidence among people with ID was comparable to the general population
Satgé, D ‘20^3^	7936	≥55 years	Retrospective, population register‐based design	2002–2012	Sweden	All cancers	Observed versus expected cancer incidence ratio 444/877; (OR = 0.63, 95%CI 0.57–0.70)	These findings report that there is more than one third of a decreased frequency of cancer in individuals with ID compared to the general population. Nonetheless, this study confirm that cancer is a major concern in people with ID as they age. People with ID should be included in cancer prevention and screening programs
Sullivan,S ‘04^4^	9409	0–85+ years	Data‐linkage study/Cross sectional research	1982–2001	Australia	All cancers	Overall cancer incidence rate was lower than in the general population (Males: obs: 103, exp: 349.2 SIR = 0.29, 95% CI 0.24–0.36 and Females: obs: 97, exp: 215.8 SIR = 0.48, 95% CI 0.36–0.55), but incidence was significantly higher at young ages (0–4 years of age; SIR = 7.23 95% CI 4.04–11.92). Incidence rate for leukemia was increased for both sexes (males: SIR = 3.31 95% CI 1.81–5.56 and females SIR = 4.64 95% CI 2.40–8.11), mostly concerning people with DS	Overall, there were fewer cases of cancers observed in individuals with ID than expected. However the study emphasizes that cancer incidence ID rate is likely to rise with a continuing increase of life expectancy of the ID population
Cuypers,M'20^5^	65,183	0‐ ≥ 90 years	Population‐based cohort study	2012–2015	Netherlands	All cancers	Incidence rate for cancer‐care was lower for all cancers among people with ID when compared to the general population (Male: incidence rate ratio (IRR) = 0.59 95% CI 0.57–0.62, Female: IRR = 0.69 95% CI 0.66–0.72)	Based on hospital care provided, cancer incidence was lower among people with ID than in the general population
Tretarre,B ‘17^6^	978 Females	20–85+ years		2009	France	Breast Cancer	20/978 females were diagnosed with any type of cancer, of which 14 were diagnosed with breast cancer, and remaining one colorectal cancer; one uterine and four unspecified localization. The cumulative risk of developing breast cancer <50 years in the ID population was 2.03% (95% CI 0.4–3.66) was not significantly different from the general population: 2.4% (95% CI 1.0%–3.78%)	This study demonstrate that breast cancer occurs and develops as frequent and around the same age as in people without ID
Sullivan,S ‘03^7^	20	50–69 years	Community‐based sample	1982–2000	Australia	Breast cancer	Breast cancer incidence was 64.0 per 100.000 persons‐years among people with ID versus 146.7 per 100.000 person‐years in the general population. With an IRR 64.0/146.7 = 0.43 per 100,000 PYs	Breast cancer risk in this study was reduced as compared to the general population, possibly explained by a shorter life expectancy

**TABLE 2:** Overview of cancer rate in people with specific ID‐related disorders.

Study	ID disorder	No. of participants	Age of the included cohorts (*y*, mean/median or range)	Study design	Time periods data collected	Population (country)	Type of cancer	Cancer incidence among people with ID	Recommendations on screening/general conclusion
Lawania, S. ‘18^26^	Xeroderma pigmentosum A	370	22–86 years	Case–control study		India	Lung cancer	Subjects with variant genotype (GG) for A23G polymorphism had an elevated risk for lung cancer (OR = 2.56; 95% CI 1.49–4.39; *p* = 0.0007). For XPA codon 228 polymorphism, the heterozygous genotype (GA) showed a significant protective effect (OR = 0.54; 95% CI 0.38–0.78; *p* = 0.001)	XPA A23G polymorphism might be associated with increased lung cancer risk in variant genotype, with XPA G709A polymorphism functions as a protective effect for lung cancer (for nonsmokers) in the North Indian population
Paszkowska‐Szczur, K. ‘13^27^	Xeroderma Pigmentosum A–G	714	Mean age in patients, female: 63 and male: 65.5	Case–control study	2002–2006	Poland	Melanoma	The XPC rs2228000 CT (OR = 0.15 95%CI 0.07–0.29; *p* < 0.001) and TT (OR 0.11; 95% CI 0.03–0.37; *p* < 0.001) had decreased melanoma risks compared to the reference genotype (CC). Analogously, XPC G1475A_AG genotype and XPD rs238406_AC genotype were associated with significantly increased risk of melanoma (OR 3.54; 95% CI 1.18–12.6; *p* < 0.05 and OR 2.38; 95% CI 1.14–4.96; *p* < 0.05, respectively) in comparison with the reference genotypes	CT/TT genotype in rs222800 (XPC Ala499Val) could be a protective factor against melanoma risk. The other XP genes do not appear to be associated to any significant degree with melanoma development. The outcomes from this study supports the concept that only XPC and XPD genes are associated with melanoma susceptibility
Shao,J ‘07^28^	Xeroderma pigmentosum group D	215	Mean age in patients and control: 62.9	Case–control study	2003–2005	China	Bladder cancer	The A allele of XPD Arg156Arg (C22541A) and the C allele of XPD Lys751Gln (A35931C) are associated with increased risk of bladder cancer (adjusted OR = 1.54, 95% CI 1.19–2.01 and 1.65, 1.12–2.73 respectively). XPD156, AA genotype showed a significant increase for bladder cancer versus CC and AC genotype (adjusted OR = 1.58, 95% CI 1.20–2.08). For XPD751, AC genotype showed a significant increased bladder cancer risk versus the homozygous wild genotype (AA adjusted OR = 1.86 95% CI 1.13–3.04)	The A allele of XPD Arg156Arg (C22541A) and the C allele of XPD Lys751Gln (A35931C) are related with an increased risk of bladder cancer
Xie,Y ‘21^29^	Xeroderma pigmentosum group A	500	≤50–>60 years	Case–control study	2008–2013	China	Gastric Cancer	For xeroderma pigmentosum group A‐binding protein (XAB2) rs794078 polymorphism, the AA genotype carriers had a significantly lower risk for gastric cancer (OR = 0.33; 95% CI 0.12–0.91) versus GG genotype. The rs794078 heterozygous AG showed no association with the risk of gastric cancer (OR = 1.29; 95% CI 0.95–1.76)	The XAB2 rs794078AA genotype can behave as a protective factor of gastric cancer. XAB2 polymorphism could contribute as a biomarker as risk prediction of gastric cancer in the future
Yang, Z ‘08^30^	Xeroderma pigmentosum group C	153	Mean age in patients: 48.7	Case–control study	2005–2006	China	Nasopharyngeal carcinoma	Carriers of 499Val have a significant risk for NPC compared to non‐carriers (OR = 1.603; 95% CI 1.160–2.216; *p* = 0.005). The 599Val/939Lys/PAT‐(TA–) haplotype is associated with a significantly higher rate than in the controls (OR = 1.901, 95% CI 1.284–2.814, *p* = 0.002)	This study showed us that XPC codon, polymorphism 499Val Allele and its haplotype (499Val/939Lys/PA‐(TA‐)) are associated with significant increased risk for NPC. Implying that Val499Ala polymorphism could be involved in development of NPC
Yuan, T ‘11^31^	Xeroderma Pigmentosum group D	190	30–84	Case–control study	2006–2010	China	Gastric cancer	The OR value for XPD 312Asn was 0.73 (95% CI 0.14–2.72)	In this study, XPD Asp312Asn was not found to be associated with gastric cancer risks
Samson,M. ‘11^32^	Xeroderma pigmentosum group D	250		Case–control study		India	Breast Cancer	XPD 751 Gln/Gln genotype depicts a significant increased risk for breast cancer (OR = 1.7, 95% CI 1.02–2.8), particularly in premenopausal female patients (OR = 2.6; 95% CI 1.33–4.79)	XPD 751 (Gln/Gln) may play a role in the predisposition to breast cancer risk. Especially in premenopausal woman, who are in a constant exposure of steroid hormones and other environmental carcinogens to breast cell occurs
Jorgensen,T ‘06^33^	Xeroderma pigmentosum group A–G	321	Mean age in cases: 56.8 versus control: 56.6	Case–control study	1992–1996	USA	Breast cancer	No significant associations were found between breast cancer risk and any of the XP genotypes: XPC, XPD, XPF, and XPG, with ORs between 0.61 and 1.14	Polymorphism of the XP genes does not seem to have a significant risk for breast cancer in woman
Chang,C, ‘09^34^	Xeroderma pigmentosum group D	308		Case–control study	2001–2008	Taiwan	Bladder cancer	The A allele at XPD Asp312Asn conferred a 1.85‐fold risk factor for bladder cancer (95% CI 1.34–2.56)	The A allele of the XPD codon 312 may be responsible for bladder carcinogenesis and useful in the early detection and prediction of bladder cancer
Cui,Y ‘06^35^	Xeroderma pigmentosum group G	611/601	17–>54	Case–control study	1999–2004	USA	Lung cancer/oropharyngeal cancer	The XPG Asp1104Asp genotype was associated with a decreased risk of squamous cell carcinomas of the oropharyngeal oropharynx, larynxgeal, and oesophaguseal cancers (SCCOLE) (OR = 0.47, 95% CI 0.27–0.82) and for lung cancer (OR = 0.62, 95% CI 0.38–1.0)	The study showed that XPG Asp1104Asp genotype was associated with a decreased risk of developing lung cancer and squamous cell carcinomas of the oropharynx, larynx, and esophagus. Signifying protective effects on these malignancies
Jeon,H ‘03^36^	Xeroderma pigmentosum group G	310	Mean age case: 61.5 versus control: 60.5	Case–control study	1999–2000	Korea	Lung cancer	The XPG codon 1104 Asp/Asp is linked with decreased risk for lung cancer (OR = 0.54, 95% CI 0.37–0.8). Among the lung cancer cases, genotype His/Asp was more frequent than in the controls, while genotype Asp/Asp was lower. The XPG codon 1104 Asp/Asp genotype was associated with a significantly decreased risk of lung cancer (adjusted OR = 0.54, 95% CI 0.37–0.80), compared to the combined His/ His and His/Asp genotype as reference group	The Asp/Asp genotype may be a protective genotype for lung cancer
Miller,K ‘06^37^	Xeroderma pigmentosum group A	886/682	Mean age: 61.2	Case–control study	1993–1995/1997–2000	USA	Basal cell carcinoma (BCC)/squamous cell carcinoma (SCC)	Using GG as the reference, the A allele was less frequent among cases of BCC, OR A/G = 0.82 (95% CI (0.66–1.01); OR A/A = 0.74 (95% CI 0.53–1.03); and SCC OR A/G = 0.85 (95% CI 0.67–1.07); OR A/A = 0.74 (95% CI 0.52–1.05) than controls	The common G allele within the XPA A23G polymorphism is linked with an increased risk for BCC and SCC, and this polymorphism appeared to be the determining polymorphism in XPA that alters cancer susceptibility
Bjorge,T ‘08^38^	Down's syndrome	2108/3201 with DS. Altogether, 5.2 million children and their families were included	0–39	Population‐based cohort study	1967/1973 to 2004	Norway/Sweden	All cancers	The risk of cancer in individuals with Down's syndrome was increased in both Norway and Sweden. The risk of leukemias was most strongly elevated (SIRs between 15 and 141), with all cases occurring <5 years of age. In Norway, the risk of testicular cancer was elevated in males with Down's syndrome SIR 5.5 (95% CI 1.8–13)	An increased overall cancer risk in individuals with birth defects was observed. The highest risks were seen for individuals with malformations in the nervous system, Down's syndrome, and multiple defects
Boker,L ‘01^39^	Down's syndrome	2635	0–17	Population‐based cohort study	1948–1995	Israel	All cancers	Cancer was observed more frequent than expected among DS individuals born before 1979 (SIR = 1.33 95% CI 0.77–2.12) and after 1979 (SIR = 4.67 95% CI 1.90–9.60), with leukemias observed most (<1979 SIR = 6.90 95% CI 1.90–17.70; and >1979 SIR = 25.18 95% CI 10.40–53.40, respectively)	These results confirm statistically significant excess rate of leukemia in the DS population in Israel
Choi,Y ‘22^40^	Down's syndrome	Patients with acute leukemia at diagnosis between; 54 DS and 4643 non‐DS 0‐19y: 54 DS Children born from 2007 to 2008: DS: 397	0–19	Population‐based cohort study	2006–2016/2007 and 2008	Korea	Acute leukemia	The relative risk of acute leukemia, ALL, and AML was 49.25, 20.75, and 163.38 times higher in the DS group than in the non‐DS group, respectively	The incidence of acute leukemia among patients with DS is higher than among those without DS in Korea
Goldacre,M ‘04^41^	Down's syndrome	1453	0–59	Population‐based cohort study	1963–1999	England	All cancers	Leukemia risk was higher in people with DS versus the control group (rate ratio 19.0, 95% CI 10.4–31.5). The risk ratio for other cancers, excluding leukemia, was 1.2 (95% CI 0.6–2.2). Testicular cancer was observed more than expected (RR 12.0 95% CI 2.5–35.6), but based on 3 cases	People with ID are at a higher risk for leukemia (with similarly high risks for AML and ALL) in DS and testicular cancer
Hasle,H. ‘16^10^	Down's syndrome	3530	0–≥60	Population‐based cohort study	1961–2007	Denmark	All cancers	Overall risk for all solid tumors, SIR = 0.45, 95% CI 0.34–0.59. Only testicular cancer had an increased, SIR = 2.9, 95% CI 1.6–4.8	The risk for solid tumors was decreased, except testicular cancer. Altered screening strategies should be considered for persons with DS
Hasle,H ‘00 (Lancet)^9^	Down's syndrome	2814	0–≥60	Population‐based cohort with Record linkage	1961–1994	Denmark	All cancers	The overall incidence of cancer (60 cases observed) was not significantly different from what was expected (49.8 cases), SIR = 1.20, 95% CI 0.92–1.55. Leukemias constituted 60% of the cases of malignant diseases overall and 97% in children below 15 years of age (SIR = 17.63, 95% CI 12.4–24.4). The SIR for solid tumors was decreased in both children and adults (SIR = 0.50, 95% CI 0.32–0.75)	The occurrence of cancer in Down's syndrome is unique, with a high risk of leukemia in children and a decreased risk of solid tumors in all age‐groups. The distinctive pattern of malignant diseases may provide clues in the search for leukemogenic genes and tumor suppressor genes on Chromosome 21
Hill,D ‘03^42^	Down's syndrome	4872	1–≥50	Population‐based cohort with Record linkage study	1965–1993	Denmark/Sweden	All cancers	All cancers: 67 (Observed), SIR = 1.7 (95% CI 1.3–2.1). Of which 36 (54%) were diagnosed with acute leukemias (all leukemias SIR = 19.5, 95% CI 13.7–26.8). Risks of testicular cancer (SIR = 3.7, 95% CI 1.0–9.4; *n* = 4), penile cancer (SIR = 45.5, 95% CI 9.3–132.8), and liver cancer (SIR = 6.0, 95% CI, 1.2–17.5; *n* = 3) were also elevated	In this cohort an elevated risk for leukemia and testicular, penile and liver cancers was detected among people with DS compared to the general population
Marlow, E'21^43^	Down's syndrome	4401 DS children and 3.9 million other children	0–14 (at end of follow‐up)	Population‐based cohort with Record linkage	1996–2016	USA/Canada	Leukemia	124/4401 (2.8%) of the children with DS were diagnosed with leukemia. Risks were highest in children with DS <5 years of age: overall leukemia – HR = 75.6 (95% CI 62.3–91.6); ALL – HR = 28.4 (95% CI 20.4–39.6) and AML – HR = 399 (95% CI 281–566)	DS is a strong risk factor for childhood leukemia, with associations of AML being stronger than earlier reported
Patja,K ‘06^44^	Down's syndrome	3581	0–79	Population‐based cohort study	1978–1986	Finland	All cancers	The risk for any cancer was similar between people with DS and the general population (SIR = 0.9, 95% CI 0.7–1.1). Risks were elevated for leukemia (SIR = 10.5 95% CI 6.6–15.8), and testis cancer (SIR = 4.8, 95% CI 1.08–10.4). Reduced risks were observed for most other (solid) tumors	Although the risk of cancer among persons with DS is equal with the general population, there is a large excess risk of leukemia, and some excess of testicular cancer, while incidence of solid tumors is lower. The cancer profile of individuals with DS considers the genetic background and specific risk factors, yet in cancers which are associated to behavioral risk factors, the health behavior factors can lead to a reduced incidence of other type of cancers
Sullivan,S ‘07^45^	Down's syndrome	1298	Mean age at start of cohort 20.2	Population‐based cohort with Record linkage	1982–2001	Australia	All cancers	The risk for any cancer was similar between people with DS and the general population (SIR = 1.10 95% CI 0.68–1.68). Leukemia was most prevalent (62% of all cases, SIR = 8.42, 95% CI 4.48–14.4), in particular at ages <5 year (SIR = 61.61 95% CI 31.84–107.62). Lower risks were observed for most other (solid) tumors (SIR = 0.44 95% CI 0.19–0.86)	Overall incidence of cancer among people with DS was not significantly different from the general population. However incidence of leukemia was significantly higher, in particular for young children with DS. Incidence of solid tumors was reduced
Agha,M/M ‘04^46^	Down's syndrome	45,200 children with congenital abnormalities who were matched with 45,200 children without congenital abnormalities	Mean age 26.7	Population‐based cohort with record linkage	1979–1996	Canada	All cancers	Children with congenital abnormalities were found to have a significantly higher risk of developing some type of cancer, including leukemia, tumors of the CNS, tumors of the sympathetic nervous system, and soft tissue sarcomas (RR = 2.0 95% CI 1.8–2.4). A strong association found between DS and leukemia (RR of 38.6) and ANLL (RR of 104.9), however, within the cohort of children with congenital abnormalities, there was no significant difference in the distribution of cancer types (*p* = 0.55)	This study confirms the association of DS and leukemia, but also showed this association for children with other abnormalities. Congenital anomalies may be markers of other exposures or processes that increase the risk of childhood cancer. The identification of these cancer‐prone syndromes should spur an increased effort to understand the underlying links between these syndromes and the development of cancer
Madanikia,S ‘12^47^	NF1	126	24–28	Chart review	2003–2010	USA	Breast Cancer	4/126 (3.2%) of the NF1 patients had breast cancer. For woman <50 the (adjusted) SIR was 4.41 (95% CI 1.12–12)	The outcome of this study suggests an increased risk of breast cancer for women with NF1 < 50 years old and the need for early and annual of radiographic screening for breast cancer in NF1 patients. However, more research is needed to determine the optimal screening program and timing
Marjanksa,A ‘20^48^	NF1	830	0–82	Chart review	1999–2018	Poland	All cancers	The prevalence of any malignancy in this NF1 population was 289/830 (34.8%), (OR = 23, 95% CI 18–29). Especially tumors were found within the optic pathway glioma and plexiform neurofibroma. One hematological malignancy was found	NF1‐individuals develop malignancies earlier when compared to the general population. These patients have a high risk of developing various types of cancer, and the course of treatment is difficult due to the numerous diseases associated with NF1
Landry J,’21^49^	NF1	1607	0–83	Cohort study	1985–2020	USA	All cancers	666/1607 patients with NF1 developed other neoplasms in addition to neurofibromas (41.4%; OR = 9.5; 95% CI, 8.5–10.5). Moreover, an elevated risk for melanoma (OR = 3.9; 95% CI, 2.4–6.5); ovarian cancer (OR = 5.6; 95% CI, 2.8–11.1); breast cancer (OR 3.8; 95% CI 2.9–5.1) was reported	Findings suggest that patients with NF1 develop several neoplasms, including gliomas and sarcomas, at a younger age, more frequently, and with worse outcomes compared with individuals without NF1 and that counseling and annual follow‐up should be advised for these patients. Mammography screening should begin at age 30 in women with NF1, and potential benefits of (early) ovarian cancer screening should be investigated. As for melanoma, a high index of suspicion for melanoma in patients with NF1 should be maintained, with frequent meticulous skin and ocular exams
Seminog, O. ‘15^50^	NF1	3672	30–79	Population‐based cohort with Record linkage	1999–2011	England	Breast cancer	58/3672 NF1‐patients (1.6%) were diagnosed with breast cancer during study period. A total of 25 patients were <50 years old and 33 pt were >50 years old. Relative risks were highest for women aged 30–39 (RR 6.5 95% CI 2.6–13.5), and declined with increasing age (60–69 RR 1.9 95% CI 1.0–3.3)	An excess risk of breast cancer in young woman with NF1 was observed, which might have implications for early breast cancer screening. If surveillance of breast cancer in women with NF1 is considered, there may be a case for screening from 30 years of age. However, the benefits of a screening program need to be carefully weighed against the risk of exposing young women with NF1, which is a tumor‐suppressor syndrome, to radiation
Walker,L ‘06^51^	NF1	448	0–72	Prospective cohort study	1990–2004	United Kingdom	All cancers	36/448 individuals in the cohort had a malignant tumor during follow‐up (SIR = 2.7; 95% CI 1.9–3.7). The most observed types of cancer were connective tissue tumors (SIR = 122 95% CI 61.0–219) and brain tumors (SIR = 22.6 95% CI 9.1–46.5), which are rare in the general population. At the age < 50, breast cancer risk was significantly higher as well (SIR = 4.0, 95% CI 1.1–10.3)	The overall risk of cancer was increased by a factor of 2.7 in NF1 patients, mostly because of the increased risk of brain /CNS and connective tissue tumors. The potentially early onset of breast cancer has implications for breast cancer screening programs
Wang,X ‘12^52^	NF1	76 women	30–≥50	Chart review with data linkage	1990–2009	USA	Breast cancer	9/76 women with NF1 were identified with initial invasive, where 1.8 cases were expected (SIR = 5.2; 95% CI 2.4–9.8). 6 cases were <50 years of age (0.7 expected; SIR = 8.8; 95% CI 3.2–19.2)	These data are consistent with other reports suggesting an increase in breast cancer risk among females with NF1, which indicate that breast cancer screening guidelines should be evaluated for this potentially high‐risk group
Uusitalo,E ‘16^53^	NF1	1404	0–50		1987–2012	Finland	All cancers	A total of 244 cases of cancer were observed while 48.5 cases were expected (SIR = 5.03 95% CI 4.42–5.71). The lifetime risk of cancer in NF1 was 59.6% versus 30.8% in the overall population. Besides peripheral nerve sheath tumors and CNS tumors, the cancers traditionally associated with NF1, SIR were elevated for breast, and digestive cancer, Moreover, breast cancer incidence was significantly increased in woman <40 years old (SIR = 11.1 95% CI, 5.56–19.5)	Cancer in patients with NF1 manifests in multiple and previously unrecognized ways. The accumulation of mutations that lead to malignancy may occur earlier in patients with NF1 than in control populations, leading to the increased cancer risk of more cancer types than previously have been observed. These findings should translate to clinical practices to determine clinical interventions and focussed follow‐up of patients with NF1
Uusitalo,E ‘17^54^	NF1	1404 (same population as described prior)	28–84	Population‐based cohort with Record linkage	1987–2011	Finland	Breast cancer	46/1404 NF1 patients were diagnosed with breast cancer, including 1 male. Women <40 were at particular high risk (SIR = 14.3 95% CI 6.5–27.0), and the SIR of breast cancer was not significantly increased in the age groups of >50 years	Results show that the breast cancer incidence in NF1 women aged <40 years depicts an increased risk when compared to the general population, which highlight the question of the surveillance strategy for NF1 breast cancer. The following is suggested: (1) increasing the awareness of NF1 patients and their doctors of the high breast cancer risk especially in young women with NF1, (2) yearly clinical follow‐up since young adulthood by doctor familiar with NF1 syndrome, and (3) any suspicion of breast cancer should warrant imaging
Kutler,D ‘03^55^	Fanconi anemia	754	14–48	Cohort study		USA	Head and neck squamous cell carcinoma (HNSCC)	19/754 (3%) FA patients were diagnosed with HNSCC (SIR = 500, 95% CI 300–781). The cumulative incidence of developing HNSCC by the age of 40 years was 14%	Patients with FA have an increase incidence of aggressive HNSCC, that frequently develops at an early age and has a poor prognosis. Because of the complex nature of the treatment for FA‐associated HNSCC, careful screening of the head and neck in patients with FA is essential to discover oral cavity lesions at an early stage. Based on the age distribution in this study, biannual screening of the oral cavity and oropharynx should start between the ages of 15 and 20 years. Yet, in patients with FA with a history of leukoplakia or recurrent oral lesions, head and neck examinations are recommended every 6–8 weeks
Rosenberg,P ‘03^56^	Fanconi anemia	145	0–49	Survey‐based study	2000	USA	All cancers	23/145 questionnaire responders with FA reported a history of cancer. The ratio of observed to expected cancers (O/E ratio) was 50 for all cancers, 48 for all solid tumors, and 785 for leukemia, all with *p* < 0.05	In addition to their well‐known risk of AML persons with FA have an extraordinary risk of developing a solid tumor. Current and future generations of patients with FA will require rigorous and lifelong screening for solid tumors regardless of their severity of their anemia or the origin of their bone marrow
Rosenberg, P ‘08^57^	Fanconi anemia	181		Registry‐based cohort	1984–2006	Germany	All cancers	30 cancers were observed among 181 FA patients (O/E ratio 44). 12 were solid tumors (O/E ratio 18), and 18 were hematological of which 17 concerned leukemia (AML) cases (O/E ratio 868)	Fanconi anemia is a highly penetrant cancer susceptibility syndrome with early onset of acute myeloid leukemia and slightly later onset of specific solid tumors. These findings, together with major advances in the treatment of bone marrow failure, strongly suggest that solid tumors will become the predominant clinical problem of patients with Fanconi anemia
Dutzmann,C ‘22^58^	Fanconi anemia	421	0–48	Registry‐based cohort study	1980–2020	Germany	All cancers	In total 33/421 childhood cancer were diagnosed in the FA population compared to expected 0.74 (SIR = 39; 95% CI, 26–56). The highest cancer risk were observed in patients with biallelic mutations: FANCD1 (BRCA2; SIR = 324; 95% CI, 88–830) or FANCN (PALB2; SIR = 422; 95%CI, 51–1526), while no neoplasms were observed in patients (*n* = 20) with biallelic mutations: FANCD2. The cumulative cancer risk before age 18 years was 10.6% in the entire FA cohort	Roughly, 11% of patients with FA develop cancer before the age of 18. FA for childhood cancer screening and surveillance already exist for these children and are supported by our results
Schultz‐Pederson. A. ‘01^59^	Fragile X syndrome	223	0–84	Registry‐based cohort study	1977–1996	Denmark	All cancers	4/223 (1.7%) individuals were diagnosed with a form of cancer, of which one was diagnose with cancer before the diagnosis of fragile X (SIR = 0.28 95% CI 0.1–0.8)	Outcomes suggest a decreased risk for cancer in people with fragile X. The lower risk of some cancer types may be due to limited exposure to occupational carcinogens, alcohol, and tobacco. Further studies are needed to investigate genetic mechanisms that protect against malignant transformation
Sund,R ‘09^60^	Fragile X syndrome	302	0–≥80	Population‐based cohort study	1982–1986	Finland	All cancers	11 cancer cases were observed, where 13.8 were expected (SIR = 0.8 95%CI 0.4–1.4). Cancers of the mouth and pharynx were observed more than expected (SIR = 8.1 95% CI 1.7–24)	No statistically significant protective effect for cancer was detected in this cohort of individuals with fragile X syndrome. Mechanisms linking fragile X syndrome, and cancer needs further research
Roznowski, K,’08^61^	Nijmegen breakage syndrome	270		Case–control		Poland	Breast cancer	The NBS1 gene mutations were overrepresented among breast cancer patients compared to controls (2.59% vs. 1.02%). In particular, the I171V mutation was significantly more present in the breast cancer group than in the control group (OR = 9.42; 95% CI 1.09–81.05; *p* = 0.02)	Carriers of the heterozygous mutation I171V in could be at increased breast cancer risk. Furthermore, genetic consulting detection of heterozygous carriers of I171V mutation in first‐degree relatives of breast cancer patients is recommended
Bogdanova,N ‘08^62^	Nijmegen breakage syndrome	1012/1588	Median age at onset of breast cancer: 57	Case–control study	1996–1999	Germany/Belarus	Breast cancer	In the combined samples, data shows a significant association between 6756del5 mutation and breast cancer development (*p* < 0.01) whereas an effect was not detected for R215W substitution (*p* = 0.18)	675del5 mutation is associated with an increased risk for breast cancer, and the R251W substitution may represent a cancer susceptibility allele with reduced penetrance. The NBS1 gene can be added to the growing list of genes involved in DNA double strand break repair which, if mutated, confer to a 2‐ to 4‐fold increased risk for breast cancer
Sugrañes,T ‘22^63^	Bloom syndrome	290	Median age at diagnosis: 7.8	Registry study	1996–2021	USA	All cancers	155/290 (53%) registry participants with BS were diagnosed with 251 malignant neoplasms. The cumulative incidence of any malignancy by age 40 was 83%. Hematological malignancies were most frequent. The O/E ratios were 631 for all cancers, 3625 for leukemia, 1931 for lymphoma, 479 for breast cancer, and 1847 for colorectal cancer	People with BS are diagnosed with cancer more frequently and at earlier ages. The data provide strong support for implementation of cancer surveillance strategies for early detection of initial subsequent malignancies, at much younger ages than recommended in the healthy population
Boot,M ‘18^64^	Rubinstein–Taybi syndrome (RTS)	87	2–69	Population‐based cohort with record linkage	1986–2015	Netherlands	All cancers	35 tumor diagnoses (benign and malignant) were identified in 26/87 individuals with RSTS, of which 5 malignant tumors in four persons (medulloblastoma; diffuse large‐cell B‐cell lymphoma; breast cancer; non‐small cell lung carcinoma; colon carcinoma)	Within this small population, and additional literature, an increased risk for malignant tumors could not be verified. A specific surveillance in children or adults with RTS to detect tumors at an earlier age is not indicated based on presently available data
Davies,H ‘03^65^	Prader–Willi syndrome (PWS)	1077 with PWS	5–30	Survey‐based study	1994	USA	All cancers	35/1077 persons with PWS had a history of a benign tumor or cancer (malignant tumor or leukemia), of which 8 were confirmed malignant cancers (4.8 expected; O/E ratio = 1.67 95% CI 0.72–3.28) and 3 cases were leukemia (0.08 expected; O/E Ratio = 40.18 95% CI 8.0–117)	Among PWS individuals an excess of myeloid leukemia was observed in this study population. It would seem that consideration should be given to adding PWS to the list of genetic disorders that predisposes to leukemia
Greene,A. ‘04^66^	Klippel–Trenaunay syndrome (KTS)	115 with KTS/8614 with Wilms tumor	Average age: 14.3	Database study	1999–2000	USA/National Wilms Tumor Study Group	Wilms tumor	0 patients were diagnosed with Wilms Tumor in both series	Even with the most conservative estimates, the likelihood of Wilms tumor and KTS in the same patient is very small. No routine screening recommended for Wilms Tumor in KTS
Sahlin,J ‘07^67^	Saethre–Chotzen syndrome	65 individuals affected by the syndrome		Interviews	1996–2001	Sweden	Breast cancer	15/29 carriers, >25 years of age had developed breast cancer (0.89 expected; SIR = 16.80 95% CI 1.54–32.06). Most of these individuals were of young age, implying early onset of breast cancer development	Within this study population a high prevalence of breast cancer was identified. These outcomes strongly imply that individuals with Saethre–Chotzen syndrome would benefit from genetic counseling and enrolment in surveillance programs including yearly mammography
Heald, B ‘10^68^	PTEN–associated Cowden syndrome	127 with PTEN mutation	1–73	Prospective biobank	2005–2009	USA	Colorectal adenocarcinomas	62/127 subjects had colorectal polyps. Nine of these people had colorectal adenocarcinomas, all under the age of 50 (SIR = 224.1 95% CI 109.3–411.3)	PTEN mutation, associated with CS must be considered as mixed polyp syndrome, where hyperplastic polyps are most prevalent, subsequently with a risk of early‐onset colorectal cancer. Therefore, routine colonoscopy should be considered in this population
Kratz,C ‘15^69^	Noonan syndrome/Costello syndrome	632 with NS and CS with 32	0–14	Registry‐based cohort study	1980–2012	Germany	All cancers	8/632 NS patients developed cancer (1.0 expected (SIR = 8.1, 95% CI 3.5–16.0), and 2/32 CS patients (0.05 expected; SIR = 42.4, 95% CI 5.1–153.2)	In this study, there was a significant excess risk for all childhood cancers in both syndromes as compared to the general population. However, this study does not emphasize the benefits for clinical cancer screening in people with RASopathies (including Noonan and Costello syndrome)
Peron,A ‘16^70^	Tuberous sclerosis	240	0–74	Cohort study	2001–2015	Italy	All cancers	15/240 individuals had a history of malignancies. Seven of these patients had renal carcinoma, the remaining 8 had uterine (*n* = 2) and colon carcinoma (=2), or abdominal sarcoma (*n* = 1), osteosarcoma (*n* = 1); squamous cell carcinoma of the skin (*n* = 1) and lioblastoma multiforme (*n* = 1). Based on comparison with national Italian statistics. The prevalence of non‐renal malignancies overlaps with the prevalence in the general Italian population (2.6% vs. 4.4% in Italy), and the prevalence of all malignant tumors in the TSC population is compatible with the one in the general Italian population (5.6%, 95% CI 2.99–9.31% vs. 4.4% in Italy)	TSC patients do not seem to have an increased risk for malignancies, besides renal cell carcinomas. Reason why renal cancer is the most prevalent malignancy in TSC still remains to be elucidated. Authors do not recommend routine screening for malignancies in TSC patients
Jiang, J'16^71^	Sex‐development disorder (containing Y‐chr material)	202	16–42	Retrospective cohort (1992–2015)	1992–2015	China	Gonadal tumor development	37 out of the 202 (18.3%) had gonadal malignancies during follow‐up	Authors recommend performing immediate prophylactic gonadectomies upon diagnosing adult female patients with disorders of sex development, such that their karyotypes contain Y‐chromosome material

### 3.2 ID population

Of the 55 included articles, 7 studies focused on the overall population of patients with ID, and all but one included a population of more than 900 individuals with ID. In 4 of the 7 papers, people with ID were found to have a lower cancer incidence rate than the general population,^5^, ^24^, ^73^, ^74^ while two reported a similar incidence rate.^22^, ^75^ The most recent study, published by Liu et al. in 2022,^23^ reported a significantly higher rate of cancer among individuals with ID compared with those without ID (Table 1).

### 3.3 ID‐related disorders

A total of 48 papers described the cancer rate in people with specific ID‐related disorders (Table 2).

Genetic variations of the XP genes have been studied for their risks for various cancer types. Across the XP groups A–G, there was found no association with a risk for breast cancer,^33^ one study reports that variations within XPD can play a role in the predisposition to breast cancer risk, especially in premenopausal woman.^32^ Similarly, different genetic variations within XPC and XPD were associated with melanoma susceptibility, while one mutation appeared as a protective factor against melanoma.^27^ For the remaining XP genes no significant risks for melanoma was found.^27^ Different variations within XPA reported an increased risk for lung cancer,^26^ basal cell carcinoma, and squamous cell carcinoma.^37^ While other genetic mutations within XPA, appeared to serve as protective factors against lung cancer (for nonsmokers) and gastric cancer.^26^, ^29^ Different variation within XPC were reported to play a role in the development of nasopharyngeal carcinoma.^30^ According to Shao et al, certain variations of the XPD gene are linked to higher risk for bladder cancer.^28^ Additionally, one research group described that there was no correlation between a different variation within XPD and the development of gastric cancer.^76^ The presence of a specific genetic mutation within XPG is associated with a reduced link to lung cancer squamous cell carcinomas in the throat and digestive system (oropharynx, larynx, and esophagus).^35^

Regarding individuals with DS, four studies reported a higher,^38^, ^39^, ^41^, ^42^ and three studies a similar^9^, ^44^, ^45^ cancer rate as observed in the general population. Results on specific cancer types showed the incidence of solid tumors, except for testicular cancer, to be lower among people with DS,^9^, ^10^, ^44^ while leukemia was significantly more common^39^, ^45^ than the general population, especially at younger ages.

In six of eight papers higher risk for earlier breast cancer was diagnosed in woman with NF1,^47^, ^49^, ^50^, ^52^, ^54^, ^77^ the other two papers report the higher risk of developing any type of cancer.^48^, ^53^

The three included studies on FA show that people with this syndrome are susceptible to the early onset of acute myeloid leukemia.^55^, ^57^, ^58^ In addition, two of these studies described an extraordinary risk for the development of solid tumors as well.^55^, ^56^

Individuals with FXS had a lower prevalence of cancer overall than the general population.^59^, ^60^

For nine ID‐related syndromes, only one article each was identified. In BS, the cancer incidence was increased, especially in younger people.^63^ Breast cancer was more prevalent in people with Saethre–Chotzen syndrome compared with the general population.^67^ Prader–Willi syndrome was linked to a higher incidence of myeloid leukemia.^65^ People with Noonan and Costello syndromes had higher prevalence of childhood cancer (leukemia and solid tumors),^69^ while individuals with a PTEN mutation and Cowden syndrome have a higher risk of early‐onset colorectal cancer. For Rubinstein–Taybi syndrome, Klippel–Trenaunay syndrome or tuberous sclerosis, no increased risk of cancer was described.^64^, ^66^, ^70^ A study by Jiang et al. reported that the incidence for gonadal malignancies increased with age for females carrying Y‐chromosome material.^71^

### 3.4 Recommendations for cancer screening

Of the seven articles on cancer in DS, one study suggested the recommendation for general alteration in the screening strategies used in this group, with no explicit form of recommendation.^10^

Earlier breast cancer screening than in the general population is recommended for females with NF1 as studies consistently show a higher incidence and at younger ages.^11^, ^47^, ^49^, ^52^, ^53^, ^54^, ^77^ Surveillance and screening programs are available for children with FA,^58^ one study highlights a need for heightened cancer surveillance among adults individuals with FA as well.^55^

Sugrañes et al.^63^ recommended to initiate cancer screening earlier in people with BS than current guidelines suggest. Sahlin et al.^67^ implied that people with Saethre–Chotzen syndrome would benefit from genetic counseling and yearly breast cancer screening because of their higher risk of breast cancer.^67^ To mitigate the risk for early‐onset colorectal cancer in individuals with Cowden syndrome and a PTEN mutation routine colonoscopies should be considered as existing screening programs would not suffice.^68^ Although both childhood leukemia and solid tumors were found to be increased among individuals with Noonan and Costello syndrome there was evidence to support the effectiveness of specific cancer screening measures for these individuals.^69^

The studies focusing on genetic variations within ID‐related disorders, generally contain useful information for genetic counseling and tumor development predictors. Particularly, Chang^34^ described that, within XPD, the allele A of codon 312 could be used for the early detection and prediction of bladder cancer. Carriers of the I1717V mutation in people with NBS have a higher risk of breast cancer, and it has been proposed that first‐degree relatives could benefit from genetic counseling.^61^ Finally, adult females with Y‐chromosome material should be considered for immediate prophylactic gonadectomies.^71^

The studies with a specific focus on genetic variations in relation to cancer risk, identified potential predictors for tumor development. In particular, in XPD, NBS, and sex development disorders, genetic counseling is recommended to advice on the need for cancer screening.^34^, ^61^, ^71^

## 4 DISCUSSION

Our review aimed to comprehensively review the available literature on cancer risks and their implications for cancer screening in the patient population with ID, as well as within subgroups based on specific ID‐related disorders or genetic predispositions. With a main finding that studies investigating cancer incidence and prevalence where in specific ID‐related disorders reported a higher cancer rate, while studies in the general ID population reported a lower or similar rate to the general population. Based on our results, we advise a more tailored and personalized, approach in cancer screening within the ID population.

The general ID‐population studies included large groups of individuals with ID (at least 900 individuals, expect in one study), without specifying the ID etiology or disorder, and had comparison groups from the general population without ID. Most studies (6/7) reported a lower or similar prevalence of cancer among people with ID in comparison with the general population. One possible explanation for finding a lower incidence rate in the ID population studies is that, in the majority of population‐based studies, a large proportion of people with DS are represented, being the largest syndromic ID subgroup; however, people with DS are known to have a decreased rate of solid tumors,^9^, ^10^, ^22^ which may skew the results. Furthermore, in most of the studies age was not standardized. The heterogeneity of the population, including age distributions, can also account for the variations in cancer incidence rate.^19^ Only the most recent population‐based study by Liu et al.^23^ reported an increased risk for any cancer in people with ID. Notably, these articles were published over a broad time span, apart from improved methods for diagnosing ID, and improved longevity of the ID population, this may also indicate cancer risks have changed over time. Nevertheless, these differences highlight the need for further population‐based research to confirm the direction of cancer incidence rates among individuals with ID.

The overview that this review provides on cancer risks and cancer recommendations per ID‐related disorders highlights the need for tailored cancer screening. The need for changes within the cancer screening programs, are also demonstrated by other studies, focusing on participation and outcomes of screening programs among the ID population.^78^, ^79^, ^80^

We have seen that people with DS tend to develop cancer, especially leukemia, at a younger age and a have worse prognosis than the general population.^43^, ^45^, ^81^ Reaffirming the existing awareness for earlier leukemia screening among children with DS,^82^ and recommendations for more specific screening programs for people with DS.^83^

Next to DS, NF1 was frequently (*n* = 8) studied. As mentioned previously, NF1 is a syndrome where not all individuals will necessarily lead to the development of ID. Furthermore, NF1 is associated with a genetic predisposition for the growth of both benign and malignant neoplasms.^53^, ^84^, ^85^ Notably, within our findings, we identified a higher incidence of breast cancer in patients with NF1, which presented with an early onset.^47^, ^50^, ^54^ This indicates a need to initiate breast cancer screening earlier^52^, ^77^; however, this requires a trade‐off between breast cancer risk and excess risk for radiation‐induced malignancies through mammography.^86^ Next, FA is most often associated with leukemia,^87^ the reviewed studies also reported a significantly higher incidence of solid tumors in this population group.^55^, ^56^, ^57^ Specific screening awareness for childhood cancers already exists for patients with FA, by which the results from our included studies further confirming these recommendations. Lastly, our findings do not suggest the need for additional cancer screening is needed for patients with FXS unless they have other risk factors.

In some of those ID‐related studies the focus was emphasized on the association between cancer risks and some genetic variations in people with ID‐related disorders. Mutations for which a relation with tumor development was shown, can serve as predictor for cancer risk and should inform genetic counseling in the need for cancer screening. The findings from this still emerging field shows the relevance of understanding the genetic origins of a person's ID in relation to (preventative) cancer care. Further enhancing genetic knowledge is a crucial step in beating current disparities experienced by people with ID in cancer care. It could, for example, guide the much needed personalized approach in cancer treatment in this vulnerable population.^88^

### 4.1 Limitations and strengths

The starting point for this review was to explore the cancer incidence in people with ID, yet this also included syndromes that do not always result in the development of ID, such as NF1 and xeroderma pigmentosum. A broad definition for these syndromes was thus included, which subsequently meant this was also a strength, a complete picture is included within this review. Although our search strategy identified many case reports (~6500), they were purposely excluded because of their high methodological variety and small sample sizes, which made them less useful for clinical practice. More theme‐specific reviews on these disorders, which include these case reports, and subsequent larger population studies are needed for more accurate answers and implementable guidance for the cancer care of patients with the specific disorders. Still recognizing that some syndromes may be much rarer, making large study populations impossible. We need to acknowledge that the included studies use different approach for the age stratification within the population. It is known that age can be a confounding factor in the ID population, with earlier disease development. Thus, it is recommended that this should be taken into account for follow‐up studies and comparisons. Moreover, while our review included studies on 14 different ID‐related disorders, for 10 ID‐related disorders only one single article met the inclusion criteria. Although these studies provide information about the cancer risks and incidence rate in ID disorders, we need to be cautious in interpreting these results because they may not be representative for each subgroup. Furthermore, due to the high variety in research included within this review, and the exclusion of case reports it might have led to underrepresentation of malignancies in specific groups. For example, tuberous sclerosis is a disease most often associated with benign tumor growth,^89^ but was also reported to be associated with a higher risk for renal cell carcinoma.^90^ Yet, these papers did not meet our inclusion criteria due to their backgrounds (e.g., in vitro studies) nevertheless, earlier case reports and series have suggested the higher prevalence of malignancies within this syndrome.^91^, ^92^ Emphasizing the need for more studies specific ID‐related disorders.

## 5 CONCLUSIONS

The heterogeneity within the ID population challenges precise cancer risk assessment at the population level. Nonetheless, within certain subgroups, such as individuals with specific ID‐related disorders or certain genetic mutations, a more distinct pattern of varying cancer risks compared to the general population becomes apparent. The insights provided can substantially contribute to the clinical and academic field by increasing the awareness on cancer risks and screening in the ID population. This heightened awareness can aid to increase health of people with ID. Additionally, while life expectancies are continuing to rise in the ID population,^93^ more individuals are entering age groups were cancer is more common. This underscores the growing importance of addressing cancer risks and screening within the ID population. Enhanced knowledge of the role of ID within the spectrum of oncology can be instrumental in addressing existing disparities in cancer screening and care experienced by individuals with an ID.

## AUTHOR CONTRIBUTIONS

**Amina Banda:** Conceptualization (equal); data curation (lead); formal analysis (lead); project administration (equal); visualization (equal); writing – original draft (equal); writing – review and editing (equal). **Jenneken Naaldenberg:** Conceptualization (equal); formal analysis (equal); funding acquisition (equal); supervision (equal); writing – review and editing (equal). **Aura Timen:** Conceptualization (equal); formal analysis (equal); supervision (equal); writing – review and editing (equal). **Agnies van Eeghen:** Conceptualization (equal); formal analysis (equal); supervision (equal); writing – review and editing (equal). **Geraline Leusink:** Conceptualization (equal); formal analysis (equal); funding acquisition (equal); supervision (equal); writing – review and editing (equal). **Maarten Cuypers:** Conceptualization (equal); data curation (equal); formal analysis (equal); funding acquisition (equal); supervision (equal); writing – review and editing (equal).

## FUNDING INFORMATION

The study was funded by the Maarden van der Weijden Foundation (Reference: 2021–23); The Netherlands Organisation for Health Research and Development (ZonMw) (Reference: 641001100) and the Ministry of Health, Welfare and Sport (VWS), the Netherlands (Reference: 329574). The funding sources had no role in the study design, data collection, data analysis, data interpretation, or writing of this report.

## Supporting information


**Appendix S1:** xxx.

**Appendix S2:** xxx.

**Appendix S3:** xxx.

## Data Availability

Data sharing is not applicable to this article as no new data were created or analyzed in this study.
