# Supplementary material for: Cancer risks related to intellectual disabilities: A systematic review
Source: Cancer Med. 2024 Apr 30;13(9):e7210. doi: 10.1002/cam4.7210 (PMC11058689; doi:10.1002/cam4.7210)
Supplement: Supplementary file 2 — Appendix S2: xxx. [file CAM4-13-e7210-s002.docx]

| **Search** | **String (Pubmed)** |
| --- | --- |
| **ID population** | Developmental Disabilities[Mesh] OR "Intellectual Disability"[Mesh] OR "Learning Disabilities"[Mesh] OR "Persons with Mental Disabilities"[Mesh] OR Development Deviation*[tiab] OR Development Disorder*[tiab] OR Developmental Academic Disabilit*[tiab] OR Developmental Academic Disorder*[tiab] OR Developmental Delay Disorder*[tiab] OR Developmental Disabilit*[tiab] OR Developmental Disorders of Scholastic Skills[tiab] OR Idiocy[tiab] OR Intellectual Development Disorder*[tiab] OR Intellectual Disabilit*[tiab] OR Learning Disabilit*[tiab] OR Learning Disturbance*[tiab] OR Mental Deficienc*[tiab] OR Mental Retardation[tiab] |
| **Cancer** | Neoplasms[Majr] OR "Early Detection of Cancer"[Mesh] OR "National Cancer Institute (U.S.)"[Mesh] OR "American Cancer Society"[Mesh] OR "National Program of Cancer Registries"[Mesh] OR Neoplasia*[ti] OR Neoplasm*[ti] OR Tumor*[ti] OR Tumour*[ti] OR Cancer*[ti] OR Malignanc*[ti] OR Neoplasm*[ti] OR Neoplasia*[ot] OR Neoplasm*[ot] OR Tumor*[ot] OR Tumour*[ot] OR Cancer*[ot] OR Malignanc*[ot] OR Neoplasm*[ot] |
| **Incidence** | "Epidemiologic Studies"[Mesh] OR "Case Reports" [Publication Type] OR "Incidence"[Mesh] OR "Epidemiologic Study Characteristics"[Mesh] OR "Epidemiologic Stud*"[tiab] OR case Report* [tiab] OR case stud*[tiab] OR Incidence*[tiab] OR "prevalence" [MeSH] OR prevalence* [tiab] |
| **ID syndromes** |  |
| **Chr + A** | “10q22.3q23.3 Microdeletion Syndrome”[tiab] OR “10q22.3q23.3 Microduplication Syndrome”[tiab] OR “11p15.4 Microduplication Syndrome”[tiab] OR “11q22.2q22.3 Microdeletion Syndrome”[tiab] OR “12q14 Microdeletion Syndrome”[tiab] OR “13q12.3 Microdeletion Syndrome”[tiab] OR “14q11.2 Microdeletion Syndrome”[tiab] OR “14q24.1q24.3 Microdeletion Syndrome”[tiab] OR “15q11.2 Microdeletion Syndrome”[tiab] OR “15q11q13 Microduplication Syndrome”[tiab] OR “15q13.3 Microdeletion Syndrome”[tiab] OR “15q14 Microdeletion Syndrome”[tiab] OR “15q24 Microdeletion Syndrome”[tiab] OR “15q24 Recurrent Microdeletion Syndrome”[tiab] OR “15q26 Overgrowth Syndrome”[tiab] OR “16p11.2-p12.2 Microdeletion Syndrome”[tiab] OR “16p11.2p12.2 Microdeletion Syndrome”[tiab] OR “16p11.2p12.2 Microduplication Syndrome”[tiab] OR “16p13.11 Microdeletion Syndrome”[tiab] OR “16p13.11 Microduplication Syndrome”[tiab] OR “16p13.2 Microdeletion Syndrome”[tiab] OR “16q24.3 Microdeletion Syndrome”[tiab] OR “17p11.2 Microduplication Syndrome”[tiab] OR “17q11 Microdeletion Syndrome”[tiab] OR “17q11.2 Microduplication Syndrome”[tiab] OR “17q12 Microdeletion Syndrome”[tiab] OR “17q12 Microduplication Syndrome”[tiab] OR “17q21.31 Microdeletion Syndrome”[tiab] OR “17q21.31 Microduplication Syndrome”[tiab] OR “17q21.31 Recurrent Microdeletion Syndrome”[tiab] OR “17q24.2 Microdeletion Syndrome”[tiab] OR “19p13.13 Microdeletion Syndrome”[tiab] OR “19p13.3 Microduplication Syndrome”[tiab] OR “19q13.11 Microdeletion Syndrome”[tiab] OR “1p21.3 Microdeletion Syndrome”[tiab] OR “1p36 Deletion Syndrome”[tiab] OR “1p36 Microdeletion Syndrome”[tiab] OR “1q21.1 Microdeletion Syndrome”[tiab] OR “1q21.1 Microduplication Syndrome”[tiab] OR “1q21.1 Recurrent Microdeletion (susceptibility Locus For Neurodevelopmental Disorders)”[tiab] OR “1q21.1 Recurrent Microduplication (possible Susceptibility Locus For Neurodevelopmental Disorders)”[tiab] OR “1q41q42 Microdeletion Syndrome”[tiab] OR “1q44 Microdeletion Syndrome”[tiab] OR “20p13 Microdeletion Syndrome”[tiab] OR “20q11.2 Microduplication Syndrome”[tiab] OR “20q13.33 Microdeletion Syndrome”[tiab] OR “21q22.11q22.12 Microdeletion Syndrome”[tiab] OR “22q11 Duplication Syndrome”[tiab] OR “22q11.2 Deletion Syndrome”[tiab] OR “22q11.2 Distal Deletion Syndrome”[tiab] OR “22q11.2 Duplication Syndrome”[tiab] OR “22q13 Deletion Syndrome (phelan-mcdermid Syndrome)”[tiab] OR “2p15-16.1 Microdeletion Syndrome”[tiab] OR “2p15p16.1 Microdeletion Syndrome”[tiab] OR “2p21 Microdeletion Syndrome”[tiab] OR “2q23.1 Microdeletion Syndrome”[tiab] OR “2q24 Microdeletion Syndrome”[tiab] OR “2q31.1 Microdeletion Syndrome”[tiab] OR “2q32q33 Microdeletion Syndrome”[tiab] OR “2q33.1 Deletion Syndrome”[tiab] OR “2q37 Microdeletion Syndrome”[tiab] OR “2q37 Monosomy”[tiab] OR “3-@methylglutaconic Aciduria, Type V”[tiab] OR “3-hydroxy-3-methylglutaric Aciduria”[tiab] OR “3-methylcrotonyl-coa Carboxylase 1 Deficiency”[tiab] OR “3-methylcrotonyl-coa Carboxylase 2 Deficiency”[tiab] OR “3-methylglutaconic Aciduria Type 3”[tiab] OR “3-methylglutaconic Aciduria Type 4”[tiab] OR “3-methylglutaconic Aciduria Type 7”[tiab] OR “3-methylglutaconic Aciduria Type 9”[tiab] OR “3-methylglutaconic Aciduria With Cataracts, Neurologic Involvement, And Neutropenia”[tiab] OR “3-methylglutaconic Aciduria With Deafness, Encephalopathy, And Leigh-like Syndrome”[tiab] OR “3-methylglutaconic Aciduria, Type Ix”[tiab] OR “3-phosphoserine Phosphatase Deficiency, Infantile/juvenile Form”[tiab] OR “3c Syndrome”[tiab] OR “3mc Syndrome”[tiab] OR “3mc Syndrome 1”[tiab] OR “3mc Syndrome 2”[tiab] OR “3mc Syndrome 3”[tiab] OR “3q27.3 Microdeletion Syndrome”[tiab] OR “3q29 Microdeletion Syndrome”[tiab] OR “3q29 Microduplication Syndrome”[tiab] OR “45,x/46,xy Mixed Gonadal Dysgenesis”[tiab] OR “47,xyy Syndrome”[tiab] OR “48,xxxy Syndrome”[tiab] OR “48,xxyy Syndrome”[tiab] OR “48,xyyy Syndrome”[tiab] OR “49,xxxxy Syndrome”[tiab] OR “49,xxxyy Syndrome”[tiab] OR “49,xyyyy Syndrome”[tiab] OR “4q21 Microdeletion Syndrome”[tiab] OR “5q14.3 Microdeletion Syndrome”[tiab] OR “5q35 Microduplication Syndrome”[tiab] OR “6-pyruvoyl-tetrahydropterin Synthase Deficiency”[tiab] OR “6p22 Microdeletion Syndrome”[tiab] OR “6q Terminal Deletion Syndrome”[tiab] OR “6q25 Microdeletion Syndrome”[tiab] OR “7q11.23 Duplication Syndrome”[tiab] OR “7q11.23 Microduplication Syndrome”[tiab] OR “8p Inverted Duplication/deletion Syndrome”[tiab] OR “8p11.2 Deletion Syndrome”[tiab] OR “8p23.1 Deletion Syndrome”[tiab] OR “8p23.1 Duplication Syndrome”[tiab] OR “8p23.1 Microdeletion Syndrome”[tiab] OR “8q21.11 Microdeletion Syndrome”[tiab] OR “8q24.3 Microdeletion Syndrome”[tiab] OR “9q Subtelomeric Deletion Syndrome”[tiab] OR “9q31.1q31.3 Microdeletion Syndrome”[tiab] OR “9q33.3q34.11 Microdeletion Syndrome”[tiab] OR “Aarskog-scott Syndrome”[tiab] OR “Abetal34v Amyloidosis”[tiab] OR “Absent Eyebrows And Eyelashes With Mental Retardation”[tiab] OR “Achalasia-addisonianism-alacrima Syndrome”[tiab] OR “Achalasia-microcephaly Syndrome”[tiab] OR “Achondroplasia, Severe, With Developmental Delay And Acanthosis Nigricans”[tiab] OR “Acquired Partial Lipodystrophy”[tiab] OR “Acrocallosal Syndrome”[tiab] OR “Acrocardiofacial Syndrome”[tiab] OR “Acrodysostosis”[tiab] OR “Acrodysostosis 1, With Or Without Hormone Resistance”[tiab] OR “Acrodysostosis 2 With Or Without Hormone Resistance”[tiab] OR “Acrodysostosis With Multiple Hormone Resistance”[tiab] OR “Acrofacial Dysostosis, Catania Type”[tiab] OR “Acrofrontofacionasal Dysostosis”[tiab] OR “Acrofrontofacionasal Dysostosis 1”[tiab] OR “Acrogeria”[tiab] OR “Acromegaloid Facial Appearance Syndrome”[tiab] OR “Acromelic Frontonasal Dysostosis”[tiab] OR “Acromelic Frontonasal Dysplasia”[tiab] OR “Acropectoral Syndrome”[tiab] OR “Acropectorovertebral Dysplasia”[tiab] OR “Acute Infantile Liver Failure-cerebellar Ataxia-peripheral Sensory Motor Neuropathy Syndrome”[tiab] OR “Adams-oliver Syndrome”[tiab] OR “Adams-oliver Syndrome 1”[tiab] OR “Adenylosuccinase Deficiency”[tiab] OR “Adenylosuccinate Lyase Deficiency”[tiab] OR “Adult Polyglucosan Body Disease”[tiab] OR “Adult-onset Autosomal Recessive Cerebellar Ataxia”[tiab] OR “Agenesis Of Cerebral White Matter”[tiab] OR “Agenesis Of Corpus Callosum, Cardiac, Ocular, And Genital Syndrome”[tiab] OR “Agenesis Of The Corpus Callosum With Peripheral Neuropathy”[tiab] OR “Ahdc1-related Intellectual Disability-obstructive Sleep Apnea-mild Dysmorphism Syndrome”[tiab] OR “Aica-ribosiduria”[tiab] OR “Aicar Transformylase/imp Cyclohydrolase Deficiency”[tiab] OR “Aicardi Syndrome”[tiab] OR “Aicardi-goutières Syndrome”[tiab] OR “Aicardi-goutieres Syndrome 1”[tiab] OR “Aicardi-goutieres Syndrome 7”[tiab] OR “Al Kaissi Syndrome”[tiab] OR “Alacrima, Achalasia, And Mental Retardation Syndrome”[tiab] OR “Alacrimia-choreoathetosis-liver Dysfunction Syndrome”[tiab] OR “Alagille Syndrome”[tiab] OR “Alagille Syndrome 1”[tiab] OR “Alazami Syndrome”[tiab] OR “Alazami-yuan Syndrome”[tiab] OR “Aldh18a1-related De Barsy Syndrome”[tiab] OR “Alexander Disease”[tiab] OR “Alg1-cdg”[tiab] OR “Alg11-cdg”[tiab] OR “Alg12-cdg”[tiab] OR “Alg2-cdg”[tiab] OR “Alg9-cdg”[tiab] OR “Alkuraya-kucinskas Syndrome”[tiab] OR “Allan-herndon-dudley Syndrome”[tiab] OR “Alobar Holoprosencephaly”[tiab] OR “Alopecia, Neurologic Defects, And Endocrinopathy Syndrome”[tiab] OR “Alopecia, Psychomotor Epilepsy, Pyorrhea, And Mental Subnormality”[tiab] OR “Alopecia-contractures-dwarfism Mental Retardation Syndrome”[tiab] OR “Alopecia-contractures-dwarfism-intellectual Disability Syndrome”[tiab] OR “Alopecia-epilepsy-oligophrenia Syndrome Of Moynahan”[tiab] OR “Alopecia-epilepsy-pyorrhea-intellectual Disability Syndrome”[tiab] OR “Alopecia-intellectual Disability Syndrome”[tiab] OR “Alopecia-intellectual Disability-hypergonadotropic Hypogonadism Syndrome”[tiab] OR “Alopecia-mental Retardation Syndrome 1”[tiab] OR “Alopecia-mental Retardation Syndrome 2”[tiab] OR “Alopecia-mental Retardation Syndrome 3”[tiab] OR “Alopecia-mental Retardation Syndrome 4”[tiab] OR “Alopecia-mental Retardation Syndrome With Convulsions And Hypergonadotropichypogonadism”[tiab] OR “Alpha-dystroglycan-related Limb-girdle Muscular Dystrophy R16”[tiab] OR “Alpha-mannosidosis”[tiab] OR “Alpha-mannosidosis, Adult Form”[tiab] OR “Alpha-mannosidosis, Infantile Form”[tiab] OR “Alpha-methylacetoacetic Aciduria”[tiab] OR “Alpha-n-acetylgalactosaminidase Deficiency”[tiab] OR “Alpha-n-acetylgalactosaminidase Deficiency Type 1”[tiab] OR “Alpha-n-acetylgalactosaminidase Deficiency Type 2”[tiab] OR “Alpha-n-acetylgalactosaminidase Deficiency Type 3”[tiab] OR “Alpha-thalassemia-intellectual Disability Syndrome Linked To Chromosome 16”[tiab] OR “Alpha-thalassemia-x-linked Intellectual Disability Syndrome”[tiab] OR “Alpha-thalassemia/mental Retardation Syndrome, Chromosome 16-related”[tiab] OR “Alpha-thalassemia/mental Retardation Syndrome, X-linked”[tiab] OR “Alport Syndrome - Intellectual Disability - Midface Hypoplasia - Elliptocytosis”[tiab] OR “Alport Syndrome-intellectual Disability-midface Hypoplasia-elliptocytosis Syndrome”[tiab] OR “Alternating Hemiplegia Of Childhood”[tiab] OR “Alternating Hemiplegia Of Childhood 1”[tiab] OR “Alternating Hemiplegia Of Childhood 2”[tiab] OR “Amed Syndrome, Digenic”[tiab] OR “Amelocerebrohypohidrotic Syndrome”[tiab] OR “Amino Aciduria With Mental Deficiency, Dwarfism, Muscular Dystrophy,osteoporosis, And Acidosis”[tiab] OR “Amyloidosis Of Gingiva And Conjunctiva, With Mental Retardation”[tiab] OR “Amyotrophic Dystonic Paraplegia”[tiab] OR “Anauxetic Dysplasia”[tiab] OR “Ane Syndrome”[tiab] OR “Angelman Syndrome”[tiab] OR “Angelman Syndrome (type 1)”[tiab] OR “Angelman Syndrome (type 2)”[tiab] OR “Angelman Syndrome Due To A Point Mutation”[tiab] OR “Angelman Syndrome Due To Maternal 15q11q13 Deletion”[tiab] OR “Angelman Syndrome Due To Paternal Uniparental Disomy Of Chromosome 15”[tiab] OR “Aniridia, Partial, With Unilateral Renal Agenesis And Psychomotorretardation”[tiab] OR “Aniridia-cerebellar Ataxia-intellectual Disability Syndrome”[tiab] OR “Aniridia-intellectual Disability Syndrome”[tiab] OR “Aniridia-ptosis-intellectual Disability-familial Obesity Syndrome”[tiab] OR “Ank3-related Intellectual Disability-sleep Disturbance Syndrome”[tiab] OR “Anophthalmia/microphthalmia-esophageal Atresia Syndrome”[tiab] OR “Antley-bixler Syndrome Without Genital Anomalies Or Disordered Steroidogenesis”[tiab] OR “Aortic Arch Anomaly With Peculiar Facies And Mental Retardation”[tiab] OR “Aortic Arch Anomaly-facial Dysmorphism-intellectual Disability Syndrome”[tiab] OR “Apert Syndrome”[tiab] OR “Aphalangia, Partial, With Syndactyly And Duplication Of Metatarsaliv”[tiab] OR “Aphonia-deafness-retinal Dystrophy-bifid Halluces-intellectual Disability Syndrome”[tiab] OR “Arachnodactyly-abnormal Ossification-intellectual Disability Syndrome”[tiab] OR “Arachnoid Cyst”[tiab] OR “Arachnoid Cysts, Intracranial”[tiab] OR “Arboleda-tham Syndrome”[tiab] OR “Argininemia”[tiab] OR “Argininosuccinic Aciduria”[tiab] OR “Arima Syndrome”[tiab] OR “Arterial Tortuosity Syndrome”[tiab] OR “Arthrogryposis Multiplex Congenita 5”[tiab] OR “Arthrogryposis, Cleft Palate, Craniosynostosis, And Impaired Intellectual Development”[tiab] OR “Arthrogryposis, Distal, Type 2a”[tiab] OR “Arthrogryposis, Distal, Type 3”[tiab] OR “Arthrogryposis, Distal, Type 4”[tiab] OR “Arthrogryposis, Distal, With Hypopituitarism, Mental Retardation,and Facial Anomalies”[tiab] OR “Arthrogryposis, Distal, With Mental Retardation And Characteristicfacies”[tiab] OR “Arthrogryposis, Mental Retardation, And Seizures”[tiab] OR “Arts Syndrome”[tiab] OR “Aspartylglucosaminuria”[tiab] OR “Ataxia, Deafness, And Cardiomyopathy”[tiab] OR “Ataxia, Posterior Column, With Retinitis Pigmentosa”[tiab] OR “Ataxia, Spastic, Childhood-onset, Autosomal Recessive, With Optic Atrophy And Mental Retardation”[tiab] OR “Ataxia-deafness-intellectual Disability Syndrome”[tiab] OR “Ataxia-deafness-retardation Syndrome”[tiab] OR “Ataxia-intellectual Disability-oculomotor Apraxia-cerebellar Cysts Syndrome”[tiab] OR “Ataxia-microcephaly-cataract Syndrome”[tiab] OR “Athyreosis”[tiab] OR “Atkin-flaitz Syndrome”[tiab] OR “Atr-16 Syndrome”[tiab] OR “Attenuated Chédiak-higashi Syndrome”[tiab] OR “Atypical Juvenile Parkinsonism”[tiab] OR “Atypical Rett Syndrome”[tiab] OR “Au-kline Syndrome”[tiab] OR “Aural Atresia, Multiple Congenital Anomalies, And Mental Retardation”[tiab] OR “Aurocephalosyndactyly”[tiab] OR “Autism Spectrum Disorder Due To Auts2 Deficiency”[tiab] OR “Autism Spectrum Disorder-epilepsy-arthrogryposis Syndrome”[tiab] OR “Autism Susceptibility 1”[tiab] OR “Autism Susceptibility, X-linked 1”[tiab] OR “Autism Susceptibility, X-linked 3”[tiab] OR “Autism, Susceptibility To, 18”[tiab] OR “Autism, Susceptibility To, 3”[tiab] OR “Autism, Susceptibility To, 8”[tiab] OR “Autism, Susceptibility To, X-linked 2”[tiab] OR “Autism, Susceptibility To, X-linked 4”[tiab] OR “Autism, Susceptibility To, X-linked 6”[tiab] OR “Autosomal Dominant Cerebellar Ataxia”[tiab] OR “Autosomal Dominant Charcot-marie-tooth Disease Type 2z”[tiab] OR “Autosomal Dominant Deafness-onychodystrophy Syndrome”[tiab] OR “Autosomal Dominant Intellectual Disability-craniofacial Anomalies-cardiac Defects Syndrome”[tiab] OR “Autosomal Dominant Nocturnal Frontal Lobe Epilepsy”[tiab] OR “Autosomal Dominant Non-syndromic Intellectual Disability”[tiab] OR “Autosomal Dominant Robinow Syndrome”[tiab] OR “Autosomal Dominant Spastic Paraplegia Type 10”[tiab] OR “Autosomal Dominant Spastic Paraplegia Type 4”[tiab] OR “Autosomal Recessive Ataxia Due To Pex10 Deficiency”[tiab] OR “Autosomal Recessive Ataxia Due To Ubiquinone Deficiency”[tiab] OR “Autosomal Recessive Ataxia, Beauce Type”[tiab] OR “Autosomal Recessive Axonal Neuropathy With Neuromyotonia”[tiab] OR “Autosomal Recessive Centronuclear Myopathy”[tiab] OR “Autosomal Recessive Cerebellar Ataxia Due To Cwf19l1 Deficiency”[tiab] OR “Autosomal Recessive Cerebellar Ataxia With Late-onset Spasticity”[tiab] OR “Autosomal Recessive Cerebellar Ataxia-epilepsy-intellectual Disability Syndrome Due To Rubcn Deficiency”[tiab] OR “Autosomal Recessive Cerebellar Ataxia-epilepsy-intellectual Disability Syndrome Due To Tud Deficiency”[tiab] OR “Autosomal Recessive Cerebellar Ataxia-psychomotor Delay Syndrome”[tiab] OR “Autosomal Recessive Cerebellar Ataxia-pyramidal Signs-nystagmus-oculomotor Apraxia Syndrome”[tiab] OR “Autosomal Recessive Cerebelloparenchymal Disorder Type 3”[tiab] OR “Autosomal Recessive Chorioretinopathy-microcephaly Syndrome”[tiab] OR “Autosomal Recessive Complex Spastic Paraplegia Due To Kennedy Pathway Dysfunction”[tiab] OR “Autosomal Recessive Congenital Cerebellar Ataxia Due To Mglur1 Deficiency”[tiab] OR “Autosomal Recessive Cutis Laxa Type 1”[tiab] OR “Autosomal Recessive Cutis Laxa Type 2, Classic Type”[tiab] OR “Autosomal Recessive Cutis Laxa Type 2a”[tiab] OR “Autosomal Recessive Distal Osteolysis Syndrome”[tiab] OR “Autosomal Recessive Dopa-responsive Dystonia”[tiab] OR “Autosomal Recessive Hyperinsulinism Due To Kir6.2 Deficiency”[tiab] OR “Autosomal Recessive Non-syndromic Intellectual Disability”[tiab] OR “Autosomal Recessive Omodysplasia”[tiab] OR “Autosomal Recessive Primary Microcephaly”[tiab] OR “Autosomal Recessive Robinow Syndrome”[tiab] OR “Autosomal Recessive Spastic Ataxia With Leukoencephalopathy”[tiab] OR “Autosomal Recessive Spastic Paraplegia Type 11”[tiab] OR “Autosomal Recessive Spastic Paraplegia Type 14”[tiab] OR “Autosomal Recessive Spastic Paraplegia Type 15”[tiab] OR “Autosomal Recessive Spastic Paraplegia Type 18”[tiab] OR “Autosomal Recessive Spastic Paraplegia Type 26”[tiab] OR “Autosomal Recessive Spastic Paraplegia Type 32”[tiab] OR “Autosomal Recessive Spastic Paraplegia Type 35”[tiab] OR “Autosomal Recessive Spastic Paraplegia Type 45”[tiab] OR “Autosomal Recessive Spastic Paraplegia Type 48”[tiab] OR “Autosomal Recessive Spastic Paraplegia Type 54”[tiab] OR “Autosomal Recessive Spastic Paraplegia Type 55”[tiab] OR “Autosomal Recessive Spastic Paraplegia Type 56”[tiab] OR “Autosomal Recessive Spastic Paraplegia Type 59”[tiab] OR “Autosomal Recessive Spastic Paraplegia Type 60”[tiab] OR “Autosomal Recessive Spastic Paraplegia Type 64”[tiab] OR “Autosomal Recessive Spastic Paraplegia Type 66”[tiab] OR “Autosomal Recessive Spastic Paraplegia Type 67”[tiab] OR “Autosomal Recessive Spastic Paraplegia Type 69”[tiab] OR “Autosomal Recessive Spastic Paraplegia Type 70”[tiab] OR “Autosomal Recessive Spastic Paraplegia Type 71”[tiab] OR “Autosomal Recessive Spastic Paraplegia Type 75”[tiab] OR “Autosomal Recessive Spondylocostal Dysostosis”[tiab] OR “Autosomal Spastic Paraplegia Type 58”[tiab] OR “Axenfeld-rieger Anomaly With Partially Absent Eye Muscles, Distinctive Face, Hydrocephaly, And Skeletal Abnormalities”[tiab] OR “Aymé-gripp Syndrome”[tiab] OR “Ayme-gripp Syndrome”[tiab] |
| **B, C, D** | “Bainbridge-ropers Syndrome”[tiab] OR “Baller-gerold Syndrome”[tiab] OR “Bamforth-lazarus Syndrome”[tiab] OR “Band Heterotopia”[tiab] OR “Band-like Calcification With Simplified Gyration And Polymicrogyria”[tiab] OR “Bangstad Syndrome”[tiab] OR “Bannayan-riley-ruvalcaba Syndrome”[tiab] OR “Baraitser-winter Cerebrofrontofacial Syndrome”[tiab] OR “Baraitser-winter Syndrome 1”[tiab] OR “Baraitser-winter Syndrome 2”[tiab] OR “Baralle-macken Syndrome”[tiab] OR “Barber-say Syndrome”[tiab] OR “Bardet-biedl Syndrome”[tiab] OR “Bardet-biedl Syndrome 1”[tiab] OR “Bardet-biedl Syndrome 13”[tiab] OR “Bardet-biedl Syndrome 14”[tiab] OR “Bardet-biedl Syndrome 16”[tiab] OR “Bardet-biedl Syndrome 17”[tiab] OR “Bardet-biedl Syndrome 19”[tiab] OR “Bardet-biedl Syndrome 2”[tiab] OR “Bardet-biedl Syndrome 20”[tiab] OR “Bardet-biedl Syndrome 22”[tiab] OR “Bardet-biedl Syndrome 3”[tiab] OR “Bardet-biedl Syndrome 4”[tiab] OR “Bardet-biedl Syndrome 6”[tiab] OR “Bardet-biedl Syndrome 7”[tiab] OR “Bardet-biedl Syndrome 8”[tiab] OR “Bardet-biedl Syndrome 9”[tiab] OR “Bartsocas-papas Syndrome”[tiab] OR “Bartter Syndrome, Antenatal, Type 2”[tiab] OR “Bartter Syndrome, Type 1, Antenatal”[tiab] OR “Bartter Syndrome, Type 4a, Neonatal, With Sensorineural Deafness”[tiab] OR “Bartter Syndrome, Type 4b, Neonatal, With Sensorineural Deafness”[tiab] OR “Basal Cell Nevus Syndrome”[tiab] OR “Basal Ganglia Calcification, Idiopathic, Childhood-onset”[tiab] OR “Basel-vanagaite-smirin-yosef Syndrome”[tiab] OR “Basilicata-akhtar Syndrome”[tiab] OR “Beaulieu-boycott-innes Syndrome”[tiab] OR “Beck-fahrner Syndrome”[tiab] OR “Behr Syndrome”[tiab] OR “Benign Adult Familial Myoclonic Epilepsy”[tiab] OR “Beta-ketothiolase Deficiency”[tiab] OR “Beta-mannosidosis”[tiab] OR “Beta-mercaptolactate Cysteine Disulfiduria”[tiab] OR “Beta-propeller Protein-associated Neurodegeneration”[tiab] OR “Beta-ureidopropionase Deficiency”[tiab] OR “Biemond Syndrome Ii”[tiab] OR “Biemond Syndrome Type 2”[tiab] OR “Bifid Nose With Or Without Anorectal And Renal Anomalies”[tiab] OR “Bilateral Frontal Polymicrogyria”[tiab] OR “Bilateral Frontoparietal Polymicrogyria”[tiab] OR “Bilateral Generalized Polymicrogyria”[tiab] OR “Bilateral Parasagittal Parieto-occipital Polymicrogyria”[tiab] OR “Bilateral Perisylvian Polymicrogyria”[tiab] OR “Bilateral Polymicrogyria”[tiab] OR “Bile Acid Synthesis Defect, Congenital, 6”[tiab] OR “Biotinidase Deficiency”[tiab] OR “Birk-barel Mental Retardation Dysmorphism Syndrome”[tiab] OR “Björnstad Syndrome”[tiab] OR “Bjornstad Syndrome”[tiab] OR “Blepharonasofacial Malformation Syndrome”[tiab] OR “Blepharophimosis With Facial And Genital Anomalies And Mental Retardation”[tiab] OR “Blepharophimosis With Ptosis, Syndactyly, And Short Stature”[tiab] OR “Blepharophimosis-impaired Intellectual Development Syndrome”[tiab] OR “Blepharophimosis-intellectual Disability Syndrome, Mkb Type”[tiab] OR “Blepharophimosis-intellectual Disability Syndrome, Ohdo Type”[tiab] OR “Blepharophimosis-intellectual Disability Syndrome, Sbbys Type”[tiab] OR “Blepharophimosis-intellectual Disability Syndrome, Verloes Type”[tiab] OR “Blepharophimosis-ptosis-esotropia-syndactyly-short Stature Syndrome”[tiab] OR “Bloom Syndrome”[tiab] OR “Body Mass Index Quantitative Trait Locus 19”[tiab] OR “Bohring-opitz Syndrome”[tiab] OR “Bonnemann-meinecke-reich Syndrome”[tiab] OR “Borjeson-forssman-lehmann Syndrome”[tiab] OR “Bosch-boonstra-schaaf Optic Atrophy Syndrome”[tiab] OR “Bosma Arhinia Microphthalmia Syndrome”[tiab] OR “Boucher-neuhauser Syndrome”[tiab] OR “Brachycephaly, Deafness, Cataract, Microstomia, And Mental Retardation”[tiab] OR “Brachydactyly, Type A1, With Short Stature, Scoliosis, Microcephaly,ptosis, Hearing Loss, And Mental Retardation”[tiab] OR “Brachydactyly-nystagmus-cerebellar Ataxia”[tiab] OR “Brachydactyly-short Stature-retinitis Pigmentosa Syndrome”[tiab] OR “Brachymorphism-onychodysplasia-dysphalangism Syndrome”[tiab] OR “Brain Malformations With Or Without Urinary Tract Defects”[tiab] OR “Brain Malformations-musculoskeletal Abnormalities-facial Dysmorphism-intellectual Disability Syndrome”[tiab] OR “Brain Small Vessel Disease 1 With Or Without Ocular Anomalies”[tiab] OR “Brain-lung-thyroid Syndrome”[tiab] OR “Branched-chain Ketoacid Dehydrogenase Kinase Deficiency”[tiab] OR “Branchiooculofacial Syndrome”[tiab] OR “Branchioskeletogenital Syndrome”[tiab] OR “Bresek Syndrome”[tiab] OR “Brooks-wisniewski-brown Syndrome”[tiab] OR “Brunner Syndrome”[tiab] OR “Bullous Dystrophy, Hereditary Macular Type”[tiab] OR “C Syndrome”[tiab] OR “Cahmr Syndrome”[tiab] OR “Camos Syndrome”[tiab] OR “Camptodactyly Syndrome, Guadalajara Type 1”[tiab] OR “Camptodactyly Syndrome, Guadalajara Type 3”[tiab] OR “Camptodactyly Syndrome, Guadalajara, Type I”[tiab] OR “Camptodactyly Syndrome, Guadalajara, Type Iii”[tiab] OR “Camptodactyly, Tall Stature, And Hearing Loss Syndrome”[tiab] OR “Cantú Syndrome”[tiab] OR “Carbamoyl Phosphate Synthetase I Deficiency, Hyperammonemia Due To”[tiab] OR “Cardiac-urogenital Syndrome”[tiab] OR “Cardiac-valvular Ehlers-danlos Syndrome”[tiab] OR “Cardiocranial Syndrome, Pfeiffer Type”[tiab] OR “Cardiofaciocutaneous Syndrome”[tiab] OR “Cardiofaciocutaneous Syndrome 1”[tiab] OR “Cardiofaciocutaneous Syndrome 3”[tiab] OR “Carey-fineman-ziter Syndrome”[tiab] OR “Carnosinase Deficiency”[tiab] OR “Carnosinemia”[tiab] OR “Carpenter Syndrome”[tiab] OR “Carpenter Syndrome 1”[tiab] OR “Cat Eye Syndrome”[tiab] OR “Cat-eye Syndrome”[tiab] OR “Cat-eye Syndrome (type I)”[tiab] OR “Cataract 11, Multiple Types”[tiab] OR “Cataract, Ataxia, Short Stature, And Mental Retardation”[tiab] OR “Cataract, Microcephaly, Failure To Thrive, Kyphoscoliosis Syndrome”[tiab] OR “Cataract-ataxia-deafness Syndrome”[tiab] OR “Cataract-ataxia-deafness-retardation Syndrome”[tiab] OR “Cataract-deafness-hypogonadism Syndrome”[tiab] OR “Cataract-hypertrichosis-intellectual Disability Syndrome”[tiab] OR “Cataract-intellectual Disability-anal Atresia-urinary Defects Syndrome”[tiab] OR “Cataract-intellectual Disability-hypogonadism Syndrome”[tiab] OR “Cataract-nephropathy-encephalopathy Syndrome”[tiab] OR “Catifa Syndrome”[tiab] OR “Caudal Appendage-deafness Syndrome”[tiab] OR “Cebalid Syndrome”[tiab] OR “Cednik Syndrome”[tiab] OR “Cephalin Lipidosis”[tiab] OR “Cerebellar Ataxia, Brain Abnormalities, And Cardiac Conduction Defects”[tiab] OR “Cerebellar Ataxia, Mental Retardation, And Dysequilibrium Syndrome 1”[tiab] OR “Cerebellar Ataxia, Mental Retardation, And Dysequilibrium Syndrome2”[tiab] OR “Cerebellar Ataxia, Mental Retardation, And Dysequilibrium Syndrome3”[tiab] OR “Cerebellar Ataxia, Mental Retardation, And Dysequilibrium Syndrome4”[tiab] OR “Cerebellar Atrophy, Visual Impairment, And Psychomotor Retardation”[tiab] OR “Cerebellar Dysfunction With Variable Cognitive And Behavioral Abnormalities”[tiab] OR “Cerebellar Dysfunction, Impaired Intellectual Development, And Hypogonadotropic Hypogonadism”[tiab] OR “Cerebellar-facial-dental Syndrome”[tiab] OR “Cerebellofaciodental Syndrome”[tiab] OR “Cerebral Creatine Deficiency Syndrome 1”[tiab] OR “Cerebral Creatine Deficiency Syndrome 2”[tiab] OR “Cerebral Creatine Deficiency Syndrome 3”[tiab] OR “Cerebral Dysgenesis, Neuropathy, Ichthyosis, And Palmoplantar Keratoderma Syndrome”[tiab] OR “Cerebral Palsy, Spastic Quadriplegic, 2”[tiab] OR “Cerebral Visual Impairment”[tiab] OR “Cerebrocostomandibular Syndrome”[tiab] OR “Cerebrofacioarticular Syndrome”[tiab] OR “Cerebrofaciothoracic Dysplasia”[tiab] OR “Cerebrooculofacioskeletal Syndrome 1”[tiab] OR “Cerebrooculonasal Syndrome”[tiab] OR “Cerebrotendinous Xanthomatosis”[tiab] OR “Ceroid Lipofuscinosis, Neuronal, 1”[tiab] OR “Ceroid Lipofuscinosis, Neuronal, 10”[tiab] OR “Ceroid Lipofuscinosis, Neuronal, 3”[tiab] OR “Ceroid Lipofuscinosis, Neuronal, 5”[tiab] OR “Ceroid Lipofuscinosis, Neuronal, 8, Northern Epilepsy Variant”[tiab] OR “Ceroid Lipofuscinosis, Neuronal, 9”[tiab] OR “Chanarin-dorfman Syndrome”[tiab] OR “Char Syndrome”[tiab] OR “Charcot-marie-tooth Disease, Type 4b3”[tiab] OR “Charcot-marie-tooth Disease, X-linked Recessive, 2”[tiab] OR “Charcot-marie-tooth Disease-deafness-intellectual Disability Syndrome”[tiab] OR “Charge Syndrome”[tiab] OR “Chediak-higashi Syndrome”[tiab] OR “Chédiak-higashi Syndrome”[tiab] OR “Childhood Absence Epilepsy”[tiab] OR “Childhood Disintegrative Disorder”[tiab] OR “Childhood-onset Motor And Cognitive Regression Syndrome With Extrapyramidal Movement Disorder”[tiab] OR “Chime Syndrome”[tiab] OR “Chondrodysplasia Punctata 2, X-linked Dominant”[tiab] OR “Chondrodysplasia With Platyspondyly, Distinctive Brachydactyly, Hydrocephaly, And Microphthalmia”[tiab] OR “Chondrodysplasia-disorder Of Sex Development Syndrome”[tiab] OR “Chops Syndrome”[tiab] OR “Chorea, Childhood-onset, With Psychomotor Retardation”[tiab] OR “Choroid Plexus Calcification And Mental Retardation”[tiab] OR “Christianson Syndrome”[tiab] OR “Chromosome 10q26 Deletion Syndrome”[tiab] OR “Chromosome 11p13 Deletion Syndrome, Distal”[tiab] OR “Chromosome 13q14 Deletion Syndrome”[tiab] OR “Chromosome 13q33-q34 Deletion Syndrome”[tiab] OR “Chromosome 14q11-q22 Deletion Syndrome”[tiab] OR “Chromosome 15q11-q13 Duplication Syndrome”[tiab] OR “Chromosome 15q11.2 Deletion Syndrome”[tiab] OR “Chromosome 15q13.3 Microdeletion Syndrome”[tiab] OR “Chromosome 15q14 Deletion Syndrome”[tiab] OR “Chromosome 15q26-qter Deletion Syndrome”[tiab] OR “Chromosome 16p12.2-p11.2 Deletion Syndrome, 7.1- To 8.7-mb”[tiab] OR “Chromosome 16p13.2 Deletion Syndrome”[tiab] OR “Chromosome 16p13.3 Deletion Syndrome”[tiab] OR “Chromosome 16p13.3 Duplication Syndrome”[tiab] OR “Chromosome 17q11.2 Deletion Syndrome, 1.4-mb”[tiab] OR “Chromosome 17q12 Deletion Syndrome”[tiab] OR “Chromosome 17q12 Duplication Syndrome”[tiab] OR “Chromosome 17q23.1-q23.2 Deletion Syndrome”[tiab] OR “Chromosome 18p Deletion Syndrome”[tiab] OR “Chromosome 18q Deletion Syndrome”[tiab] OR “Chromosome 19p13.13 Deletion Syndrome”[tiab] OR “Chromosome 19q13.11 Deletion Syndrome, Distal”[tiab] OR “Chromosome 19q13.11 Deletion Syndrome, Proximal”[tiab] OR “Chromosome 1p35 Deletion Syndrome”[tiab] OR “Chromosome 1p36 Deletion Syndrome”[tiab] OR “Chromosome 1q21.1 Deletion Syndrome, 1.35-mb”[tiab] OR “Chromosome 1q21.1 Duplication Syndrome”[tiab] OR “Chromosome 1q41-q42 Deletion Syndrome”[tiab] OR “Chromosome 20q11-q12 Deletion Syndrome”[tiab] OR “Chromosome 22q11.2 Duplication Syndrome”[tiab] OR “Chromosome 2p16.1-p15 Deletion Syndrome”[tiab] OR “Chromosome 2q37 Deletion Syndrome”[tiab] OR “Chromosome 3q29 Deletion Syndrome”[tiab] OR “Chromosome 3q29 Duplication Syndrome”[tiab] OR “Chromosome 5p13 Duplication Syndrome”[tiab] OR “Chromosome 5q12 Deletion Syndrome”[tiab] OR “Chromosome 6pter-p24 Deletion Syndrome”[tiab] OR “Chromosome 6q25-q25 Deletion Syndrome”[tiab] OR “Chromosome 8q21.11 Deletion Syndrome”[tiab] OR “Chromosome 9p Deletion Syndrome”[tiab] OR “Chromosome Xp11.23-p11.22 Duplication Syndrome”[tiab] OR “Chromosome Xp11.3 Deletion Syndrome”[tiab] OR “Chromosome Xq27.3-q28 Duplication Syndrome”[tiab] OR “Chronic Bilirubin Encephalopathy”[tiab] OR “Chronic Visceral Acid Sphingomyelinase Deficiency”[tiab] OR “Chudley-mccullough Syndrome”[tiab] OR “Chylomicron Retention Disease”[tiab] OR “Cimdag Syndrome”[tiab] OR “Cinca Syndrome”[tiab] OR “Citrullinemia Type I”[tiab] OR “Citrullinemia, Classic”[tiab] OR “Ck Syndrome”[tiab] OR “Clark-baraitser Syndrome”[tiab] OR “Classic Galactosemia”[tiab] OR “Classic Glucose Transporter Type 1 Deficiency Syndrome”[tiab] OR “Classic Homocystinuria”[tiab] OR “Classic Phenylketonuria”[tiab] OR “Clcn4-related X-linked Intellectual Disability Syndrome”[tiab] OR “Cleft Lip/palate With Abnormal Thumbs And Microcephaly”[tiab] OR “Cleft Lip/palate-ectodermal Dysplasia Syndrome”[tiab] OR “Cleft Palate, Cardiac Defect, Genital Anomalies, And Ectrodactyly”[tiab] OR “Cleft Palate, Cardiac Defects, And Mental Retardation”[tiab] OR “Cleft Palate, Isolated”[tiab] OR “Cleft Palate-short Stature-vertebral Anomalies Syndrome”[tiab] OR “Cntnap2-related Developmental And Epileptic Encephalopathy”[tiab] OR “Coach Syndrome 1”[tiab] OR “Coach Syndrome 2”[tiab] OR “Cockayne Syndrome”[tiab] OR “Cockayne Syndrome A”[tiab] OR “Cockayne Syndrome Type 1”[tiab] OR “Cockayne Syndrome Type 2”[tiab] OR “Cockayne Syndrome Type 3”[tiab] OR “Cockayne Syndrome, Type B”[tiab] OR “Codas Syndrome”[tiab] OR “Coenzyme Q10 Deficiency, Primary, 1”[tiab] OR “Coenzyme Q10 Deficiency, Primary, 2”[tiab] OR “Coenzyme Q10 Deficiency, Primary, 4”[tiab] OR “Coffin-lowry Syndrome”[tiab] OR “Coffin-siris Syndrome”[tiab] OR “Coffin-siris Syndrome 1”[tiab] OR “Coffin-siris Syndrome 10”[tiab] OR “Coffin-siris Syndrome 11”[tiab] OR “Coffin-siris Syndrome 12”[tiab] OR “Coffin-siris Syndrome 2”[tiab] OR “Coffin-siris Syndrome 3”[tiab] OR “Coffin-siris Syndrome 4”[tiab] OR “Coffin-siris Syndrome 5”[tiab] OR “Coffin-siris Syndrome 6”[tiab] OR “Coffin-siris Syndrome 7”[tiab] OR “Coffin-siris Syndrome 9”[tiab] OR “Cog1-cdg”[tiab] OR “Cog2-cdg”[tiab] OR “Cog5-cdg”[tiab] OR “Cog8-cdg”[tiab] OR “Cognitive Impairment With Or Without Cerebellar Ataxia”[tiab] OR “Cognitive Impairment-coarse Facies-heart Defects-obesity-pulmonary Involvement-short Stature-skeletal Dysplasia Syndrome”[tiab] OR “Cohen Syndrome”[tiab] OR “Cohen-gibson Syndrome”[tiab] OR “Coloboma, Ocular, With Or Without Hearing Impairment, Cleft Lip/palate, And/or Mental Retardation”[tiab] OR “Coloboma-obesity-hypogenitalism-mental Retardation Syndrome”[tiab] OR “Colobomatous Microphthalmia-obesity-hypogenitalism-intellectual Disability Syndrome”[tiab] OR “Combined Immunodeficiency And Megaloblastic Anemia With Or Without Hyperhomocysteinemia”[tiab] OR “Combined Immunodeficiency With Faciooculoskeletal Anomalies”[tiab] OR “Combined Oxidative Phosphorylation Defect Type 23”[tiab] OR “Combined Oxidative Phosphorylation Defect Type 27”[tiab] OR “Combined Oxidative Phosphorylation Deficiency 18”[tiab] OR “Combined Oxidative Phosphorylation Deficiency 24”[tiab] OR “Combined Oxidative Phosphorylation Deficiency 35”[tiab] OR “Combined Oxidative Phosphorylation Deficiency 36”[tiab] OR “Cone-rod Dystrophy 1”[tiab] OR “Congenital Cataracts, Facial Dysmorphism, And Neuropathy”[tiab] OR “Congenital Cataracts-facial Dysmorphism-neuropathy Syndrome”[tiab] OR “Congenital Disorder Of Glycosylation With Defective Fucosylation 1”[tiab] OR “Congenital Disorder Of Glycosylation, Type 2v”[tiab] OR “Congenital Disorder Of Glycosylation, Type Icc”[tiab] OR “Congenital Disorder Of Glycosylation, Type Ii”[tiab] OR “Congenital Disorder Of Glycosylation, Type Iia”[tiab] OR “Congenital Disorder Of Glycosylation, Type Iic”[tiab] OR “Congenital Disorder Of Glycosylation, Type Iif”[tiab] OR “Congenital Disorder Of Glycosylation, Type Iih”[tiab] OR “Congenital Disorder Of Glycosylation, Type Iii”[tiab] OR “Congenital Disorder Of Glycosylation, Type Iil”[tiab] OR “Congenital Disorder Of Glycosylation, Type Iim”[tiab] OR “Congenital Disorder Of Glycosylation, Type Iin”[tiab] OR “Congenital Disorder Of Glycosylation, Type Iit”[tiab] OR “Congenital Disorder Of Glycosylation, Type Ij”[tiab] OR “Congenital Disorder Of Glycosylation, Type Il”[tiab] OR “Congenital Disorder Of Glycosylation, Type In”[tiab] OR “Congenital Disorder Of Glycosylation, Type Iq”[tiab] OR “Congenital Disorder Of Glycosylation, Type Iw”[tiab] OR “Congenital Disorder Of Glycosylation, Type Iw, Autosomal Dominant”[tiab] OR “Congenital Disorder Of Glycosylation, Type Ix”[tiab] OR “Congenital Disorder Of Glycosylation, Type Iy”[tiab] OR “Congenital Generalized Lipodystrophy”[tiab] OR “Congenital Heart Defects, Dysmorphic Facial Features, And Intellectual Developmental Disorder”[tiab] OR “Congenital Hemidysplasia With Ichthyosiform Erythroderma And Limb Defects”[tiab] OR “Congenital Hydrocephalus”[tiab] OR “Congenital Hypothyroidism”[tiab] OR “Congenital Labioscrotal Agenesis-cerebellar Malformation-corneal Dystrophy-facial Dysmorphism Syndrome”[tiab] OR “Congenital Muscular Dystrophy With Cerebellar Involvement”[tiab] OR “Congenital Muscular Dystrophy With Intellectual Disability”[tiab] OR “Congenital Muscular Dystrophy, Fukuyama Type”[tiab] OR “Congenital Myasthenic Syndrome”[tiab] OR “Congenital Progressive Bone Marrow Failure-b-cell Immunodeficiency-skeletal Dysplasia Syndrome”[tiab] OR “Congenital Rubella Syndrome”[tiab] OR “Congenital Varicella Syndrome”[tiab] OR “Cono-spondylar Dysplasia”[tiab] OR “Continuous Spikes And Waves During Sleep”[tiab] OR “Convulsive Disorder, Familial, With Prenatal Or Early Onset”[tiab] OR “Cooper-jabs Syndrome”[tiab] OR “Corneal Hypesthesia With Retinal Abnormalities, Sensorineural Deafness,unusual Facies, Persistent Ductus Arteriosus, And Mental Retardation”[tiab] OR “Cornelia De Lange Syndrome”[tiab] OR “Cornelia De Lange Syndrome 1”[tiab] OR “Cornelia De Lange Syndrome 2”[tiab] OR “Cornelia De Lange Syndrome 3”[tiab] OR “Cornelia De Lange Syndrome 5”[tiab] OR “Corpus Callosum Agenesis-abnormal Genitalia Syndrome”[tiab] OR “Corpus Callosum Agenesis-intellectual Disability-coloboma-micrognathia Syndrome”[tiab] OR “Corpus Callosum Agenesis-macrocephaly-hypertelorism Syndrome”[tiab] OR “Corpus Callosum Agenesis-neuronopathy Syndrome”[tiab] OR “Corpus Callosum, Agenesis Of”[tiab] OR “Corpus Callosum, Agenesis Of, With Abnormal Genitalia”[tiab] OR “Corpus Callosum, Agenesis Of, With Facial Anomalies And Cerebellar Ataxia”[tiab] OR “Corpus Callosum, Agenesis Of, With Facial Anomalies And Robin Sequence”[tiab] OR “Corpus Callosum, Agenesis Of, With Mental Retardation, Ocular Coloboma,and Micrognathia”[tiab] OR “Corpus Callosum, Partial Agenesis Of, X-linked”[tiab] OR “Cortical Blindness-intellectual Disability-polydactyly Syndrome”[tiab] OR “Cortical Dysgenesis With Pontocerebellar Hypoplasia Due To Tubb3 Mutation”[tiab] OR “Cortical Dysplasia, Complex, With Other Brain Malformations 1”[tiab] OR “Cortical Dysplasia, Complex, With Other Brain Malformations 10”[tiab] OR “Cortical Dysplasia, Complex, With Other Brain Malformations 2”[tiab] OR “Cortical Dysplasia, Complex, With Other Brain Malformations 7”[tiab] OR “Cortical Dysplasia, Complex, With Other Brain Malformations 9”[tiab] OR “Costello Syndrome”[tiab] OR “Cowchock Syndrome”[tiab] OR “Cowden Syndrome”[tiab] OR “Cowden Syndrome 1”[tiab] OR “Cowden Syndrome 5”[tiab] OR “Cowden Syndrome 6”[tiab] OR “Craniodiaphyseal Dysplasia”[tiab] OR “Craniodigital-intellectual Disability Syndrome”[tiab] OR “Craniofacial Dysmorphism, Skeletal Anomalies, And Mental Retardation Syndrome”[tiab] OR “Craniofacial Dyssynostosis With Short Stature”[tiab] OR “Craniofaciofrontodigital Syndrome”[tiab] OR “Craniofrontonasal Dysplasia”[tiab] OR “Craniopharyngioma”[tiab] OR “Craniostenosis, Sagittal, With Congenital Heart Disease, Mental Deficiency, And Mandibular Ankylosis”[tiab] OR “Craniosynostosis-anal Anomalies-porokeratosis Syndrome”[tiab] OR “Craniosynostosis-dandy-walker Malformation-hydrocephalus Syndrome”[tiab] OR “Craniosynostosis-hydrocephalus-arnold-chiari Malformation Type I-radioulnar Synostosis Syndrome”[tiab] OR “Craniosynostosis-mental Retardation Syndrome Of Lin And Gettig”[tiab] OR “Craniosynostosis-mental Retardation-clefting Syndrome”[tiab] OR “Cree Mental Retardation Syndrome”[tiab] OR “Cri Du Chat Syndrome (5p Deletion)”[tiab] OR “Cri-du-chat Syndrome”[tiab] OR “Crigler-najjar Syndrome Type 1”[tiab] OR “Crisponi/cold-induced Sweating Syndrome 1”[tiab] OR “Crome Syndrome”[tiab] OR “Crouzon Syndrome”[tiab] OR “Cryptorchidism-arachnodactyly-intellectual Disability Syndrome”[tiab] OR “Ctcf-related Neurodevelopmental Disorder”[tiab] OR “Cubitus Valgus With Mental Retardation And Unusual Facies”[tiab] OR “Curry-jones Syndrome”[tiab] OR “Cutaneous Mastocytosis, Conductive Hearing Loss, And Microtia”[tiab] OR “Cutis Laxa, Autosomal Recessive, Type Iia”[tiab] OR “Cutis Laxa, Autosomal Recessive, Type Iiia”[tiab] OR “Cutis Laxa, Autosomal Recessive, Type Iiib”[tiab] OR “Cutis Verticis Gyrata And Mental Deficiency”[tiab] OR “Cutis Verticis Gyrata, Thyroid Aplasia, And Mental Retardation”[tiab] OR “Cyclic Vomiting Syndrome”[tiab] OR “Cystathioninuria”[tiab] OR “Cysteine Peptiduria”[tiab] OR “Cystic Fibrosis With Helicobacter Pylori Gastritis, Megaloblastic Anemia, And Mental Retardation”[tiab] OR “Cystic Fibrosis-gastritis-megaloblastic Anemia Syndrome”[tiab] OR “Cystinosis”[tiab] OR “D-2-hydroxyglutaric Aciduria 1”[tiab] OR “D-glyceric Aciduria”[tiab] OR “Dandy-walker Malformation With Mental Retardation, Macrocephaly, Myopia, And Brachytelephalangy”[tiab] OR “Danon Disease”[tiab] OR “Darier-white Disease”[tiab] OR “De Sanctis-cacchione Syndrome”[tiab] OR “Deafness And Myopia”[tiab] OR “Deafness, Autosomal Recessive 119”[tiab] OR “Deafness, Conductive, With Malformed External Ear”[tiab] OR “Deafness, Congenital, And Onychodystrophy, Autosomal Dominant”[tiab] OR “Deafness, Congenital, With Total Albinism”[tiab] OR “Deafness, Dystonia, And Cerebral Hypomyelination”[tiab] OR “Deafness, Sensorineural, With Pituitary Dwarfism”[tiab] OR “Deafness-enamel Hypoplasia-nail Defects Syndrome”[tiab] OR “Deafness-epiphyseal Dysplasia-short Stature Syndrome”[tiab] OR “Deafness-genital Anomalies-metacarpal And Metatarsal Synostosis Syndrome”[tiab] OR “Deafness-intellectual Disability Syndrome, Martin-probst Type”[tiab] OR “Degcags Syndrome”[tiab] OR “Delayed Speech-facial Asymmetry-strabismus-ear Lobe Creases Syndrome”[tiab] OR “Dent Disease”[tiab] OR “Dentinogenesis Imperfecta-short Stature-hearing Loss-intellectual Disability Syndrome”[tiab] OR “Dermatoleukodystrophy”[tiab] OR “Dermotrichic Syndrome”[tiab] OR “Desbuquois Dysplasia 1”[tiab] OR “Desbuquois Dysplasia 2”[tiab] OR “Desbuquois Syndrome”[tiab] OR “Desmosterolosis”[tiab] OR “Develomental And Epileptic Encephalopathy 94”[tiab] OR “Developmental And Epileptic Encephalopathy 1”[tiab] OR “Developmental And Epileptic Encephalopathy 2”[tiab] OR “Developmental And Epileptic Encephalopathy 58”[tiab] OR “Developmental And Epileptic Encephalopathy 6b, Non-dravet”[tiab] OR “Developmental And Epileptic Encephalopathy 70”[tiab] OR “Developmental And Epileptic Encephalopathy 73”[tiab] OR “Developmental And Epileptic Encephalopathy 89”[tiab] OR “Developmental And Epileptic Encephalopathy 96”[tiab] OR “Developmental And Epileptic Encephalopathy 97”[tiab] OR “Developmental And Epileptic Encephalopathy 99”[tiab] OR “Developmental And Speech Delay Due To Sox5 Deficiency”[tiab] OR “Developmental Delay And Seizures With Or Without Movement Abnormalities”[tiab] OR “Developmental Delay With Dysmorphic Facies And Dental Anomalies”[tiab] OR “Developmental Delay With Or Without Intellectual Impairment Or Behavioral Abnormalities”[tiab] OR “Developmental Delay With Short Stature, Dysmorphic Features, And Sparse Hair”[tiab] OR “Developmental Delay, Hypotonia, Musculoskeletal Defects, And Behavioral Abnormalities”[tiab] OR “Developmental Delay, Impaired Growth, Dysmorphic Facies, And Axonal Neuropathy”[tiab] OR “Developmental Delay, Impaired Speech, And Behavioral Abnormalities”[tiab] OR “Developmental Delay, Intellectual Disability, Obesity, And Dysmorphic Features”[tiab] OR “Developmental Delay-facial Dysmorphism Syndrome Due To Med13l Deficiency”[tiab] OR “Developmental Malformations-deafness-dystonia Syndrome”[tiab] OR “Dextrocardia With Unusual Facies And Microphthalmia”[tiab] OR “Diabetes Insipidus, Nephrogenic, 2”[tiab] OR “Diabetes Insipidus, Nephrogenic, X-linked”[tiab] OR “Diamond-blackfan Anemia 1”[tiab] OR “Dibasic Amino Aciduria I”[tiab] OR “Dicarboxylicamino Aciduria”[tiab] OR “Diets-jongmans Syndrome”[tiab] OR “Digeorge Syndrome”[tiab] OR “Dihydropteridine Reductase Deficiency”[tiab] OR “Dihydropyrimidine Dehydrogenase Deficiency”[tiab] OR “Dihydropyrimidinuria”[tiab] OR “Disorder Of Sex Development-intellectual Disability Syndrome”[tiab] OR “Distal 16p11.2 Microdeletion Syndrome”[tiab] OR “Distal 22q11.2 Microdeletion Syndrome”[tiab] OR “Distal 22q11.2 Microduplication Syndrome”[tiab] OR “Distal 7q11.23 Microdeletion Syndrome”[tiab] OR “Distal 7q11.23 Microduplication Syndrome”[tiab] OR “Distal Limb Deficiencies-micrognathia Syndrome”[tiab] OR “Distal Monosomy 10p”[tiab] OR “Distal Monosomy 10q”[tiab] OR “Distal Monosomy 12q”[tiab] OR “Distal Monosomy 19p13.3”[tiab] OR “Distal Monosomy 1q”[tiab] OR “Distal Monosomy 6p”[tiab] OR “Distal Monosomy 7q36”[tiab] OR “Distal Monosomy 9p”[tiab] OR “Distal Trisomy 15q”[tiab] OR “Distal Trisomy 17q”[tiab] OR “Distal Trisomy 5q”[tiab] OR “Distal Xq28 Microduplication Syndrome”[tiab] OR “Dk1-cdg”[tiab] OR “Donnai-barrow Syndrome”[tiab] OR “Doors Syndrome”[tiab] OR “Dopa-responsive Dystonia Due To Sepiapterin Reductase Deficiency”[tiab] OR “Double Outlet Right Ventricle”[tiab] OR “Down Syndrome”[tiab] OR “Down Syndrometrisomy 21, Included”[tiab] OR “Dpagt1-cdg”[tiab] OR “Dpm3-cdg”[tiab] OR “Dubowitz Syndrome”[tiab] OR “Duchenne And Becker Muscular Dystrophy”[tiab] OR “Duchenne Muscular Dystrophy”[tiab] OR “Duplication Of The Pituitary Gland”[tiab] OR “Dwarfism, Low-birth-weight Type, With Unresponsiveness To Growth Hormone”[tiab] OR “Dwarfism, Mental Retardation, And Eye Abnormality”[tiab] OR “Dyggve-melchior-clausen Disease”[tiab] OR “Dyrk1a-related Intellectual Disability Syndrome”[tiab] OR “Dyrk1a-related Intellectual Disability Syndrome Due To 21q22.13q22.2 Microdeletion”[tiab] OR “Dysautonomia-like Disorder”[tiab] OR “Dysequilibrium Syndrome”[tiab] OR “Dyskeratosis Congenita, Autosomal Recessive 1”[tiab] OR “Dyskeratosis Congenita, Autosomal Recessive, 2”[tiab] OR “Dyskeratosis Congenita, X-linked”[tiab] OR “Dyskinesia, Seizures, And Intellectual Developmental Disorder”[tiab] OR “Dysmorphism-cleft Palate-loose Skin Syndrome”[tiab] OR “Dysmorphism-short Stature-deafness-disorder Of Sex Development Syndrome”[tiab] OR “Dysmyelination With Jaundice”[tiab] OR “Dysosteosclerosis”[tiab] OR “Dysspondyloenchondromatosis”[tiab] OR “Dystonia 16”[tiab] OR “Dystonia 30”[tiab] OR “Dystonia 33”[tiab] OR “Dystonia, Dopa-responsive, Due To Sepiapterin Reductase Deficiency”[tiab] OR “Dystonia, Juvenile-onset”[tiab] OR “Dystonia-parkinsonism-hypermanganesemia Syndrome”[tiab] |
| **E, F, G, H** | “Ear-patella-short Stature Syndrome”[tiab] OR “Early Infantile Epileptic Encephalopathy”[tiab] OR “Early-onset Autosomal Dominant Alzheimer Disease”[tiab] OR “Early-onset Epileptic Encephalopathy And Intellectual Disability Due To Grin2a Mutation”[tiab] OR “Early-onset Epileptic Encephalopathy-cortical Blindness-intellectual Disability-facial Dysmorphism Syndrome”[tiab] OR “Early-onset Parkinsonism-intellectual Disability Syndrome”[tiab] OR “Early-onset Progressive Diffuse Brain Atrophy-microcephaly-muscle Weakness-optic Atrophy Syndrome”[tiab] OR “Early-onset Progressive Encephalopathy-spastic Ataxia-distal Spinal Muscular Atrophy Syndrome”[tiab] OR “Early-onset Seizures-distal Limb Anomalies-facial Dysmorphism-global Developmental Delay Syndrome”[tiab] OR “Early-onset Spastic Ataxia-myoclonic Epilepsy-neuropathy Syndrome”[tiab] OR “Early-onset X-linked Optic Atrophy”[tiab] OR “East Syndrome”[tiab] OR “Ectodermal Dysplasia With Mental Retardation And Syndactyly”[tiab] OR “Ectodermal Dysplasia, Hypohidrotic, With Hypothyroidism And Agenesisof The Corpus Callosum”[tiab] OR “Ectodermal Dysplasia-blindness Syndrome”[tiab] OR “Ectodermal Dysplasia-intellectual Disability-central Nervous System Malformation Syndrome”[tiab] OR “Ectrodactyly, Ectodermal Dysplasia, And Cleft Lip/palate Syndrome 3”[tiab] OR “Ectrodactyly, Ectodermal Dysplasia, And Cleft Lip/palate Syndrome1”[tiab] OR “Edinburgh Malformation Syndrome”[tiab] OR “Eec Syndrome”[tiab] OR “Ehlers-danlos Syndrome, Beasley-cohen Type”[tiab] OR “Ehlers-danlos Syndrome, Musculocontractural Type 1”[tiab] OR “Elejalde Disease”[tiab] OR “Ellis Van Creveld Syndrome”[tiab] OR “Ellis-van Creveld Syndrome”[tiab] OR “Elsahy-waters Syndrome”[tiab] OR “Emanuel Syndrome”[tiab] OR “Encephalocraniocutaneous Lipomatosis”[tiab] OR “Encephalomalacia, Multilocular”[tiab] OR “Encephalopathy Due To Sulfite Oxidase Deficiency”[tiab] OR “Encephalopathy With Intracranial Calcification, Growth Hormone Deficiency, Microcephaly, And Retinal Degeneration”[tiab] OR “Encephalopathy, Acute, Infection-induced (herpes-specific), Susceptibility To, 2”[tiab] OR “Encephalopathy, Ethylmalonic”[tiab] OR “Encephalopathy, Neonatal Severe, Due To Mecp2 Mutations”[tiab] OR “Encephalopathy, Progressive, Early-onset, With Brain Atrophy And Thin Corpus Callosum”[tiab] OR “Encephalopathy, Progressive, With Amyotrophy And Optic Atrophy”[tiab] OR “Enlarged Parietal Foramina”[tiab] OR “Epidermolysis Bullosa, Late-onset Localized Junctional, With Mentalretardation”[tiab] OR “Epilepsy, Early-onset, Vitamin B6-dependent”[tiab] OR “Epilepsy, Familial Adult Myoclonic, 1”[tiab] OR “Epilepsy, Familial Adult Myoclonic, 2”[tiab] OR “Epilepsy, Familial Focal, With Variable Foci”[tiab] OR “Epilepsy, Familial Temporal Lobe, 5”[tiab] OR “Epilepsy, Focal, With Speech Disorder And With Or Without Mental Retardation”[tiab] OR “Epilepsy, Hearing Loss, And Mental Retardation Syndrome”[tiab] OR “Epilepsy, Idiopathic Generalized, Susceptibility To, 12”[tiab] OR “Epilepsy, Idiopathic Generalized, Susceptibility To, 18”[tiab] OR “Epilepsy, Nocturnal Frontal Lobe, 5”[tiab] OR “Epilepsy, Nocturnal Frontal Lobe, Type 1”[tiab] OR “Epilepsy, Photogenic, With Spastic Diplegia And Mental Retardation”[tiab] OR “Epilepsy, Progressive Myoclonic 1a (unverricht And Lundborg)”[tiab] OR “Epilepsy, Progressive Myoclonic 3, With Or Without Intracellular Inclusions”[tiab] OR “Epilepsy, Progressive Myoclonic, 11”[tiab] OR “Epilepsy, Progressive Myoclonic, 8”[tiab] OR “Epilepsy, Pyridoxine-dependent”[tiab] OR “Epilepsy-microcephaly-skeletal Dysplasia Syndrome”[tiab] OR “Epilepsy-telangiectasia”[tiab] OR “Epilepsy-telangiectasia Syndrome”[tiab] OR “Epileptic Encephalopathy, Early Infantile, 13”[tiab] OR “Epileptic Encephalopathy, Early Infantile, 15”[tiab] OR “Epileptic Encephalopathy, Early Infantile, 19”[tiab] OR “Epileptic Encephalopathy, Early Infantile, 24”[tiab] OR “Epileptic Encephalopathy, Early Infantile, 27”[tiab] OR “Epileptic Encephalopathy, Early Infantile, 31”[tiab] OR “Epileptic Encephalopathy, Early Infantile, 32”[tiab] OR “Epileptic Encephalopathy, Early Infantile, 34”[tiab] OR “Epileptic Encephalopathy, Early Infantile, 36”[tiab] OR “Epileptic Encephalopathy, Early Infantile, 37”[tiab] OR “Epileptic Encephalopathy, Early Infantile, 38”[tiab] OR “Epileptic Encephalopathy, Early Infantile, 4”[tiab] OR “Epileptic Encephalopathy, Early Infantile, 40”[tiab] OR “Epileptic Encephalopathy, Early Infantile, 41”[tiab] OR “Epileptic Encephalopathy, Early Infantile, 42”[tiab] OR “Epileptic Encephalopathy, Early Infantile, 43”[tiab] OR “Epileptic Encephalopathy, Early Infantile, 44”[tiab] OR “Epileptic Encephalopathy, Early Infantile, 5”[tiab] OR “Epileptic Encephalopathy, Early Infantile, 53”[tiab] OR “Epileptic Encephalopathy, Early Infantile, 54”[tiab] OR “Epileptic Encephalopathy, Early Infantile, 55”[tiab] OR “Epileptic Encephalopathy, Early Infantile, 56”[tiab] OR “Epileptic Encephalopathy, Early Infantile, 59”[tiab] OR “Epileptic Encephalopathy, Early Infantile, 6 (dravet Syndrome)”[tiab] OR “Epileptic Encephalopathy, Early Infantile, 60”[tiab] OR “Epileptic Encephalopathy, Early Infantile, 61”[tiab] OR “Epileptic Encephalopathy, Early Infantile, 63”[tiab] OR “Epileptic Encephalopathy, Early Infantile, 64”[tiab] OR “Epileptic Encephalopathy, Early Infantile, 66”[tiab] OR “Epileptic Encephalopathy, Early Infantile, 7”[tiab] OR “Epileptic Encephalopathy, Early Infantile, 76”[tiab] OR “Epileptic Encephalopathy, Early Infantile, 79”[tiab] OR “Epileptic Encephalopathy, Early Infantile, 8”[tiab] OR “Epileptic Encephalopathy, Early Infantile, 81”[tiab] OR “Epileptic Encephalopathy, Early Infantile, 82”[tiab] OR “Epileptic Encephalopathy, Early Infantile, 83”[tiab] OR “Epileptic Encephalopathy, Early Infantile, 87”[tiab] OR “Epileptic Encephalopathy, Early Infantile, 9”[tiab] OR “Epileptic Encephalopathy, Infantile Or Early Childhood, 1”[tiab] OR “Epileptic Encephalopathy, Infantile Or Early Childhood, 2”[tiab] OR “Epileptic Encephalopathy, Infantile Or Early Childhood, 3”[tiab] OR “Epiphyseal Dysplasia-hearing Loss-dysmorphism Syndrome”[tiab] OR “Ermine Phenotype”[tiab] OR “Erythrokeratodermia Variabilis”[tiab] OR “Ethylmalonic Encephalopathy”[tiab] OR “Extrasystoles, Multiform Ventricular, With Short Stature, Hyperpigmentationand Microcephaly”[tiab] OR “Facial Abnormalities, Kyphoscoliosis, And Mental Retardation”[tiab] OR “Facial Dysmorphism-developmental Delay-behavioral Abnormalities Syndrome Due To 10p11.21p12.31 Microdeletion”[tiab] OR “Facial Dysmorphism-developmental Delay-behavioral Abnormalities Syndrome Due To Wac Point Mutation”[tiab] OR “Facial Dysmorphism-macrocephaly-myopia-dandy-walker Malformation Syndrome”[tiab] OR “Facial Dysmorphism-shawl Scrotum-joint Laxity Syndrome”[tiab] OR “Faciocardiomelic Syndrome”[tiab] OR “Faciocardiorenal Syndrome”[tiab] OR “Faciodigitogenital Syndrome, Autosomal Recessive”[tiab] OR “Facioscapulohumeral Muscular Dystrophy 1”[tiab] OR “Fallot Complex With Severe Mental And Growth Retardation”[tiab] OR “Fallot Complex-intellectual Disability-growth Delay Syndrome”[tiab] OR “Familial Acute Necrotizing Encephalopathy”[tiab] OR “Familial Adenomatous Polyposis Due To 5q22.2 Microdeletion”[tiab] OR “Familial Congenital Mirror Movements”[tiab] OR “Familial Exudative Vitreoretinopathy”[tiab] OR “Familial Focal Epilepsy With Variable Foci”[tiab] OR “Familial Glucocorticoid Deficiency”[tiab] OR “Familial Infantile Bilateral Striatal Necrosis”[tiab] OR “Familial Infantile Myoclonic Epilepsy”[tiab] OR “Familial Multiple Nevi Flammei”[tiab] OR “Familial Or Sporadic Hemiplegic Migraine”[tiab] OR “Familial Paroxysmal Ataxia”[tiab] OR “Familial Scaphocephaly Syndrome, Mcgillivray Type”[tiab] OR “Familial Thyroid Dyshormonogenesis”[tiab] OR “Fanconi Anemia”[tiab] OR “Fanconi Anemia, Complementation Group B”[tiab] OR “Fanconi Anemia, Complementation Group C”[tiab] OR “Fanconi Anemia, Complementation Group D2”[tiab] OR “Fanconi Anemia, Complementation Group E”[tiab] OR “Fanconi Anemia, Complementation Group R”[tiab] OR “Fanconi Anemia, Complementation Group S”[tiab] OR “Farber Disease”[tiab] OR “Farber Lipogranulomatosis”[tiab] OR “Fatal Infantile Lactic Acidosis With Methylmalonic Aciduria”[tiab] OR “Fatty Acyl-coa Reductase 1 Deficiency”[tiab] OR “Fbln1-related Developmental Delay-central Nervous System Anomaly-syndactyly Syndrome”[tiab] OR “Feingold Syndrome”[tiab] OR “Feingold Syndrome 1”[tiab] OR “Feingold Syndrome 2”[tiab] OR “Feingold Syndrome Type 1”[tiab] OR “Feingold Syndrome Type 2”[tiab] OR “Female Restricted Epilepsy With Intellectual Disability”[tiab] OR “Fetal Alcohol Syndrome”[tiab] OR “Fetal Iodine Deficiency Disorder”[tiab] OR “Fetal Iodine Syndrome”[tiab] OR “Fetal Trimethadione Syndrome”[tiab] OR “Fg Syndrome 4”[tiab] OR “Fg Syndrome Type 1”[tiab] OR “Fibrodysplasia Ossificans Progressiva”[tiab] OR “Fibromatosis, Gingival, 1”[tiab] OR “Fibrosis Of Extraocular Muscles, Congenital, 3b”[tiab] OR “Fibular Hemimelia”[tiab] OR “Filippi Syndrome”[tiab] OR “Floating-harbor Syndrome”[tiab] OR “Focal Cortical Dysplasia Of Taylor”[tiab] OR “Focal Dermal Hypoplasia”[tiab] OR “Focal Segmental Glomerulosclerosis And Neurodevelopmental Syndrome”[tiab] OR “Folate Malabsorption, Hereditary”[tiab] OR “Formiminoglutamic Aciduria”[tiab] OR “Formiminotransferase Deficiency”[tiab] OR “Fountain Syndrome”[tiab] OR “Foxg1 Syndrome Due To 14q12 Microdeletion”[tiab] OR “Fragile X Mental Retardation Syndrome”[tiab] OR “Fragile X Syndrome”[tiab] OR “Fraser Syndrome”[tiab] OR “Fraxe Intellectual Disability”[tiab] OR “Free Sialic Acid Storage Disease”[tiab] OR “Fried Syndrome”[tiab] OR “Frontometaphyseal Dysplasia”[tiab] OR “Frontometaphyseal Dysplasia 2”[tiab] OR “Frontonasal Dysplasia 1”[tiab] OR “Frontonasal Dysplasia 2”[tiab] OR “Frontonasal Dysplasia 3”[tiab] OR “Frontonasal Dysplasia-alopecia-genital Anomalies Syndrome”[tiab] OR “Frontonasal Dysplasia-severe Microphthalmia-severe Facial Clefting Syndrome”[tiab] OR “Frontoocular Syndrome”[tiab] OR “Fructose Intolerance, Hereditary”[tiab] OR “Fructose-1,6-bisphosphatase Deficiency”[tiab] OR “Fryns Macrocephaly”[tiab] OR “Fryns Syndrome”[tiab] OR “Fryns-smeets-thiry Syndrome”[tiab] OR “Fucosidosis”[tiab] OR “Fumarase Deficiency”[tiab] OR “Gabriele-de Vries Syndrome”[tiab] OR “Galactokinase Deficiency”[tiab] OR “Galactose Epimerase Deficiency”[tiab] OR “Galactosemia”[tiab] OR “Galactosialidosis”[tiab] OR “Galloway-mowat Syndrome 1”[tiab] OR “Galloway-mowat Syndrome 2, X-linked”[tiab] OR “Galloway-mowat Syndrome 3”[tiab] OR “Galloway-mowat Syndrome 6”[tiab] OR “Galloway-mowat Syndrome 7”[tiab] OR “Gapo Syndrome”[tiab] OR “Gardner Syndrome”[tiab] OR “Gastrointestinal Defects And Immunodeficiency Syndrome 2”[tiab] OR “Gaucher Disease-ophthalmoplegia-cardiovascular Calcification Syndrome”[tiab] OR “Gaze Palsy, Familial Horizontal, With Progressive Scoliosis, 2”[tiab] OR “Gemignani Syndrome”[tiab] OR “Generalized Epilepsy With Febrile Seizures Plus, Type 10”[tiab] OR “Generalized Epilepsy-paroxysmal Dyskinesia Syndrome”[tiab] OR “Genitopatellar Syndrome”[tiab] OR “German Syndrome”[tiab] OR “Geroderma Osteodysplastica”[tiab] OR “Geroderma Osteodysplasticum”[tiab] OR “Giant Axonal Neuropathy”[tiab] OR “Giant Axonal Neuropathy 1, Autosomal Recessive”[tiab] OR “Gillespie Syndrome”[tiab] OR “Glass Syndrome”[tiab] OR “Global Developmental Delay With Or Without Impaired Intellectual Development”[tiab] OR “Global Developmental Delay With Speech And Behavioral Abnormalities”[tiab] OR “Global Developmental Delay, Absent Or Hypoplastic Corpus Callosum, And Dysmorphic Facies”[tiab] OR “Global Developmental Delay-neuro-ophthalmological Abnormalities-seizures-intellectual Disability Syndrome”[tiab] OR “Global Developmental Delay-visual Anomalies-progressive Cerebellar Atrophy-truncal Hypotonia Syndrome”[tiab] OR “Glut1 Deficiency Syndrome 1”[tiab] OR “Glutamate-cysteine Ligase Deficiency”[tiab] OR “Glutathione Synthetase Deficiency”[tiab] OR “Glutathionuria”[tiab] OR “Glycerol Kinase Deficiency”[tiab] OR “Glycine Encephalopathy”[tiab] OR “Glycogen Storage Disease Due To Acid Maltase Deficiency, Infantile Onset”[tiab] OR “Glycogen Storage Disease Due To Aldolase A Deficiency”[tiab] OR “Glycogen Storage Disease Due To Glycogen Debranching Enzyme Deficiency”[tiab] OR “Glycogen Storage Disease Due To Lamp-2 Deficiency”[tiab] OR “Glycogen Storage Disease Due To Liver Phosphorylase Kinase Deficiency”[tiab] OR “Glycogen Storage Disease Due To Phosphoglycerate Kinase 1 Deficiency”[tiab] OR “Glycogen Storage Disease Xii”[tiab] OR “Glycosylphosphatidylinositol Biosynthesis Defect 11”[tiab] OR “Glycosylphosphatidylinositol Biosynthesis Defect 15”[tiab] OR “Glycosylphosphatidylinositol Biosynthesis Defect 16”[tiab] OR “Gm1 Gangliosidosis Type 1”[tiab] OR “Gm1-gangliosidosis, Type I”[tiab] OR “Gm1-gangliosidosis, Type Iii”[tiab] OR “Gmppb-related Limb-girdle Muscular Dystrophy R19”[tiab] OR “Gms Syndrome”[tiab] OR “Gnb5-related Intellectual Disability-cardiac Arrhythmia Syndrome”[tiab] OR “Goldberg-shprintzen Megacolon Syndrome”[tiab] OR “Goldberg-shprintzen Syndrome”[tiab] OR “Gombo Syndrome”[tiab] OR “Gómez-lópez-hernández Syndrome”[tiab] OR “Gonadal Dysgenesis, Xy Type, With Associated Anomalies”[tiab] OR “Gorlin Syndrome”[tiab] OR “Gorlin-chaudhry-moss Syndrome”[tiab] OR “Grange Syndrome”[tiab] OR “Greig Cephalopolysyndactyly Syndrome”[tiab] OR “Griscelli Syndrome”[tiab] OR “Griscelli Syndrome Type 1”[tiab] OR “Griscelli Syndrome, Type 1”[tiab] OR “Growth Delay Due To Insulin-like Growth Factor I Resistance”[tiab] OR “Growth Delay Due To Insulin-like Growth Factor Type 1 Deficiency”[tiab] OR “Growth Delay-intellectual Disability-hepatopathy Syndrome”[tiab] OR “Growth Failure, Microcephaly, Mental Retardation, Cataracts, Largejoint Contractures, Osteoporosis, Cortical Dysplasia, And Cerebellaratrophy”[tiab] OR “Growth Hormone Insensitivity Syndrome”[tiab] OR “Growth Retardation, Deafness, Femoral Epiphyseal Dysplasia, And Lacrimal Duct Obstruction”[tiab] OR “Growth Retardation, Impaired Intellectual Development, Hypotonia, And Hepatopathy”[tiab] OR “Guanidinoacetate Methyltransferase Deficiency”[tiab] OR “Gurrieri Syndrome”[tiab] OR “H Syndrome”[tiab] OR “Haddad Syndrome”[tiab] OR “Hadziselimovic Syndrome”[tiab] OR “Hair Defect With Photosensitivity And Mental Retardation”[tiab] OR “Hall-riggs Mental Retardation Syndrome”[tiab] OR “Hall-riggs Syndrome”[tiab] OR “Hallermann-streiff Syndrome”[tiab] OR “Hamel Cerebro-palato-cardiac Syndrome”[tiab] OR “Hand And Foot Deformity With Flat Facies”[tiab] OR “Harel-yoon Syndrome”[tiab] OR “Harrod Syndrome”[tiab] OR “Hartnup Disease”[tiab] OR “Heart-hand Syndrome Type 2”[tiab] OR “Helsmoortel-van Der Aa Syndrome”[tiab] OR “Hemifacial Microsomia”[tiab] OR “Hemihyperplasia, Isolated”[tiab] OR “Hemimegalencephaly”[tiab] OR “Hemolytic Anemia, Nonspherocytic, Due To Glucose Phosphate Isomerasedeficiency”[tiab] OR “Hengel-maroofian-schols Syndrome”[tiab] OR “Hennekam Lymphangiectasia-lymphedema Syndrome”[tiab] OR “Hennekam Lymphangiectasia-lymphedema Syndrome 2”[tiab] OR “Hennekam Syndrome”[tiab] OR “Hennekam-beemer Syndrome”[tiab] OR “Hepatic Fibrosis-renal Cysts-intellectual Disability Syndrome”[tiab] OR “Hereditary Bullous Dystrophy, Macular Type”[tiab] OR “Hereditary Cryohydrocytosis With Reduced Stomatin”[tiab] OR “Hereditary Hyperekplexia”[tiab] OR “Hereditary Methemoglobinemia”[tiab] OR “Hereditary Sensory And Autonomic Neuropathy Due To Tecpr2 Mutation”[tiab] OR “Hereditary Sensory And Autonomic Neuropathy Type 4”[tiab] OR “Hereditary Sensory And Autonomic Neuropathy Type 5”[tiab] OR “Hermansky-pudlak Syndrome 2”[tiab] OR “Heterotopia, Periventricular, X-linked Dominant”[tiab] OR “Hiatt-neu-cooper Neurodevelopmental Syndrome”[tiab] OR “Hidrotic Ectodermal Dysplasia, Halal Type”[tiab] OR “Hirschsprung Disease”[tiab] OR “Hirschsprung Disease-deafness-polydactyly Syndrome”[tiab] OR “Hirsutism, Skeletal Dysplasia, And Mental Retardation”[tiab] OR “Histidinemia”[tiab] OR “Histidinuria Due To A Renal Tubular Defect”[tiab] OR “Histidinuria-renal Tubular Defect Syndrome”[tiab] OR “Hnf1b-related Autosomal Dominant Tubulointerstitial Kidney Disease”[tiab] OR “Holoprosencephaly 1”[tiab] OR “Holoprosencephaly 13, X-linked”[tiab] OR “Holoprosencephaly 2”[tiab] OR “Holoprosencephaly 3”[tiab] OR “Holoprosencephaly 5”[tiab] OR “Holoprosencephaly, Recurrent Infections, And Monocytosis”[tiab] OR “Homocarnosinosis”[tiab] OR “Homocystinuria Due To Cystathionine Beta-synthase Deficiency”[tiab] OR “Homocystinuria Due To Methylene Tetrahydrofolate Reductase Deficiency”[tiab] OR “Homocystinuria-megaloblastic Anemia, Cbl E Type”[tiab] OR “Homocystinuria-megaloblastic Anemia, Cblg Complementation Type”[tiab] OR “Hooft Disease”[tiab] OR “Hoyeraal-hreidarsson Syndrome”[tiab] OR “Hsd10 Disease”[tiab] OR “Hsd10 Disease, Atypical Type”[tiab] OR “Hsd10 Disease, Infantile Type”[tiab] OR “Hsd10 Mitochondrial Disease”[tiab] OR “Hunter-mcalpine Craniosynostosis Syndrome”[tiab] OR “Hurler Syndrome”[tiab] OR “Hutterite Cerebroosteonephrodysplasia Syndrome”[tiab] OR “Hydrocephalus Due To Congenital Stenosis Of Aqueduct Of Sylvius”[tiab] OR “Hydrocephalus With Stenosis Of The Aqueduct Of Sylvius”[tiab] OR “Hydrocephalus, Congenital, 2, With Or Without Brain Or Eye Anomalies”[tiab] OR “Hydrocephalus, Nonsyndromic, Autosomal Recessive 1”[tiab] OR “Hydrocephalus, Skeletal Anomalies, And Mental Disturbance”[tiab] OR “Hydrocephalus-costovertebral Dysplasia-sprengel Anomaly Syndrome”[tiab] OR “Hydrocephalus-obesity-hypogonadism Syndrome”[tiab] OR “Hydrocephaly-cerebellar Agenesis Syndrome”[tiab] OR “Hydroxykynureninuria”[tiab] OR “Hydroxylysinuria”[tiab] OR “Hydroxyprolinemia”[tiab] OR “Hypercalcemia, Infantile, 1”[tiab] OR “Hyperinsulinemic Hypoglycemia, Familial, 1”[tiab] OR “Hyperinsulinemic Hypoglycemia, Familial, 3”[tiab] OR “Hyperinsulinemic Hypoglycemia, Familial, 4”[tiab] OR “Hyperinsulinemic Hypoglycemia, Familial, 6”[tiab] OR “Hyperinsulinism Due To Hnf4a Deficiency”[tiab] OR “Hyperinsulinism-hyperammonemia Syndrome”[tiab] OR “Hyperleucine-isoleucinemia”[tiab] OR “Hyperlysinemia”[tiab] OR “Hyperlysinemia Due To Defect In Lysine Transport Into Mitochondria”[tiab] OR “Hyperlysinemia, Type I”[tiab] OR “Hyperlysinuria With Hyperammonemia”[tiab] OR “Hypermanganesemia With Dystonia 2”[tiab] OR “Hypermethioninemia With S-adenosylhomocysteine Hydrolase Deficiency”[tiab] OR “Hyperornithinemia-hyperammonemia-homocitrullinuria Syndrome”[tiab] OR “Hyperphenylalaninemia, Bh4-deficient, A”[tiab] OR “Hyperphenylalaninemia, Bh4-deficient, B”[tiab] OR “Hyperphenylalaninemia, Bh4-deficient, C”[tiab] OR “Hyperphenylalaninemia, Mild, Non-bh4-deficient”[tiab] OR “Hyperphosphatasia With Mental Retardation”[tiab] OR “Hyperphosphatasia With Mental Retardation Syndrome 2”[tiab] OR “Hyperphosphatasia With Mental Retardation Syndrome 3”[tiab] OR “Hyperphosphatasia With Mental Retardation Syndrome 4”[tiab] OR “Hyperphosphatasia-intellectual Disability Syndrome”[tiab] OR “Hyperprolinemia Type 2”[tiab] OR “Hyperprolinemia, Type I”[tiab] OR “Hyperprolinemia, Type Ii”[tiab] OR “Hypertelorism And Other Facial Dysmorphism, Brachydactyly, Genital Abnormalities, Mental Retardation, And Recurrent Inflammatory Episodes”[tiab] OR “Hypertelorism And Tetralogy Of Fallot”[tiab] OR “Hypertelorism-microtia-facial Clefting Syndrome”[tiab] OR “Hyperthyroidism, Nonautoimmune”[tiab] OR “Hypertrichosis Cubiti”[tiab] OR “Hypertrichotic Osteochondrodysplasia”[tiab] OR “Hypertryptophanemia”[tiab] OR “Hyperuricemia, Infantile, With Abnormal Behavior And Normal Hypoxanthineguanine Phosphoribosyltransferase”[tiab] OR “Hypo- And Hypermelanotic Cutaneous Macules-retarded Growth-intellectual Disability Syndrome”[tiab] OR “Hypochondroplasia”[tiab] OR “Hypoglossia-hypodactyly Syndrome”[tiab] OR “Hypoglycemia Of Infancy, Leucine-sensitive”[tiab] OR “Hypogonadism With Low-grade Mental Deficiency And Microcephaly”[tiab] OR “Hypogonadism, Male, With Mental Retardation And Skeletal Anomalies”[tiab] OR “Hypogonadism-mitral Valve Prolapse-intellectual Disability Syndrome”[tiab] OR “Hypogonadotropic Hypogonadism 10 With Or Without Anosmia”[tiab] OR “Hypogonadotropic Hypogonadism 2 With Or Without Anosmia”[tiab] OR “Hypogonadotropic Hypogonadism 26 With Or Without Anosmia”[tiab] OR “Hypohidrosis-enamel Hypoplasia-palmoplantar Keratoderma-intellectual Disability Syndrome”[tiab] OR “Hypomagnesemia 4, Renal”[tiab] OR “Hypomagnesemia, Seizures, And Mental Retardation”[tiab] OR “Hypomelanosis Of Ito”[tiab] OR “Hypomyelination With Brainstem And Spinal Cord Involvement And Legspasticity”[tiab] OR “Hypomyelination-congenital Cataract Syndrome”[tiab] OR “Hypoparathyroidism-retardation-dysmorphism Syndrome”[tiab] OR “Hypospadias-intellectual Disability, Goldblatt Type Syndrome”[tiab] OR “Hypospadias-mental Retardation Syndrome”[tiab] OR “Hypothyroidism, Congenital, Nongoitrous, 4”[tiab] OR “Hypothyroidism, Congenital, Nongoitrous, 5”[tiab] OR “Hypotonia, Ataxia, And Delayed Development Syndrome”[tiab] OR “Hypotonia, Ataxia, Developmental Delay, And Tooth Enamel Defect Syndrome”[tiab] OR “Hypotonia, Hypoventilation, Impaired Intellectual Development, Dysautonomia, Epilepsy, And Eye Abnormalities”[tiab] OR “Hypotonia, Infantile, With Psychomotor Retardation And Characteristic Facies 2”[tiab] OR “Hypotonia-cystinuria Syndrome”[tiab] OR “Hypotonia-speech Impairment-severe Cognitive Delay Syndrome”[tiab] OR “Hypotrichosis-intellectual Disability, Lopes Type”[tiab] OR “Hypoxanthine Guanine Phosphoribosyltransferase Partial Deficiency”[tiab] |
| **I, J, K** | “Icf Syndrome”[tiab] OR “Ichthyosiform Erythroderma, Corneal Involvement, And Deafness”[tiab] OR “Ichthyosis And Male Hypogonadism”[tiab] OR “Ichthyosis Follicularis-alopecia-photophobia Syndrome”[tiab] OR “Ichthyosis With Alopecia, Eclabion, Ectropion, And Mental Retardation”[tiab] OR “Ichthyosis, Congenital, Autosomal Recessive 2”[tiab] OR “Ichthyosis, Mental Retardation, Dwarfism, And Renal Impairment”[tiab] OR “Ichthyosis, Spastic Quadriplegia, And Mental Retardation”[tiab] OR “Ichthyosis, Split Hairs, And Amino Aciduria”[tiab] OR “Ichthyosis-alopecia-eclabion-ectropion-intellectual Disability Syndrome”[tiab] OR “Ichthyosis-intellectual Disability-dwarfism-renal Impairment Syndrome”[tiab] OR “Ichthyosis-mental Retardation Syndrome With Large Keratohyalin Granules In The Skin”[tiab] OR “Ifap Syndrome With Or Without Bresheck Syndrome”[tiab] OR “Imagawa-matsumoto Syndrome”[tiab] OR “Iminoglycinuria”[tiab] OR “Immunodeficiency 23”[tiab] OR “Immunodeficiency 47”[tiab] OR “Immunodeficiency 49”[tiab] OR “Immunodeficiency Due To Purine Nucleoside Phosphorylase Deficiency”[tiab] OR “Immunodeficiency, Developmental Delay, And Hypohomocysteinemia”[tiab] OR “Immunodeficiency-centromeric Instability-facial Anomalies Syndrome”[tiab] OR “Immunodeficiency-centromeric Instability-facial Anomalies Syndrome 3”[tiab] OR “Immunodeficiency-centromeric Instability-facial Anomalies Syndrome 4”[tiab] OR “Immunodeficiency-centromeric Instability-facial Anomalies Syndrome2”[tiab] OR “Immunoskeletal Dysplasia With Neurodevelopmental Abnormalities”[tiab] OR “Inclusion Body Myopathy With Paget Disease Of Bone And Frontotemporal Dementia”[tiab] OR “Incontinentia Pigmenti”[tiab] OR “Indolylacroyl Glycinuria With Mental Retardation”[tiab] OR “Infantile Cerebellar-retinal Degeneration”[tiab] OR “Infantile Choroidocerebral Calcification Syndrome”[tiab] OR “Infantile Multisystem Neurologic-endocrine-pancreatic Disease”[tiab] OR “Insensitivity To Pain, Congenital, With Anhidrosis”[tiab] OR “Insulin-like Growth Factor I Deficiency”[tiab] OR “Insulin-like Growth Factor I, Resistance To”[tiab] OR “Intellectual Developmental Disorder 60 With Seizures”[tiab] OR “Intellectual Developmental Disorder 62”[tiab] OR “Intellectual Developmental Disorder And Hypogonadotropic Hypogonadism”[tiab] OR “Intellectual Developmental Disorder And Retinitis Pigmentosa”[tiab] OR “Intellectual Developmental Disorder With Abnormal Behavior, Microcephaly, And Short Stature”[tiab] OR “Intellectual Developmental Disorder With Autism And Speech Delay”[tiab] OR “Intellectual Developmental Disorder With Autistic Features And Language Delay, With Or Without Seizures”[tiab] OR “Intellectual Developmental Disorder With Behavioral Abnormalities And Craniofacial Dysmorphism With Or Without Seizures”[tiab] OR “Intellectual Developmental Disorder With Cardiac Arrhythmia”[tiab] OR “Intellectual Developmental Disorder With Dysmorphic Facies And Behavioral Abnormalities”[tiab] OR “Intellectual Developmental Disorder With Dysmorphic Facies And Ptosis”[tiab] OR “Intellectual Developmental Disorder With Dysmorphic Facies, Seizures, And Distal Limb Anomalies”[tiab] OR “Intellectual Developmental Disorder With Epilepsy, Behavioral Abnormalities, And Coarse Facies”[tiab] OR “Intellectual Developmental Disorder With Gastrointestinal Difficulties And High Pain Threshold”[tiab] OR “Intellectual Developmental Disorder With Hypotonia And Behavioral Abnormalities”[tiab] OR “Intellectual Developmental Disorder With Hypotonia, Impaired Speech, And Dysmorphic Facies”[tiab] OR “Intellectual Developmental Disorder With Impaired Language And Dysmorphic Facies”[tiab] OR “Intellectual Developmental Disorder With Macrocephaly, Seizures, And Speech Delay”[tiab] OR “Intellectual Developmental Disorder With Neuropsychiatric Features”[tiab] OR “Intellectual Developmental Disorder With Or Without Epilepsy Or Cerebellar Ataxia”[tiab] OR “Intellectual Developmental Disorder With Paroxysmal Dyskinesia Or Seizures”[tiab] OR “Intellectual Developmental Disorder With Persistence Of Fetal Hemoglobin”[tiab] OR “Intellectual Developmental Disorder With Poor Growth And With Or Without Seizures Or Ataxia”[tiab] OR “Intellectual Developmental Disorder With Seizures And Language Delay”[tiab] OR “Intellectual Developmental Disorder With Short Stature And Behavioral Abnormalities”[tiab] OR “Intellectual Developmental Disorder With Short Stature And Variable Skeletal Anomalies”[tiab] OR “Intellectual Developmental Disorder With Speech Delay And Axonal Peripheral Neuropathy”[tiab] OR “Intellectual Developmental Disorder With Speech Delay, Autism, And Dysmorphic Facies”[tiab] OR “Intellectual Developmental Disorder With Speech Delay, Dysmorphic Facies, And T-cell Abnormalities”[tiab] OR “Intellectual Developmental Disorder, Autosomal Dominant 63, With Macrocephaly”[tiab] OR “Intellectual Developmental Disorder, Autosomal Dominant 64”[tiab] OR “Intellectual Developmental Disorder, Autosomal Dominant 65”[tiab] OR “Intellectual Developmental Disorder, Autosomal Recessive 40”[tiab] OR “Intellectual Developmental Disorder, Autosomal Recessive 70”[tiab] OR “Intellectual Developmental Disorder, Autosomal Recessive 72”[tiab] OR “Intellectual Developmental Disorder, Autosomal Recessive 73”[tiab] OR “Intellectual Developmental Disorder, Autosomal Recessive 74”[tiab] OR “Intellectual Developmental Disorder, X-linked, Syndrome, Snijders Blok Type”[tiab] OR “Intellectual Developmental Disorder, X-linked, Syndromic, With Pigmentary Mosaicism And Coarse Facies”[tiab] OR “Intellectual Disability Syndrome Due To A Dyrk1a Point Mutation”[tiab] OR “Intellectual Disability, Buenos-aires Type”[tiab] OR “Intellectual Disability, Wolff Type”[tiab] OR “Intellectual Disability-alacrima-achalasia Syndrome”[tiab] OR “Intellectual Disability-autism-speech Apraxia-craniofacial Dysmorphism Syndrome”[tiab] OR “Intellectual Disability-balding-patella Luxation-acromicria Syndrome”[tiab] OR “Intellectual Disability-brachydactyly-pierre Robin Syndrome”[tiab] OR “Intellectual Disability-cardiac Anomalies-short Stature-joint Laxity Syndrome”[tiab] OR “Intellectual Disability-cataracts-calcified Pinnae-myopathy Syndrome”[tiab] OR “Intellectual Disability-cataracts-kyphosis Syndrome”[tiab] OR “Intellectual Disability-coarse Face-macrocephaly-cerebellar Hypotrophy Syndrome”[tiab] OR “Intellectual Disability-craniofacial Dysmorphism-cryptorchidism Syndrome”[tiab] OR “Intellectual Disability-developmental Delay-contractures Syndrome”[tiab] OR “Intellectual Disability-dysmorphism-hypogonadism-diabetes Mellitus Syndrome”[tiab] OR “Intellectual Disability-epilepsy-extrapyramidal Syndrome”[tiab] OR “Intellectual Disability-expressive Aphasia-facial Dysmorphism Syndrome”[tiab] OR “Intellectual Disability-facial Dysmorphism Syndrome Due To Setd5 Haploinsufficiency”[tiab] OR “Intellectual Disability-facial Dysmorphism-hand Anomalies Syndrome”[tiab] OR “Intellectual Disability-hyperkinetic Movement-truncal Ataxia Syndrome”[tiab] OR “Intellectual Disability-hypoplastic Corpus Callosum-preauricular Tag Syndrome”[tiab] OR “Intellectual Disability-macrocephaly-hypotonia-behavioral Abnormalities Syndrome”[tiab] OR “Intellectual Disability-muscle Weakness-short Stature-facial Dysmorphism Syndrome”[tiab] OR “Intellectual Disability-myopathy-short Stature-endocrine Defect Syndrome”[tiab] OR “Intellectual Disability-obesity-brain Malformations-facial Dysmorphism Syndrome”[tiab] OR “Intellectual Disability-obesity-prognathism-eye And Skin Anomalies Syndrome”[tiab] OR “Intellectual Disability-polydactyly-uncombable Hair Syndrome”[tiab] OR “Intellectual Disability-seizures-abnormal Gait-facial Dysmorphism Syndrome”[tiab] OR “Intellectual Disability-seizures-hypophosphatasia-ophthalmic-skeletal Anomalies Syndrome”[tiab] OR “Intellectual Disability-seizures-macrocephaly-obesity Syndrome”[tiab] OR “Intellectual Disability-severe Speech Delay-mild Dysmorphism Syndrome”[tiab] OR “Intellectual Disability-short Stature-hypertelorism Syndrome”[tiab] OR “Intellectual Disability-spasticity-ectrodactyly Syndrome”[tiab] OR “Intellectual Disability-strabismus Syndrome”[tiab] OR “Irida Syndrome”[tiab] OR “Isolated Focal Cortical Dysplasia”[tiab] OR “Isolated Glycerol Kinase Deficiency”[tiab] OR “Isolated Hemihyperplasia”[tiab] OR “Isolated Lissencephaly Type 1 Without Known Genetic Defects”[tiab] OR “Isolated Permanent Neonatal Diabetes Mellitus”[tiab] OR “Jaberi-elahi Syndrome”[tiab] OR “Jacobsen Syndrome”[tiab] OR “Japanese Encephalitis”[tiab] OR “Jawad Syndrome”[tiab] OR “Jeavons Syndrome”[tiab] OR “Johanson-blizzard Syndrome”[tiab] OR “Johnson Neuroectodermal Syndrome”[tiab] OR “Joubert Syndrome”[tiab] OR “Joubert Syndrome 1”[tiab] OR “Joubert Syndrome 10”[tiab] OR “Joubert Syndrome 14”[tiab] OR “Joubert Syndrome 15”[tiab] OR “Joubert Syndrome 18”[tiab] OR “Joubert Syndrome 2”[tiab] OR “Joubert Syndrome 21”[tiab] OR “Joubert Syndrome 27”[tiab] OR “Joubert Syndrome 28”[tiab] OR “Joubert Syndrome 3”[tiab] OR “Joubert Syndrome 30”[tiab] OR “Joubert Syndrome 32”[tiab] OR “Joubert Syndrome 36”[tiab] OR “Joubert Syndrome 40”[tiab] OR “Joubert Syndrome 5”[tiab] OR “Joubert Syndrome 6”[tiab] OR “Joubert Syndrome 7”[tiab] OR “Joubert Syndrome 8”[tiab] OR “Joubert Syndrome 9”[tiab] OR “Joubert Syndrome With Hepatic Defect”[tiab] OR “Joubert Syndrome With Ocular Defect”[tiab] OR “Joubert Syndrome With Oculorenal Defect”[tiab] OR “Joubert Syndrome With Renal Defect”[tiab] OR “Juberg-hayward Syndrome”[tiab] OR “Jung Syndrome”[tiab] OR “Juvenile Polyposis Of Infancy”[tiab] OR “Juvenile Polyposis Syndrome”[tiab] OR “Juvenile Sialidosis Type 2”[tiab] OR “Juvenile-onset Diabetes Mellitus-central And Peripheral Neurodegeneration Syndrome”[tiab] OR “Kabuki Syndrome 1”[tiab] OR “Kabuki Syndrome 2”[tiab] OR “Kagami-ogata Syndrome”[tiab] OR “Kagami-ogata Syndrome Due To Maternal 14q32.2 Hypermethylation”[tiab] OR “Kahrizi Syndrome”[tiab] OR “Kallmann Syndrome-heart Disease Syndrome”[tiab] OR “Kanzaki Disease”[tiab] OR “Kapur-toriello Syndrome”[tiab] OR “Kaufman Oculocerebrofacial Syndrome”[tiab] OR “Kbg Syndrome”[tiab] OR “Kcnq2-related Epileptic Encephalopathy”[tiab] OR “Kdm5c-related Syndromic X-linked Intellectual Disability”[tiab] OR “Keipert Syndrome”[tiab] OR “Kennerknecht Syndrome”[tiab] OR “Keppen-lubinsky Syndrome”[tiab] OR “Keutel Syndrome”[tiab] OR “Kifafa Seizure Disorder”[tiab] OR “Kilquist Syndrome”[tiab] OR “Kinsship Syndrome”[tiab] OR “Kleefstra Syndrome”[tiab] OR “Kleefstra Syndrome 2”[tiab] OR “Kleefstra Syndrome Due To 9q34 Microdeletion”[tiab] OR “Kleefstra Syndrome Due To A Point Mutation”[tiab] OR “Klippel-trénaunay Syndrome”[tiab] OR “Klippel-trenaunay-weber Syndrome”[tiab] OR “Knobloch Syndrome 1”[tiab] OR “Kohlschutter-tonz Syndrome”[tiab] OR “Kohlschutter-tonz Syndrome-like”[tiab] OR “Koolen-de Vries Syndrome”[tiab] OR “Koolen-de Vries Syndrome Due To A Point Mutation”[tiab] OR “Kufor-rakeb Syndrome”[tiab] OR “Kyphoscoliosis-lateral Tongue Atrophy-hereditary Spastic Paraplegia Syndrome”[tiab] OR “L-2-hydroxyglutaric Aciduria”[tiab] OR “L1 Syndrome”[tiab] OR “Lactic Aciduria Due To D-lactic Acid”[tiab] OR “Lamb-shaffer Syndrome”[tiab] OR “Lambert Syndrome”[tiab] OR “Laminin Subunit Alpha 2-related Congenital Muscular Dystrophy”[tiab] OR “Language Delay And Attention Deficit-hyperactivity Disorder/cognitive Impairment With Or Without Cardiac Arrhythmia”[tiab] OR “Laron Syndrome”[tiab] OR “Larsen Syndrome”[tiab] OR “Laryngeal Abductor Paralysis”[tiab] OR “Laryngeal Abductor Paralysis-intellectual Disability Syndrome”[tiab] OR “Lathosterolosis”[tiab] OR “Laurence-moon Syndrome”[tiab] OR “Laurin-sandrow Syndrome”[tiab] OR “Lead Poisoning”[tiab] OR “Leber Congenital Amaurosis”[tiab] OR “Leber Congenital Amaurosis, Type I”[tiab] OR “Leber Congenital Amaurosis, Type Ii”[tiab] OR “Leber Optic Atrophy And Dystonia”[tiab] OR “Leigh Syndrome”[tiab] OR “Leigh Syndrome With Cardiomyopathy”[tiab] OR “Leigh Syndrome With Leukodystrophy”[tiab] OR “Lelis Syndrome”[tiab] OR “Lennox-gastaut Syndrome”[tiab] OR “Lentiginosis, Centrofacial Neurodysraphic”[tiab] OR “Lenz-majewski Hyperostotic Dwarfism”[tiab] OR “Leopard Syndrome 1”[tiab] OR “Leopard Syndrome 3”[tiab] OR “Leprechaunism”[tiab] OR “Lesch-nyhan Phenotype With Normal Hgprt”[tiab] OR “Lesch-nyhan Syndrome”[tiab] OR “Lessel-kreienkamp Syndrome”[tiab] OR “Lethal Ataxia With Deafness And Optic Atrophy”[tiab] OR “Leukocyte Adhesion Deficiency”[tiab] OR “Leukocyte Adhesion Deficiency Type Ii”[tiab] OR “Leukodystrophy And Acquired Microcephaly With Or Without Dystonia”[tiab] OR “Leukodystrophy, Hypomyelinating, 11”[tiab] OR “Leukodystrophy, Hypomyelinating, 12”[tiab] OR “Leukodystrophy, Hypomyelinating, 14”[tiab] OR “Leukodystrophy, Hypomyelinating, 16”[tiab] OR “Leukodystrophy, Hypomyelinating, 17”[tiab] OR “Leukodystrophy, Hypomyelinating, 22”[tiab] OR “Leukodystrophy, Hypomyelinating, 23, With Ataxia, Deafness, Liver Dysfunction, And Dilated Cardiomyopathy”[tiab] OR “Leukodystrophy, Hypomyelinating, 4”[tiab] OR “Leukodystrophy, Hypomyelinating, 5”[tiab] OR “Leukodystrophy, Hypomyelinating, 6”[tiab] OR “Leukodystrophy, Hypomyelinating, 8, With Or Without Oligodontia And/or Hypogonadotropic Hypogonadism”[tiab] OR “Leukodystrophy, Hypomyelinating, 9”[tiab] OR “Leukodystrophy, Progressive, Early Childhood-onset”[tiab] OR “Leukoencephalopathy With Bilateral Anterior Temporal Lobe Cysts”[tiab] OR “Leukoencephalopathy With Brain Stem And Spinal Cord Involvement-high Lactate Syndrome”[tiab] OR “Leukoencephalopathy With Metaphyseal Chondrodysplasia”[tiab] OR “Leukoencephalopathy-spondyloepimetaphyseal Dysplasia Syndrome”[tiab] OR “Leukomelanoderma, Infantilism, Mental Retardation, Hypodontia, Hypotrichosis”[tiab] OR “Leukomelanoderma-infantilism-intellectual Disability-hypodontia-hypotrichosis Syndrome”[tiab] OR “Li-campeau Syndrome”[tiab] OR “Liberfarb Syndrome”[tiab] OR “Lig4 Syndrome”[tiab] OR “Limb Defects, Distal Transverse, With Mental Retardation And Spasticity”[tiab] OR “Linear Nevus Sebaceus Syndrome”[tiab] OR “Lipodystrophy, Congenital Generalized, Type 2”[tiab] OR “Lipodystrophy, Generalized, With Mental Retardation, Deafness, Short Stature, And Slender Bones”[tiab] OR “Lipodystrophy-intellectual Disability-deafness Syndrome”[tiab] OR “Lissencephaly 1”[tiab] OR “Lissencephaly 10”[tiab] OR “Lissencephaly 3”[tiab] OR “Lissencephaly 4”[tiab] OR “Lissencephaly 5”[tiab] OR “Lissencephaly 8”[tiab] OR “Lissencephaly Due To Lis1 Mutation”[tiab] OR “Lissencephaly Syndrome, Norman-roberts Type”[tiab] OR “Lissencephaly, X-linked, 1”[tiab] OR “Listeriosis”[tiab] OR “Lobar Holoprosencephaly”[tiab] OR “Loeys-dietz Syndrome 1”[tiab] OR “Loeys-dietz Syndrome 2”[tiab] OR “Long Chain 3-hydroxyacyl-coa Dehydrogenase Deficiency”[tiab] OR “Long Qt Syndrome 8”[tiab] OR “Lopes-maciel-rodan Syndrome”[tiab] OR “Lowe Syndrome”[tiab] OR “Lowry-maclean Syndrome”[tiab] OR “Lowry-wood Syndrome”[tiab] OR “Lujan-fryns Syndrome”[tiab] OR “Luo-schoch-yamamoto Syndrome”[tiab] OR “Luscan-lumish Syndrome”[tiab] OR “Lysine Malabsorption Syndrome”[tiab] OR “Lysinuric Protein Intolerance”[tiab] |
| **M, N, O, P, Q, R** | “Macrocephaly And Epileptic Encephalopathy”[tiab] OR “Macrocephaly, Dysmorphic Facies, And Psychomotor Retardation”[tiab] OR “Macrocephaly, Neurodevelopmental Delay, Lymphoid Hyperplasia, And Persistent Fetal Hemoglobin”[tiab] OR “Macrocephaly-developmental Delay Syndrome”[tiab] OR “Macrocephaly-intellectual Disability-left Ventricular Non Compaction Syndrome”[tiab] OR “Macrocephaly-intellectual Disability-neurodevelopmental Disorder-small Thorax Syndrome”[tiab] OR “Macrocephaly-spastic Paraplegia-dysmorphism Syndrome”[tiab] OR “Macrocephaly/autism Syndrome”[tiab] OR “Macrocephaly/megalencephaly Syndrome, Autosomal Recessive”[tiab] OR “Macrothrombocytopenia-lymphedema-developmental Delay-facial Dysmorphism-camptodactyly Syndrome”[tiab] OR “Magel2-related Prader-willi-like Syndrome”[tiab] OR “Malan Overgrowth Syndrome”[tiab] OR “Malan Syndrome”[tiab] OR “Male Hypergonadotropic Hypogonadism-intellectual Disability-skeletal Anomalies Syndrome”[tiab] OR “Male Pseudohermaphroditism/mental Retardation Syndrome, Verloes Type”[tiab] OR “Malonyl-coa Decarboxylase Deficiency”[tiab] OR “Malouf Syndrome”[tiab] OR “Man1b1-cdg”[tiab] OR “Mandibulofacial Dysostosis With Mental Retardation”[tiab] OR “Mandibulofacial Dysostosis-microcephaly Syndrome”[tiab] OR “Mannosidosis, Beta A, Lysosomal”[tiab] OR “Maple Syrup Urine Disease”[tiab] OR “Marden-walker Syndrome”[tiab] OR “Marfanoid Habitus With Microcephaly And Glomerulonephritis”[tiab] OR “Marfanoid Mental Retardation Syndrome, Autosomal”[tiab] OR “Marinesco-sjogren Syndrome”[tiab] OR “Marinesco-sjögren Syndrome”[tiab] OR “Marshall-smith Syndrome”[tiab] OR “Martsolf Syndrome 1”[tiab] OR “Martsolf Syndrome 2”[tiab] OR “Masa Syndrome”[tiab] OR “Maternal Phenylketonuria”[tiab] OR “Maternal Uniparental Disomy Of Chromosome 4”[tiab] OR “Maternal Uniparental Disomy Of Chromosome 6”[tiab] OR “Maternal Uniparental Disomy Of Chromosome X”[tiab] OR “Matthew-wood Syndrome”[tiab] OR “Mcdonough Syndrome”[tiab] OR “Mckusick-kaufman Syndrome”[tiab] OR “Meckel Syndrome 13”[tiab] OR “Mednik Syndrome”[tiab] OR “Megalencephalic Leukoencephalopathy With Subcortical Cysts 1”[tiab] OR “Megalencephalic Leukoencephalopathy With Subcortical Cysts 2a”[tiab] OR “Megalencephalic Leukoencephalopathy With Subcortical Cysts 2b, Remitting, With Or Without Mental Retardation”[tiab] OR “Megalencephaly”[tiab] OR “Megalencephaly-capillary Malformation-polymicrogyria Syndrome”[tiab] OR “Megalencephaly-polymicrogyria-polydactyly-hydrocephalus Syndrome”[tiab] OR “Megalencephaly-polymicrogyria-polydactyly-hydrocephalus Syndrome 2”[tiab] OR “Megalencephaly-severe Kyphoscoliosis-overgrowth Syndrome”[tiab] OR “Megalocornea-intellectual Disability Syndrome”[tiab] OR “Megalocornea-mental Retardation Syndrome”[tiab] OR “Mehmo Syndrome”[tiab] OR “Meier-gorlin Syndrome 1”[tiab] OR “Meier-gorlin Syndrome 4”[tiab] OR “Meier-gorlin Syndrome 6”[tiab] OR “Mend Syndrome”[tiab] OR “Menke-hennekam Syndrome 1”[tiab] OR “Menke-hennekam Syndrome 2”[tiab] OR “Menkes Disease”[tiab] OR “Mental And Growth Retardation With Amblyopia”[tiab] OR “Mental Retardation And Distinctive Facial Features With Or Without Cardiac Defects”[tiab] OR “Mental Retardation And Microcephaly With Pontine And Cerebellar Hypoplasia”[tiab] OR “Mental Retardation Associated With Psoriasis”[tiab] OR “Mental Retardation Syndrome, Belgian Type”[tiab] OR “Mental Retardation Syndrome, Mietens-weber Type”[tiab] OR “Mental Retardation Syndrome, X-linked, Armfield Type”[tiab] OR “Mental Retardation With Language Impairment And With Or Without Autistic Features”[tiab] OR “Mental Retardation With Optic Atrophy, Deafness, And Seizures”[tiab] OR “Mental Retardation With Optic Atrophy, Facial Dysmorphism, Microcephaly,and Short Stature”[tiab] OR “Mental Retardation With Spastic Paraplegia”[tiab] OR “Mental Retardation With Spastic Paraplegia And Palmoplantar Hyperkeratosis”[tiab] OR “Mental Retardation, Anterior Maxillary Protrusion, And Strabismus”[tiab] OR “Mental Retardation, Autosomal Dominant 1”[tiab] OR “Mental Retardation, Autosomal Dominant 10”[tiab] OR “Mental Retardation, Autosomal Dominant 13”[tiab] OR “Mental Retardation, Autosomal Dominant 18”[tiab] OR “Mental Retardation, Autosomal Dominant 2”[tiab] OR “Mental Retardation, Autosomal Dominant 20”[tiab] OR “Mental Retardation, Autosomal Dominant 21”[tiab] OR “Mental Retardation, Autosomal Dominant 22”[tiab] OR “Mental Retardation, Autosomal Dominant 23”[tiab] OR “Mental Retardation, Autosomal Dominant 24”[tiab] OR “Mental Retardation, Autosomal Dominant 26”[tiab] OR “Mental Retardation, Autosomal Dominant 29”[tiab] OR “Mental Retardation, Autosomal Dominant 3”[tiab] OR “Mental Retardation, Autosomal Dominant 30”[tiab] OR “Mental Retardation, Autosomal Dominant 31”[tiab] OR “Mental Retardation, Autosomal Dominant 33”[tiab] OR “Mental Retardation, Autosomal Dominant 34”[tiab] OR “Mental Retardation, Autosomal Dominant 35”[tiab] OR “Mental Retardation, Autosomal Dominant 36”[tiab] OR “Mental Retardation, Autosomal Dominant 38”[tiab] OR “Mental Retardation, Autosomal Dominant 39”[tiab] OR “Mental Retardation, Autosomal Dominant 4”[tiab] OR “Mental Retardation, Autosomal Dominant 40”[tiab] OR “Mental Retardation, Autosomal Dominant 41”[tiab] OR “Mental Retardation, Autosomal Dominant 42”[tiab] OR “Mental Retardation, Autosomal Dominant 43”[tiab] OR “Mental Retardation, Autosomal Dominant 44”[tiab] OR “Mental Retardation, Autosomal Dominant 45”[tiab] OR “Mental Retardation, Autosomal Dominant 46”[tiab] OR “Mental Retardation, Autosomal Dominant 47”[tiab] OR “Mental Retardation, Autosomal Dominant 48”[tiab] OR “Mental Retardation, Autosomal Dominant 49”[tiab] OR “Mental Retardation, Autosomal Dominant 5”[tiab] OR “Mental Retardation, Autosomal Dominant 50”[tiab] OR “Mental Retardation, Autosomal Dominant 51”[tiab] OR “Mental Retardation, Autosomal Dominant 52”[tiab] OR “Mental Retardation, Autosomal Dominant 53”[tiab] OR “Mental Retardation, Autosomal Dominant 54”[tiab] OR “Mental Retardation, Autosomal Dominant 55, With Seizures”[tiab] OR “Mental Retardation, Autosomal Dominant 56”[tiab] OR “Mental Retardation, Autosomal Dominant 57”[tiab] OR “Mental Retardation, Autosomal Dominant 58”[tiab] OR “Mental Retardation, Autosomal Dominant 6, With Or Without Seizures”[tiab] OR “Mental Retardation, Autosomal Dominant 7”[tiab] OR “Mental Retardation, Autosomal Dominant 9”[tiab] OR “Mental Retardation, Autosomal Recessive 1”[tiab] OR “Mental Retardation, Autosomal Recessive 10”[tiab] OR “Mental Retardation, Autosomal Recessive 11”[tiab] OR “Mental Retardation, Autosomal Recessive 12”[tiab] OR “Mental Retardation, Autosomal Recessive 13”[tiab] OR “Mental Retardation, Autosomal Recessive 14”[tiab] OR “Mental Retardation, Autosomal Recessive 16”[tiab] OR “Mental Retardation, Autosomal Recessive 18”[tiab] OR “Mental Retardation, Autosomal Recessive 19”[tiab] OR “Mental Retardation, Autosomal Recessive 2”[tiab] OR “Mental Retardation, Autosomal Recessive 23”[tiab] OR “Mental Retardation, Autosomal Recessive 24”[tiab] OR “Mental Retardation, Autosomal Recessive 25”[tiab] OR “Mental Retardation, Autosomal Recessive 27”[tiab] OR “Mental Retardation, Autosomal Recessive 28”[tiab] OR “Mental Retardation, Autosomal Recessive 29”[tiab] OR “Mental Retardation, Autosomal Recessive 3”[tiab] OR “Mental Retardation, Autosomal Recessive 30”[tiab] OR “Mental Retardation, Autosomal Recessive 31”[tiab] OR “Mental Retardation, Autosomal Recessive 33”[tiab] OR “Mental Retardation, Autosomal Recessive 34, With Variant Lissencephaly”[tiab] OR “Mental Retardation, Autosomal Recessive 35”[tiab] OR “Mental Retardation, Autosomal Recessive 36”[tiab] OR “Mental Retardation, Autosomal Recessive 37”[tiab] OR “Mental Retardation, Autosomal Recessive 38”[tiab] OR “Mental Retardation, Autosomal Recessive 39”[tiab] OR “Mental Retardation, Autosomal Recessive 41”[tiab] OR “Mental Retardation, Autosomal Recessive 43”[tiab] OR “Mental Retardation, Autosomal Recessive 44”[tiab] OR “Mental Retardation, Autosomal Recessive 45”[tiab] OR “Mental Retardation, Autosomal Recessive 46”[tiab] OR “Mental Retardation, Autosomal Recessive 47”[tiab] OR “Mental Retardation, Autosomal Recessive 48”[tiab] OR “Mental Retardation, Autosomal Recessive 49”[tiab] OR “Mental Retardation, Autosomal Recessive 5”[tiab] OR “Mental Retardation, Autosomal Recessive 50”[tiab] OR “Mental Retardation, Autosomal Recessive 51”[tiab] OR “Mental Retardation, Autosomal Recessive 52”[tiab] OR “Mental Retardation, Autosomal Recessive 53”[tiab] OR “Mental Retardation, Autosomal Recessive 54”[tiab] OR “Mental Retardation, Autosomal Recessive 56”[tiab] OR “Mental Retardation, Autosomal Recessive 57”[tiab] OR “Mental Retardation, Autosomal Recessive 58”[tiab] OR “Mental Retardation, Autosomal Recessive 59”[tiab] OR “Mental Retardation, Autosomal Recessive 6”[tiab] OR “Mental Retardation, Autosomal Recessive 60”[tiab] OR “Mental Retardation, Autosomal Recessive 61”[tiab] OR “Mental Retardation, Autosomal Recessive 63”[tiab] OR “Mental Retardation, Autosomal Recessive 64”[tiab] OR “Mental Retardation, Autosomal Recessive 65”[tiab] OR “Mental Retardation, Autosomal Recessive 66”[tiab] OR “Mental Retardation, Autosomal Recessive 7”[tiab] OR “Mental Retardation, Autosomal Recessive 9”[tiab] OR “Mental Retardation, Autosomal Recessive, 4”[tiab] OR “Mental Retardation, Buenos Aires Type”[tiab] OR “Mental Retardation, Enteropathy, Deafness, Peripheral Neuropathy, Ichthyosis, And Keratoderma”[tiab] OR “Mental Retardation, Fra12a Type”[tiab] OR “Mental Retardation, Keratoconus, Febrile Seizures, And Sinoatrialblock”[tiab] OR “Mental Retardation, Microcephaly, Epilepsy, And Coarse Face”[tiab] OR “Mental Retardation, Microcephaly, Growth Retardation, Joint Contractures,and Facial Dysmorphism”[tiab] OR “Mental Retardation, Obesity, Mandibular Prognathism, And Eye And Skinanomalies”[tiab] OR “Mental Retardation, Skeletal Dysplasia, And Abducens Palsy”[tiab] OR “Mental Retardation, Truncal Obesity, Retinal Dystrophy, And Micropenis Syndrome”[tiab] OR “Mental Retardation, X-linked 1”[tiab] OR “Mental Retardation, X-linked 100”[tiab] OR “Mental Retardation, X-linked 101”[tiab] OR “Mental Retardation, X-linked 103”[tiab] OR “Mental Retardation, X-linked 104”[tiab] OR “Mental Retardation, X-linked 105”[tiab] OR “Mental Retardation, X-linked 106”[tiab] OR “Mental Retardation, X-linked 107”[tiab] OR “Mental Retardation, X-linked 12/35”[tiab] OR “Mental Retardation, X-linked 14”[tiab] OR “Mental Retardation, X-linked 19”[tiab] OR “Mental Retardation, X-linked 2”[tiab] OR “Mental Retardation, X-linked 20”[tiab] OR “Mental Retardation, X-linked 21”[tiab] OR “Mental Retardation, X-linked 23”[tiab] OR “Mental Retardation, X-linked 30”[tiab] OR “Mental Retardation, X-linked 41”[tiab] OR “Mental Retardation, X-linked 45”[tiab] OR “Mental Retardation, X-linked 46”[tiab] OR “Mental Retardation, X-linked 50”[tiab] OR “Mental Retardation, X-linked 53”[tiab] OR “Mental Retardation, X-linked 58”[tiab] OR “Mental Retardation, X-linked 63”[tiab] OR “Mental Retardation, X-linked 72”[tiab] OR “Mental Retardation, X-linked 73”[tiab] OR “Mental Retardation, X-linked 77”[tiab] OR “Mental Retardation, X-linked 81”[tiab] OR “Mental Retardation, X-linked 82”[tiab] OR “Mental Retardation, X-linked 84”[tiab] OR “Mental Retardation, X-linked 88”[tiab] OR “Mental Retardation, X-linked 89”[tiab] OR “Mental Retardation, X-linked 9”[tiab] OR “Mental Retardation, X-linked 90”[tiab] OR “Mental Retardation, X-linked 91”[tiab] OR “Mental Retardation, X-linked 92”[tiab] OR “Mental Retardation, X-linked 93”[tiab] OR “Mental Retardation, X-linked 94”[tiab] OR “Mental Retardation, X-linked 95”[tiab] OR “Mental Retardation, X-linked 96”[tiab] OR “Mental Retardation, X-linked 97”[tiab] OR “Mental Retardation, X-linked 98”[tiab] OR “Mental Retardation, X-linked 99”[tiab] OR “Mental Retardation, X-linked 99, Syndromic, Female-restricted”[tiab] OR “Mental Retardation, X-linked Syndromic, Christianson Type”[tiab] OR “Mental Retardation, X-linked Syndromic, Lubs Type”[tiab] OR “Mental Retardation, X-linked Syndromic, Raymond Type”[tiab] OR “Mental Retardation, X-linked Syndromic, Turner Type”[tiab] OR “Mental Retardation, X-linked, Associated With Fragile Site Fraxe”[tiab] OR “Mental Retardation, X-linked, Syndromic 11”[tiab] OR “Mental Retardation, X-linked, Syndromic 12”[tiab] OR “Mental Retardation, X-linked, Syndromic 13”[tiab] OR “Mental Retardation, X-linked, Syndromic 14”[tiab] OR “Mental Retardation, X-linked, Syndromic 17”[tiab] OR “Mental Retardation, X-linked, Syndromic 32”[tiab] OR “Mental Retardation, X-linked, Syndromic 33”[tiab] OR “Mental Retardation, X-linked, Syndromic 34”[tiab] OR “Mental Retardation, X-linked, Syndromic, 35”[tiab] OR “Mental Retardation, X-linked, Syndromic, Bain Type”[tiab] OR “Mental Retardation, X-linked, Syndromic, Cabezas Type”[tiab] OR “Mental Retardation, X-linked, Syndromic, Chudley-schwartz Type”[tiab] OR “Mental Retardation, X-linked, Syndromic, Claes-jensen Type”[tiab] OR “Mental Retardation, X-linked, Syndromic, Hedera Type”[tiab] OR “Mental Retardation, X-linked, Syndromic, Martin-probst Type”[tiab] OR “Mental Retardation, X-linked, Syndromic, Nascimento Type”[tiab] OR “Mental Retardation, X-linked, Syndromic, Snyder-robinson Type”[tiab] OR “Mental Retardation, X-linked, Syndromic, Turner Type”[tiab] OR “Mental Retardation, X-linked, With Cerebellar Hypoplasia And Distinctive Facial Appearance”[tiab] OR “Mental Retardation, X-linked, With Craniofacial Dysmorphism”[tiab] OR “Mental Retardation, X-linked, With Isolated Growth Hormone Deficiency”[tiab] OR “Mental Retardation, X-linked, With Or Without Seizures, Arx-related”[tiab] OR “Mental Retardation-hypotonic Facies Syndrome, X-linked, 1”[tiab] OR “Mercaptolactate-cysteine Disulfiduria”[tiab] OR “Mesangial Sclerosis, Diffuse Renal, With Ocular Abnormalities”[tiab] OR “Mesoaxial Hexadactyly And Cardiac Malformation”[tiab] OR “Mesomelic Dysplasia, Nievergelt Type”[tiab] OR “Mesomelic Dysplasia, Savarirayan Type”[tiab] OR “Metabolic Encephalomyopathic Crises, Recurrent, With Rhabdomyolysis, Cardiac Arrhythmias, And Neurodegeneration”[tiab] OR “Metachromatic Leukodystrophy”[tiab] OR “Metaphyseal Acroscyphodysplasia”[tiab] OR “Metaphyseal Dysostosis, Mental Retardation, And Conductive Deafness”[tiab] OR “Metaphyseal Dysostosis-intellectual Disability-conductive Deafness Syndrome”[tiab] OR “Methemoglobinemia Due To Deficiency Of Methemoglobin Reductase”[tiab] OR “Methionine Adenosyltransferase I/iii Deficiency”[tiab] OR “Methionine Malabsorption Syndrome”[tiab] OR “Methylcobalamin Deficiency Type Cble”[tiab] OR “Methylmalonic Acidemia And Homocysteinemia, Cblx Type”[tiab] OR “Methylmalonic Acidemia With Homocystinuria”[tiab] OR “Methylmalonic Acidemia With Homocystinuria Type Cblf”[tiab] OR “Methylmalonic Acidemia With Homocystinuria, Type Cblc”[tiab] OR “Methylmalonic Acidemia With Homocystinuria, Type Cbld”[tiab] OR “Methylmalonic Aciduria And Homocystinuria, Cblc Type”[tiab] OR “Methylmalonic Aciduria And Homocystinuria, Cbld Type”[tiab] OR “Mevalonic Aciduria”[tiab] OR “Micro Syndrome”[tiab] OR “Microbrachycephaly-ptosis-cleft Lip Syndrome”[tiab] OR “Microcephalic Cortical Malformations-short Stature Due To Rttn Deficiency”[tiab] OR “Microcephalic Osteodysplastic Primordial Dwarfism Type Ii”[tiab] OR “Microcephalic Osteodysplastic Primordial Dwarfism Types I And Iii”[tiab] OR “Microcephalic Osteodysplastic Primordial Dwarfism, Type I”[tiab] OR “Microcephalic Osteodysplastic Primordial Dwarfism, Type Ii”[tiab] OR “Microcephalic Osteodysplastic Primordial Dwarfism, Type Iii”[tiab] OR “Microcephalic Primordial Dwarfism, Dauber Type”[tiab] OR “Microcephalic Primordial Dwarfism, Montreal Type”[tiab] OR “Microcephalic Primordial Dwarfism, Toriello Type”[tiab] OR “Microcephaly 12, Primary, Autosomal Recessive”[tiab] OR “Microcephaly 14, Primary, Autosomal Recessive”[tiab] OR “Microcephaly 15, Primary, Autosomal Recessive”[tiab] OR “Microcephaly 17, Primary, Autosomal Recessive”[tiab] OR “Microcephaly 18, Primary, Autosomal Dominant”[tiab] OR “Microcephaly 2, Primary, Autosomal Recessive, With Or Without Cortical Malformations”[tiab] OR “Microcephaly 20, Primary, Autosomal Recessive”[tiab] OR “Microcephaly 21, Primary, Autosomal Recessive”[tiab] OR “Microcephaly 23, Primary, Autosomal Recessive”[tiab] OR “Microcephaly 28, Primary, Autosomal Recessive”[tiab] OR “Microcephaly 3, Primary, Autosomal Recessive”[tiab] OR “Microcephaly 4, Primary, Autosomal Recessive”[tiab] OR “Microcephaly 5, Primary, Autosomal Recessive”[tiab] OR “Microcephaly 7, Primary, Autosomal Recessive”[tiab] OR “Microcephaly 8, Primary, Autosomal Recessive”[tiab] OR “Microcephaly And Chorioretinopathy, Autosomal Recessive, 1”[tiab] OR “Microcephaly And Chorioretinopathy, Autosomal Recessive, 2”[tiab] OR “Microcephaly With Cervical Spine Fusion Anomalies”[tiab] OR “Microcephaly With Or Without Chorioretinopathy, Lymphedema, Or Mental Retardation”[tiab] OR “Microcephaly, Cerebellar Hypoplasia, And Cardiac Conduction Defect Syndrome”[tiab] OR “Microcephaly, Congenital Cataract, And Psoriasiform Dermatitis”[tiab] OR “Microcephaly, Developmental Delay, And Brittle Hair Syndrome”[tiab] OR “Microcephaly, Epilepsy, And Diabetes Syndrome”[tiab] OR “Microcephaly, Primary Autosomal Recessive, 1”[tiab] OR “Microcephaly, Primary Autosomal Recessive, 6”[tiab] OR “Microcephaly, Seizures, And Developmental Delay”[tiab] OR “Microcephaly, Seizures, Spasticity, And Brain Calcifications”[tiab] OR “Microcephaly, Short Stature, And Impaired Glucose Metabolism 1”[tiab] OR “Microcephaly, Short Stature, And Impaired Glucose Metabolism 2”[tiab] OR “Microcephaly, Short Stature, And Limb Abnormalities”[tiab] OR “Microcephaly, Short Stature, And Polymicrogyria With Or Without Seizures”[tiab] OR “Microcephaly-brachydactyly-kyphoscoliosis Syndrome”[tiab] OR “Microcephaly-cardiomyopathy”[tiab] OR “Microcephaly-cardiomyopathy Syndrome”[tiab] OR “Microcephaly-cervical Spine Fusion Anomalies Syndrome”[tiab] OR “Microcephaly-cleft Palate-abnormal Retinal Pigmentation Syndrome”[tiab] OR “Microcephaly-corpus Callosum And Cerebellar Vermis Hypoplasia-facial Dysmorphism-intellectual Disability Syndrom”[tiab] OR “Microcephaly-corpus Callosum Hypoplasia-intellectual Disability-facial Dysmorphism Syndrome”[tiab] OR “Microcephaly-deafness Syndrome”[tiab] OR “Microcephaly-deafness-intellectual Disability Syndrome”[tiab] OR “Microcephaly-glomerulonephritis-marfanoid Habitus Syndrome”[tiab] OR “Microcephaly-intellectual Disability-sensorineural Hearing Loss-epilepsy-abnormal Muscle Tone Syndrome”[tiab] OR “Microcephaly-lymphedema-chorioretinopathy Syndrome”[tiab] OR “Microcephaly-microcornea Syndrome, Seemanova Type”[tiab] OR “Microcephaly-seizures-intellectual Disability-heart Disease Syndrome”[tiab] OR “Microcephaly-thin Corpus Callosum-intellectual Disability Syndrome”[tiab] OR “Microduplication Xp11.22p11.23 Syndrome”[tiab] OR “Microform Holoprosencephaly”[tiab] OR “Micrognathia-recurrent Infections-behavioral Abnormalities-mild Intellectual Disability Syndrome”[tiab] OR “Microhydranencephaly”[tiab] OR “Microlissencephaly”[tiab] OR “Microphthalmia With Limb Anomalies”[tiab] OR “Microphthalmia With Linear Skin Defects Syndrome”[tiab] OR “Microphthalmia, Isolated, With Coloboma 9”[tiab] OR “Microphthalmia, Lenz Type”[tiab] OR “Microphthalmia, Syndromic 1”[tiab] OR “Microphthalmia, Syndromic 12”[tiab] OR “Microphthalmia, Syndromic 13”[tiab] OR “Microphthalmia, Syndromic 2”[tiab] OR “Microphthalmia, Syndromic 7”[tiab] OR “Microphthalmia, Syndromic 8”[tiab] OR “Microphthalmia, Syndromic 9”[tiab] OR “Microphthalmia-ankyloblepharon-intellectual Disability Syndrome”[tiab] OR “Microphthalmia/coloboma And Skeletal Dysplasia Syndrome”[tiab] OR “Microtriplication 11q24.1”[tiab] OR “Midface Hypoplasia, Hearing Impairment, Elliptocytosis, And Nephrocalcinosis”[tiab] OR “Midline Interhemispheric Variant Of Holoprosencephaly”[tiab] OR “Mietens Syndrome”[tiab] OR “Migraine, Familial Hemiplegic, 2”[tiab] OR “Miller-dieker Lissencephaly Syndrome”[tiab] OR “Mirror Movements 1”[tiab] OR “Mitochondrial Complex I Deficiency, Nuclear Type 16”[tiab] OR “Mitochondrial Complex Iii Deficiency, Nuclear Type 1”[tiab] OR “Mitochondrial Complex Iii Deficiency, Nuclear Type 4”[tiab] OR “Mitochondrial Complex Iii Deficiency, Nuclear Type 8”[tiab] OR “Mitochondrial Complex Iv Deficiency”[tiab] OR “Mitochondrial Complex Iv Deficiency, Nuclear Type 17”[tiab] OR “Mitochondrial Complex Iv Deficiency, Nuclear Type 8”[tiab] OR “Mitochondrial Complex V (atp Synthase) Deficiency, Nuclear Type 3”[tiab] OR “Mitochondrial Dna Depletion Syndrome 11”[tiab] OR “Mitochondrial Dna Depletion Syndrome 12 (cardiomyopathic Type)”[tiab] OR “Mitochondrial Dna Depletion Syndrome 2 (myopathic Type)”[tiab] OR “Mitochondrial Dna Depletion Syndrome 5 (encephalomyopathic With Or Without Methylmalonic Aciduria)”[tiab] OR “Mitochondrial Dna Depletion Syndrome 7 (hepatocerebral Type)”[tiab] OR “Mitochondrial Dna Depletion Syndrome 8a (encephalomyopathic Type With Renal Tubulopathy)”[tiab] OR “Mitochondrial Dna Depletion Syndrome 9 (encephalomyopathic Type With Methylmalonic Aciduria)”[tiab] OR “Mitochondrial Dna Depletion Syndrome, Encephalomyopathic Form With Methylmalonic Aciduria”[tiab] OR “Mitochondrial Dna-related Progressive External Ophthalmoplegia”[tiab] OR “Mitochondrial Myopathy And Sideroblastic Anemia”[tiab] OR “Mitochondrial Myopathy-cerebellar Ataxia-pigmentary Retinopathy Syndrome”[tiab] OR “Mitochondrial Neurogastrointestinal Encephalomyopathy”[tiab] OR “Mmep Syndrome”[tiab] OR “Moebius Syndrome”[tiab] OR “Moebius Syndrome-axonal Neuropathy-hypogonadotropic Hypogonadism Syndrome”[tiab] OR “Molybdenum Cofactor Deficiency, Complementation Group A”[tiab] OR “Momo Syndrome”[tiab] OR “Monilethrix”[tiab] OR “Monocarboxylate Transporter 1 Deficiency”[tiab] OR “Monosomy 13q14”[tiab] OR “Monosomy 13q34”[tiab] OR “Monosomy 18p”[tiab] OR “Monosomy 18q”[tiab] OR “Monosomy 22”[tiab] OR “Monosomy 22q13.3”[tiab] OR “Monosomy 5p”[tiab] OR “Monosomy 9p”[tiab] OR “Monosomy 9q22.3”[tiab] OR “Morbid Obesity And Spermatogenic Failure”[tiab] OR “Morm Syndrome”[tiab] OR “Morquio Syndrome C”[tiab] OR “Mosaic Trisomy 1”[tiab] OR “Mosaic Trisomy 14”[tiab] OR “Mosaic Trisomy 8”[tiab] OR “Mosaic Trisomy 9”[tiab] OR “Mosaic Variegated Aneuploidy Syndrome”[tiab] OR “Mosaic Variegated Aneuploidy Syndrome 1”[tiab] OR “Mosaic Variegated Aneuploidy Syndrome 2”[tiab] OR “Mowat-wilson Syndrome”[tiab] OR “Mowat-wilson Syndrome Due To A Zeb2 Point Mutation”[tiab] OR “Mowat-wilson Syndrome Due To Monosomy 2q22”[tiab] OR “Moyamoya Disease”[tiab] OR “Moynahan Syndrome”[tiab] OR “Mpdu1-cdg”[tiab] OR “Mucolipidosis Iii Alpha/beta”[tiab] OR “Mucolipidosis Iii Gamma”[tiab] OR “Mucolipidosis Iv”[tiab] OR “Mucolipidosis Type Iv”[tiab] OR “Mucopolysaccharidosis Type 1”[tiab] OR “Mucopolysaccharidosis Type 3”[tiab] OR “Mucopolysaccharidosis Type 7”[tiab] OR “Mucopolysaccharidosis Type Iiia”[tiab] OR “Mucopolysaccharidosis Type Iiib”[tiab] OR “Mucopolysaccharidosis Type Iiic”[tiab] OR “Mucopolysaccharidosis Vii”[tiab] OR “Mucopolysaccharidosis, Type Ii”[tiab] OR “Mucopolysaccharidosis, Type Iiid”[tiab] OR “Muenke Syndrome”[tiab] OR “Mulibrey Nanism”[tiab] OR “Multicentric Osteolysis-nodulosis-arthropathy Spectrum”[tiab] OR “Multicore Myopathy With Mental Retardation, Short Stature, And Hypogonadotropichypogonadism”[tiab] OR “Multiple Benign Circumferential Skin Creases On Limbs”[tiab] OR “Multiple Congenital Anomalies-hypotonia-seizures Syndrome 1”[tiab] OR “Multiple Congenital Anomalies-neurodevelopmental Syndrome, X-linked”[tiab] OR “Multiple Epiphyseal Dysplasia, Al-gazali Type”[tiab] OR “Multiple Mitochondrial Dysfunctions Syndrome 6”[tiab] OR “Multiple Sulfatase Deficiency”[tiab] OR “Muscle-eye-brain Disease With Bilateral Multicystic Leucodystrophy”[tiab] OR “Muscular Dystrophy, Congenital, Due To Integrin Alpha-7 Deficiency”[tiab] OR “Muscular Dystrophy, Congenital, Megaconial Type”[tiab] OR “Muscular Dystrophy, Congenital, Merosin Deficient Or Partially Deficient”[tiab] OR “Muscular Dystrophy, Congenital, With Cataracts And Intellectual Disability”[tiab] OR “Muscular Dystrophy, Limb-girdle, Autosomal Recessive 18”[tiab] OR “Muscular Dystrophy, Limb-girdle, Autosomal Recessive 27”[tiab] OR “Muscular Dystrophy-dystroglycanopathy (congenital With Brain And Eye Anomalies), Type A, 1”[tiab] OR “Muscular Dystrophy-dystroglycanopathy (congenital With Brain And Eye Anomalies), Type A, 14”[tiab] OR “Muscular Dystrophy-dystroglycanopathy (congenital With Brain And Eye Anomalies), Type A, 2”[tiab] OR “Muscular Dystrophy-dystroglycanopathy (congenital With Brain And Eye Anomalies), Type A, 3”[tiab] OR “Muscular Dystrophy-dystroglycanopathy (congenital With Brain And Eye Anomalies), Type A, 4”[tiab] OR “Muscular Dystrophy-dystroglycanopathy (congenital With Brain And Eye Anomalies), Type A, 5”[tiab] OR “Muscular Dystrophy-dystroglycanopathy (congenital With Brain And Eye Anomalies), Type A, 7”[tiab] OR “Muscular Dystrophy-dystroglycanopathy (congenital With Brain And Eyeanomalies), Type A, 6”[tiab] OR “Muscular Dystrophy-dystroglycanopathy (congenital With Mental Retardation), Type B, 1”[tiab] OR “Muscular Dystrophy-dystroglycanopathy (congenital With Mental Retardation), Type B, 14”[tiab] OR “Muscular Dystrophy-dystroglycanopathy (congenital With Mental Retardation), Type B, 2”[tiab] OR “Muscular Dystrophy-dystroglycanopathy (congenital With Mental Retardation), Type B, 3”[tiab] OR “Muscular Dystrophy-dystroglycanopathy (congenital With Mental Retardation), Type B, 6”[tiab] OR “Muscular Dystrophy-dystroglycanopathy (congenital With Or Withoutmental Retardation), Type B, 5”[tiab] OR “Muscular Dystrophy-dystroglycanopathy (limb-girdle), Type C, 1”[tiab] OR “Muscular Dystrophy-dystroglycanopathy (limb-girdle), Type C, 14”[tiab] OR “Muscular Dystrophy-dystroglycanopathy (limb-girdle), Type C, 9”[tiab] OR “Mycophenolate Mofetil Embryopathy”[tiab] OR “Myh7-related Late-onset Scapuloperoneal Muscular Dystrophy”[tiab] OR “Myhre Syndrome”[tiab] OR “Myoclonic Epilepsy Of Infancy”[tiab] OR “Myoclonic-astatic Epilepsy”[tiab] OR “Myoclonic-atonic Epilepsy”[tiab] OR “Myopathy And Diabetes Mellitus”[tiab] OR “Myopathy, Centronuclear, 2”[tiab] OR “Myopathy, Congenital, Bailey-bloch”[tiab] OR “Myopathy, Lactic Acidosis, And Sideroblastic Anemia 1”[tiab] OR “Myopathy, Mitochondrial, And Ataxia”[tiab] OR “Myotonia Permanens”[tiab] OR “Myotonia With Skeletal Abnormalities And Mental Retardation”[tiab] OR “Myotonic Dystrophy 1”[tiab] OR “N Syndrome”[tiab] OR “Nabais Sa-de Vries Syndrome, Type 1”[tiab] OR “Nabais Sa-de Vries Syndrome, Type 2”[tiab] OR “Nance-horan Syndrome”[tiab] OR “Narp Syndrome”[tiab] OR “Native American Myopathy”[tiab] OR “Nephrogenic Diabetes Insipidus-intracranial Calcification-facial Dysmorphism Syndrome”[tiab] OR “Nephronophthisis 18”[tiab] OR “Nephronophthisis-like Nephropathy 1”[tiab] OR “Nephrosialidosis”[tiab] OR “Nephrotic Syndrome, Type 8”[tiab] OR “Netherton Syndrome”[tiab] OR “Neuraminidase Deficiency”[tiab] OR “Neurocutaneous Melanocytosis”[tiab] OR “Neurodegeneration Due To Cerebral Folate Transport Deficiency”[tiab] OR “Neurodegeneration With Brain Iron Accumulation 2a”[tiab] OR “Neurodegeneration With Brain Iron Accumulation 5”[tiab] OR “Neurodegeneration, Childhood-onset, With Brain Atrophy”[tiab] OR “Neurodevelopmental Delay-seizures-ophthalmic Anomalies-osteopenia-cerebellar Atrophy Syndrome”[tiab] OR “Neurodevelopmental Disorder With Absent Language And Variable Seizures”[tiab] OR “Neurodevelopmental Disorder With Ataxia, Hypotonia, And Microcephaly”[tiab] OR “Neurodevelopmental Disorder With Behavioral Abnormalities, Absent Speech, And Hypotonia”[tiab] OR “Neurodevelopmental Disorder With Brain Anomalies, Seizures, And Scoliosis”[tiab] OR “Neurodevelopmental Disorder With Cardiomyopathy, Spasticity, And Brain Abnormalities”[tiab] OR “Neurodevelopmental Disorder With Cerebellar Atrophy And With Or Without Seizures”[tiab] OR “Neurodevelopmental Disorder With Cerebral Atrophy And Variable Facial Dysmorphism”[tiab] OR “Neurodevelopmental Disorder With Dysmorphic Facies And Distal Limb Anomalies”[tiab] OR “Neurodevelopmental Disorder With Dysmorphic Facies And Distal Skeletal Anomalies”[tiab] OR “Neurodevelopmental Disorder With Dysmorphic Facies And Thin Corpus Callosum”[tiab] OR “Neurodevelopmental Disorder With Dysmorphic Facies And Variable Seizures”[tiab] OR “Neurodevelopmental Disorder With Dysmorphic Facies, Impaired Speech And Hypotonia”[tiab] OR “Neurodevelopmental Disorder With Dysmorphic Features, Spasticity, And Brain Abnormalities”[tiab] OR “Neurodevelopmental Disorder With Epilepsy, Cataracts, Feeding Difficulties, And Delayed Brain Myelination”[tiab] OR “Neurodevelopmental Disorder With Epilepsy, Spasticity, And Brain Atrophy”[tiab] OR “Neurodevelopmental Disorder With Hearing Loss And Spasticity”[tiab] OR “Neurodevelopmental Disorder With Hyperkinetic Movements And Dyskinesia”[tiab] OR “Neurodevelopmental Disorder With Hypotonia And Autistic Features With Or Without Hyperkinetic Movements”[tiab] OR “Neurodevelopmental Disorder With Hypotonia And Brain Abnormalities”[tiab] OR “Neurodevelopmental Disorder With Hypotonia And Dysmorphic Facies”[tiab] OR “Neurodevelopmental Disorder With Hypotonia And Gross Motor And Seech Delay”[tiab] OR “Neurodevelopmental Disorder With Hypotonia, Microcephaly, And Seizures”[tiab] OR “Neurodevelopmental Disorder With Hypotonia, Neuropathy, And Deafness”[tiab] OR “Neurodevelopmental Disorder With Hypotonia, Seizures, And Absent Language”[tiab] OR “Neurodevelopmental Disorder With Impaired Language And Ataxia And With Or Without Seizures”[tiab] OR “Neurodevelopmental Disorder With Infantile Epileptic Spasms”[tiab] OR “Neurodevelopmental Disorder With Involuntary Movements”[tiab] OR “Neurodevelopmental Disorder With Language Impairment And Behavioral Abnormalities”[tiab] OR “Neurodevelopmental Disorder With Microcephaly And Gray Sclerae”[tiab] OR “Neurodevelopmental Disorder With Microcephaly And Structural Brain Anomalies”[tiab] OR “Neurodevelopmental Disorder With Microcephaly, Ataxia, And Seizures”[tiab] OR “Neurodevelopmental Disorder With Microcephaly, Cortical Malformations, And Spasticity”[tiab] OR “Neurodevelopmental Disorder With Microcephaly, Epilepsy, And Brain Atrophy”[tiab] OR “Neurodevelopmental Disorder With Microcephaly, Impaired Language, And Gait Abnormalities, Autosomal Recessive”[tiab] OR “Neurodevelopmental Disorder With Microcephaly, Impaired Language, Epilepsy, And Gait Abnormalities, Autosomal Dominant”[tiab] OR “Neurodevelopmental Disorder With Microcephaly, Seizures, And Cortical Atrophy”[tiab] OR “Neurodevelopmental Disorder With Midbrain And Hindbrain Malformations”[tiab] OR “Neurodevelopmental Disorder With Movement Abnormalities, Abnormal Gait, And Autistic Features”[tiab] OR “Neurodevelopmental Disorder With Nonspecific Brain Abnormalities And With Or Without Seizures”[tiab] OR “Neurodevelopmental Disorder With Or Without Autism Or Seizures”[tiab] OR “Neurodevelopmental Disorder With Or Without Autistic Features And/or Structural Brain Abnormalities”[tiab] OR “Neurodevelopmental Disorder With Or Without Hyperkinetic Movements And Seizures, Autosomal Dominant”[tiab] OR “Neurodevelopmental Disorder With Or Without Hyperkinetic Movements And Seizures, Autosomal Recessive”[tiab] OR “Neurodevelopmental Disorder With Or Without Seizures And Gait Abnormalities”[tiab] OR “Neurodevelopmental Disorder With Or Without Variable Movement Or Behavioral Abnormalities”[tiab] OR “Neurodevelopmental Disorder With Poor Language And Loss Of Hand Skills”[tiab] OR “Neurodevelopmental Disorder With Progressive Microcephaly, Spasticity, And Brain Anomalies”[tiab] OR “Neurodevelopmental Disorder With Progressive Spasticity And Brain White Matter Abnormalities”[tiab] OR “Neurodevelopmental Disorder With Regression, Abnormal Movements, Loss Of Speech, And Seizures”[tiab] OR “Neurodevelopmental Disorder With Seizures And Gingival Overgrowth”[tiab] OR “Neurodevelopmental Disorder With Seizures, Hypotonia, And Brain Imaging Abnormalities”[tiab] OR “Neurodevelopmental Disorder With Severe Motor Impairment And Absent Language”[tiab] OR “Neurodevelopmental Disorder With Spastic Diplegia And Visual Defects”[tiab] OR “Neurodevelopmental Disorder With Spastic Quadriplegia And Brain Abnormalities With Or Without Seizures”[tiab] OR “Neurodevelopmental Disorder With Spasticity And Poor Growth”[tiab] OR “Neurodevelopmental Disorder With Spasticity, Cataracts, And Cerebellar Hypoplasia”[tiab] OR “Neurodevelopmental Disorder With Speech Impairment And Dysmorphic Facies”[tiab] OR “Neurodevelopmental Disorder With Structural B[tiab]” |
| **S, T, U, V, W, X, Y, Z** | “Sabinas Brittle Hair Syndrome”[tiab] OR “Saccharopinuria”[tiab] OR “Saethre-chotzen Syndrome”[tiab] OR “Salla Disease”[tiab] OR “Sanjad-sakati Syndrome”[tiab] OR “Sarcosinemia”[tiab] OR “Satb2-associated Syndrome Due To A Chromosomal Rearrangement”[tiab] OR “Satb2-associated Syndrome Due To A Pathogenic Variant”[tiab] OR “Scarf Syndrome”[tiab] OR “Schaaf-yang Syndrome”[tiab] OR “Schilbach-rott Syndrome”[tiab] OR “Schimke X-linked Mental Retardation Syndrome”[tiab] OR “Schimmelpenning-feuerstein-mims Syndrome”[tiab] OR “Schindler Disease, Type I”[tiab] OR “Schinzel-giedion Midface-retraction Syndrome”[tiab] OR “Schizencephaly”[tiab] OR “Scholte Syndrome”[tiab] OR “Schuurs-hoeijmakers Syndrome”[tiab] OR “Schwartz-jampel Syndrome”[tiab] OR “Schwartz-jampel Syndrome, Type 1”[tiab] OR “Seckel Syndrome”[tiab] OR “Seckel Syndrome 1”[tiab] OR “Seckel Syndrome 5”[tiab] OR “Seckel Syndrome 6”[tiab] OR “Seckel Syndrome 7”[tiab] OR “Seckel Syndrome 8”[tiab] OR “Seizures, Sensorineural Deafness, Ataxia, Mental Retardation, And Electrolyte Imbalance”[tiab] OR “Seizures-intellectual Disability Due To Hydroxylysinuria Syndrome”[tiab] OR “Seizures-scoliosis-macrocephaly Syndrome”[tiab] OR “Semilobar Holoprosencephaly”[tiab] OR “Senior-loken Syndrome 7”[tiab] OR “Septo-optic Dysplasia Spectrum”[tiab] OR “Septopreoptic Holoprosencephaly”[tiab] OR “Severe Achondroplasia-developmental Delay-acanthosis Nigricans Syndrome”[tiab] OR “Severe Growth Deficiency-strabismus-extensive Dermal Melanocytosis-intellectual Disability Syndrome”[tiab] OR “Severe Intellectual Disability And Progressive Spastic Paraplegia”[tiab] OR “Severe Intellectual Disability-corpus Callosum Agenesis-facial Dysmorphism-cerebellar Ataxia Syndrome”[tiab] OR “Severe Intellectual Disability-epilepsy-anal Anomalies-distal Phalangeal Hypoplasia”[tiab] OR “Severe Intellectual Disability-hypotonia-strabismus-coarse Face-planovalgus Syndrome”[tiab] OR “Severe Intellectual Disability-poor Language-strabismus-grimacing Face-long Fingers Syndrome”[tiab] OR “Severe Intellectual Disability-progressive Postnatal Microcephaly-midline Stereotypic Hand Movements Syndrome”[tiab] OR “Severe Intellectual Disability-progressive Spastic Diplegia Syndrome”[tiab] OR “Severe Intellectual Disability-short Stature-behavioral Abnormalities-facial Dysmorphism Syndrome”[tiab] OR “Severe Neonatal Hypotonia-seizures-encephalopathy Syndrome Due To 5q31.3 Microdeletion”[tiab] OR “Severe Neurodevelopmental Disorder With Feeding Difficulties-stereotypic Hand Movement-bilateral Cataract”[tiab] OR “Severe Oculo-renal-cerebellar Syndrome”[tiab] OR “Severe Phosphoribosylpyrophosphate Synthetase Superactivity”[tiab] OR “Severe X-linked Intellectual Disability, Gustavson Type”[tiab] OR “Shaheen Syndrome”[tiab] OR “Shashi-pena Syndrome”[tiab] OR “Short Stature, Brachydactyly, Intellectual Developmental Disability, And Seizures”[tiab] OR “Short Stature, Developmental Delay, And Congenital Heart Defects”[tiab] OR “Short Stature, Facial Dysmorphism, And Skeletal Anomalies With Or Without Cardiac Anomalies”[tiab] OR “Short Stature, Hearing Loss, Retinitis Pigmentosa, And Distinctive Facies”[tiab] OR “Short Stature, Rhizomelic, With Microcephaly, Micrognathia, And Developmental Delay”[tiab] OR “Short Stature-brachydactyly-obesity-global Developmental Delay Syndrome”[tiab] OR “Short Stature-craniofacial Anomalies-genital Hypoplasia Syndrome”[tiab] OR “Short Stature-delayed Bone Age Due To Thyroid Hormone Metabolism Deficiency”[tiab] OR “Short Stature-webbed Neck-heart Disease Syndrome”[tiab] OR “Short Ulna-dysmorphism-hypotonia-intellectual Disability Syndrome”[tiab] OR “Short-rib Thoracic Dysplasia 10 With Or Without Polydactyly”[tiab] OR “Shprintzen-goldberg Craniosynostosis Syndrome”[tiab] OR “Shprintzen-goldberg Syndrome”[tiab] OR “Shwachman-diamond Syndrome”[tiab] OR “Shwachman-diamond Syndrome 1”[tiab] OR “Sialidosis Type 1”[tiab] OR “Sialuria”[tiab] OR “Siderius X-linked Mental Retardation Syndrome”[tiab] OR “Sifrim-hitz-weiss Syndrome”[tiab] OR “Silver-russell Syndrome”[tiab] OR “Silver-russell Syndrome Due To 7p11.2p13 Microduplication”[tiab] OR “Silver-russell Syndrome Due To An Imprinting Defect Of 11p15”[tiab] OR “Sim1-related Prader-willi-like Syndrome”[tiab] OR “Simha Syndrome”[tiab] OR “Simpson-golabi-behmel Syndrome”[tiab] OR “Simpson-golabi-behmel Syndrome, Type 2”[tiab] OR “Sin3a-related Intellectual Disability Syndrome Due To A Point Mutation”[tiab] OR “Sjogren-larsson Syndrome”[tiab] OR “Sjögren-larsson Syndrome”[tiab] OR “Skeletal Defects, Genital Hypoplasia, And Mental Retardation”[tiab] OR “Skeletal Dysplasia-epilepsy-short Stature Syndrome”[tiab] OR “Skeletal Dysplasia-t-cell Immunodeficiency-developmental Delay Syndrome”[tiab] OR “Skin Creases, Congenital Symmetric Circumferential, 1”[tiab] OR “Skin Creases, Congenital Symmetric Circumferential, 2”[tiab] OR “Skraban-deardorff Syndrome”[tiab] OR “Slc35a2-cdg”[tiab] OR “Slc39a8-cdg”[tiab] OR “Smith-kingsmore Syndrome”[tiab] OR “Smith-lemli-opitz Syndrome”[tiab] OR “Smith-magenis Syndrome”[tiab] OR “Snijders Blok-fisher Syndrome”[tiab] OR “Solitary Median Maxillary Central Incisor”[tiab] OR “Sonoda Syndrome”[tiab] OR “Sotos Syndrome”[tiab] OR “Spastic Ataxia 9, Autosomal Recessive”[tiab] OR “Spastic Ataxia, Charlevoix-saguenay Type”[tiab] OR “Spastic Diplegia, Infantile Type”[tiab] OR “Spastic Paraplegia 11, Autosomal Recessive”[tiab] OR “Spastic Paraplegia 14, Autosomal Recessive”[tiab] OR “Spastic Paraplegia 15, Autosomal Recessive”[tiab] OR “Spastic Paraplegia 16, X-linked”[tiab] OR “Spastic Paraplegia 18, Autosomal Recessive”[tiab] OR “Spastic Paraplegia 2, X-linked”[tiab] OR “Spastic Paraplegia 20, Autosomal Recessive”[tiab] OR “Spastic Paraplegia 26, Autosomal Recessive”[tiab] OR “Spastic Paraplegia 3, Autosomal Dominant”[tiab] OR “Spastic Paraplegia 32, Autosomal Recessive”[tiab] OR “Spastic Paraplegia 35, Autosomal Recessive”[tiab] OR “Spastic Paraplegia 4, Autosomal Dominant”[tiab] OR “Spastic Paraplegia 45, Autosomal Recessive”[tiab] OR “Spastic Paraplegia 46, Autosomal Recessive”[tiab] OR “Spastic Paraplegia 47, Autosomal Recessive”[tiab] OR “Spastic Paraplegia 48, Autosomal Recessive”[tiab] OR “Spastic Paraplegia 50, Autosomal Recessive”[tiab] OR “Spastic Paraplegia 51, Autosomal Recessive”[tiab] OR “Spastic Paraplegia 52, Autosomal Recessive”[tiab] OR “Spastic Paraplegia 54, Autosomal Recessive”[tiab] OR “Spastic Paraplegia 55, Autosomal Recessive”[tiab] OR “Spastic Paraplegia 56, Autosomal Recessive”[tiab] OR “Spastic Paraplegia 61, Autosomal Recessive”[tiab] OR “Spastic Paraplegia 63, Autosomal Recessive”[tiab] OR “Spastic Paraplegia 64, Autosomal Recessive”[tiab] OR “Spastic Paraplegia 81, Autosomal Recessive”[tiab] OR “Spastic Paraplegia 82, Autosomal Recessive”[tiab] OR “Spastic Paraplegia 9b, Autosomal Recessive”[tiab] OR “Spastic Paraplegia And Psychomotor Retardation With Or Without Seizures”[tiab] OR “Spastic Paraplegia Type 2”[tiab] OR “Spastic Paraplegia, Ataxia, And Mental Retardation”[tiab] OR “Spastic Paraplegia, Epilepsy, And Mental Retardation”[tiab] OR “Spastic Paraplegia, Intellectual Disability, Nystagmus, And Obesity”[tiab] OR “Spastic Paraplegia, Sensorineural Deafness, Mental Retardation, And”[tiab] OR “Spastic Paraplegia-glaucoma-intellectual Disability Syndrome”[tiab] OR “Spastic Paraplegia-intellectual Disability-nystagmus-obesity Syndrome”[tiab] OR “Spastic Paraplegia-nephritis-deafness Syndrome”[tiab] OR “Spastic Paraplegia-precocious Puberty Syndrome”[tiab] OR “Spastic Paraplegia-severe Developmental Delay-epilepsy Syndrome”[tiab] OR “Spastic Paresis, Glaucoma, And Mental Retardation”[tiab] OR “Spastic Quadriplegia, Retinitis Pigmentosa, And Mental Retardation”[tiab] OR “Spastic Tetraplegia, Thin Corpus Callosum, And Progressive Microcephaly”[tiab] OR “Spastic Tetraplegia-retinitis Pigmentosa-intellectual Disability Syndrome”[tiab] OR “Spastic Tetraplegia-thin Corpus Callosum-progressive Postnatal Microcephaly Syndrome”[tiab] OR “Spectrin-associated Autosomal Recessive Cerebellar Ataxia”[tiab] OR “Spinal Muscular Atrophy With Mental Retardation”[tiab] OR “Spinal Muscular Atrophy With Microcephaly And Mental Subnormality”[tiab] OR “Spinal Muscular Atrophy-progressive Myoclonic Epilepsy Syndrome”[tiab] OR “Spinocerebellar Ataxia 13”[tiab] OR “Spinocerebellar Ataxia 21”[tiab] OR “Spinocerebellar Ataxia 27”[tiab] OR “Spinocerebellar Ataxia 29, Congenital Nonprogressive”[tiab] OR “Spinocerebellar Ataxia 42, Early-onset, Severe, With Neurodevelopmental Deficits”[tiab] OR “Spinocerebellar Ataxia 47”[tiab] OR “Spinocerebellar Ataxia Type 13”[tiab] OR “Spinocerebellar Ataxia Type 21”[tiab] OR “Spinocerebellar Ataxia Type 27”[tiab] OR “Spinocerebellar Ataxia Type 35”[tiab] OR “Spinocerebellar Ataxia, Autosomal Recessive 10”[tiab] OR “Spinocerebellar Ataxia, Autosomal Recessive 12”[tiab] OR “Spinocerebellar Ataxia, Autosomal Recessive 13”[tiab] OR “Spinocerebellar Ataxia, Autosomal Recessive 15”[tiab] OR “Spinocerebellar Ataxia, Autosomal Recessive 17”[tiab] OR “Spinocerebellar Ataxia, Autosomal Recessive 18”[tiab] OR “Spinocerebellar Ataxia, Autosomal Recessive 2”[tiab] OR “Spinocerebellar Ataxia, Autosomal Recessive 21”[tiab] OR “Spinocerebellar Ataxia, Autosomal Recessive 22”[tiab] OR “Spinocerebellar Ataxia, Autosomal Recessive 23”[tiab] OR “Spinocerebellar Ataxia, Autosomal Recessive 28”[tiab] OR “Spinocerebellar Ataxia, Autosomal Recessive 29”[tiab] OR “Spinocerebellar Ataxia, Autosomal Recessive 30”[tiab] OR “Spinocerebellar Ataxia, Autosomal Recessive 4”[tiab] OR “Spinocerebellar Degeneration And Corneal Dystrophy”[tiab] OR “Spinocerebellar Degeneration-corneal Dystrophy Syndrome”[tiab] OR “Split-hand/foot Malformation 1”[tiab] OR “Split-hand/foot Malformation 3”[tiab] OR “Sponastrime Dysplasia”[tiab] OR “Spondylo-ocular Syndrome”[tiab] OR “Spondyloenchondrodysplasia”[tiab] OR “Spondyloenchondrodysplasia With Immune Dysregulation”[tiab] OR “Spondyloepimetaphyseal Dysplasia With Joint Laxity”[tiab] OR “Spondyloepimetaphyseal Dysplasia, Faden-alkuraya Type”[tiab] OR “Spondyloepimetaphyseal Dysplasia, Genevieve Type”[tiab] OR “Spondyloepimetaphyseal Dysplasia, Sponastrime Type”[tiab] OR “Spondyloepimetaphyseal Dysplasia, X-linked, With Mental Deterioration”[tiab] OR “Spondyloepiphyseal Dysplasia Tarda With Mental Retardation”[tiab] OR “Spondyloepiphyseal Dysplasia Tarda, Kohn Type”[tiab] OR “Spondylometaphyseal Dysplasia, X-linked”[tiab] OR “Sporadic Fetal Brain Disruption Sequence”[tiab] OR “Srd5a3-cdg”[tiab] OR “Ssr4-cdg”[tiab] OR “Stag1-related Intellectual Disability-facial Dysmorphism-gastroesophageal Reflux Syndrome”[tiab] OR “Stankiewicz-isidor Syndrome”[tiab] OR “Stargardt Macular Degeneration, Absent Or Hypoplastic Corpus Callosum,mental Retardation, And Dysmorphic Features”[tiab] OR “Steinert Myotonic Dystrophy”[tiab] OR “Stevenson-carey Syndrome”[tiab] OR “Stickler Syndrome Type 1”[tiab] OR “Stimmler Syndrome”[tiab] OR “Stocco Dos Santos X-linked Mental Retardation Syndrome”[tiab] OR “Stomatin-deficient Cryohydrocytosis With Neurologic Defects”[tiab] OR “Striatal Degeneration, Autosomal Dominant 2”[tiab] OR “Striatonigral Degeneration, Infantile”[tiab] OR “Striatonigral Degeneration, Infantile, Mitochondrial”[tiab] OR “Structural Brain Anomalies With Impaired Intellectual Development And Craniosynostosis”[tiab] OR “Stt3a-cdg”[tiab] OR “Stt3b-cdg”[tiab] OR “Sturge-weber Syndrome”[tiab] OR “Subaortic Stenosis--short Stature Syndrome”[tiab] OR “Subaortic Stenosis-short Stature Syndrome”[tiab] OR “Succinic Semialdehyde Dehydrogenase Deficiency”[tiab] OR “Sucrosuria, Hiatus Hernia And Mental Retardation”[tiab] OR “Sulfite Oxidase Deficiency”[tiab] OR “Summitt Syndrome”[tiab] OR “Suprabulbar Paresis, Congenital”[tiab] OR “Symptomatic Form Of Fragile X Syndrome In Female Carriers”[tiab] OR “Synaptic Congenital Myasthenic Syndromes”[tiab] OR “Syndromic Diarrhea”[tiab] OR “Syndromic Recessive X-linked Ichthyosis”[tiab] OR “Syndromic X-linked Intellectual Disability 7”[tiab] OR “Syngap1-related Developmental And Epileptic Encephalopathy”[tiab] OR “T-substance Anomaly”[tiab] OR “Takenouchi-kosaki Syndrome”[tiab] OR “Tall Stature-intellectual Disability-renal Anomalies Syndrome”[tiab] OR “Tarp Syndrome”[tiab] OR “Tatton-brown-rahman Syndrome”[tiab] OR “Telecanthus”[tiab] OR “Telo2-related Intellectual Disability-neurodevelopmental Disorder”[tiab] OR “Temple Syndrome”[tiab] OR “Temple Syndrome Due To Maternal Uniparental Disomy Of Chromosome 14”[tiab] OR “Temple Syndrome Due To Paternal 14q32.2 Hypomethylation”[tiab] OR “Temple Syndrome Due To Paternal 14q32.2 Microdeletion”[tiab] OR “Temple-baraitser Syndrome”[tiab] OR “Temtamy Preaxial Brachydactyly Syndrome”[tiab] OR “Temtamy Syndrome”[tiab] OR “Tenorio Syndrome”[tiab] OR “Tetra-amelia With Ectodermal Dysplasia And Lacrimal Duct Abnormalities”[tiab] OR “Tetramelic Deficiencies, Ectodermal Dysplasia, Deformed Ears, Andother Abnormalities”[tiab] OR “Tetrasomy 12p”[tiab] OR “Tetrasomy 18p”[tiab] OR “Thanatophoric Dysplasia”[tiab] OR “Thanatophoric Dysplasia Type 1”[tiab] OR “Thanatophoric Dysplasia, Type I”[tiab] OR “Thauvin-robinet-faivre Syndrome”[tiab] OR “Thoc6-related Developmental Delay-microcephaly-facial Dysmorphism Syndrome”[tiab] OR “Thoracic Dysplasia-hydrocephalus Syndrome”[tiab] OR “Thrombocytopenia, Paris-trousseau Type”[tiab] OR “Thrombocytopenia-absent Radius Syndrome”[tiab] OR “Thumb Deformity And Alopecia”[tiab] OR “Thumb Deformity-alopecia-pigmentation Anomaly Syndrome”[tiab] OR “Thumb Stiffness-brachydactyly-intellectual Disability Syndrome”[tiab] OR “Thumbs, Stiff, With Brachydactyly Type A1 And Developmental Delay”[tiab] OR “Thyroid Ectopia”[tiab] OR “Thyroid Hormonogenesis, Genetic Defect In, 1”[tiab] OR “Thyroid Hormonogenesis, Genetic Defect In, 3”[tiab] OR “Thyroid Hormonogenesis, Genetic Defect In, 4”[tiab] OR “Thyroid Hormonogenesis, Genetic Defect In, 5”[tiab] OR “Thyroid Hypoplasia”[tiab] OR “Thyrotropin-releasing Hormone Deficiency”[tiab] OR “Timothy Syndrome”[tiab] OR “Tmem70-related Mitochondrial Encephalo-cardio-myopathy”[tiab] OR “Tonne-kalscheuer Syndrome”[tiab] OR “Townes-brocks Syndrome”[tiab] OR “Townes-brocks Syndrome 1”[tiab] OR “Transcobalamin Ii Deficiency”[tiab] OR “Transketolase Deficiency”[tiab] OR “Trappc11-related Limb-girdle Muscular Dystrophy R18”[tiab] OR “Treacher Collins Syndrome 1”[tiab] OR “Tremor-ataxia-central Hypomyelination Syndrome”[tiab] OR “Trichorhinophalangeal Syndrome Type 2”[tiab] OR “Trichorhinophalangeal Syndrome, Type Ii”[tiab] OR “Trichothiodystrophy 1, Photosensitive”[tiab] OR “Trichothiodystrophy 2, Photosensitive”[tiab] OR “Trichothiodystrophy 3, Photosensitive”[tiab] OR “Trichothiodystrophy 4, Nonphotosensitive”[tiab] OR “Trichothiodystrophy 5, Nonphotosensitive”[tiab] OR “Trichothiodystrophy 6, Nonphotosensitive”[tiab] OR “Trichothiodystrophy 9, Nonphotosensitive”[tiab] OR “Triglyceride Deposit Cardiomyovasculopathy”[tiab] OR “Trigonocephaly With Short Stature And Developmental Delay”[tiab] OR “Trigonocephaly-short Stature-developmental Delay Syndrome”[tiab] OR “Trisomy 10p”[tiab] OR “Trisomy 12p”[tiab] OR “Trisomy 13”[tiab] OR “Trisomy 17p”[tiab] OR “Trisomy 18p”[tiab] OR “Trisomy 5p”[tiab] OR “Trisomy 8p”[tiab] OR “Trisomy 9p”[tiab] OR “Tryptophanuria With Dwarfism”[tiab] OR “Tuberous Sclerosis Complex”[tiab] OR “Tuberous Sclerosis-1”[tiab] OR “Tuberous Sclerosis-2”[tiab] OR “Turnpenny-fry Syndrome”[tiab] OR “Tyrosine Transaminase Deficiency”[tiab] OR “Tyrosinemia Type 2”[tiab] OR “Tyrosinemia, Type Iii”[tiab] OR “Tyshchenko Syndrome”[tiab] OR “Ulna Hypoplasia-intellectual Disability Syndrome”[tiab] OR “Ulnar Hypoplasia With Mental Retardation”[tiab] OR “Unilateral Focal Polymicrogyria”[tiab] OR “Unilateral Hemispheric Polymicrogyria”[tiab] OR “Unilateral Polymicrogyria”[tiab] OR “Urban-rogers-meyer Syndrome”[tiab] OR “Urocanase Deficiency”[tiab] OR “Usher Syndrome Type 1”[tiab] OR “Usmani-riazuddin Syndrome, Autosomal Dominant”[tiab] OR “Usmani-riazuddin Syndrome, Autosomal Recessive”[tiab] OR “Uveal Coloboma-cleft Lip And Palate-intellectual Disability”[tiab] OR “Vacterl With Hydrocephalus”[tiab] OR “Van Bogaert-hozay Syndrome”[tiab] OR “Van Den Bosch Syndrome”[tiab] OR “Van Esch-o'driscoll Syndrome”[tiab] OR “Van Maldergem Syndrome 1”[tiab] OR “Van Maldergem Syndrome 2”[tiab] OR “Velocardiofacial Syndrome”[tiab] OR “Ventricular Extrasystoles With Syncopal Episodes-perodactyly-robin Sequence Syndrome”[tiab] OR “Vertebral, Cardiac, Renal, And Limb Defects Syndrome 1”[tiab] OR “Ververi-brady Syndrome”[tiab] OR “Vici Syndrome”[tiab] OR “Viss Syndrome”[tiab] OR “Vissers-bodmer Syndrome”[tiab] OR “Vitamin B12-responsive Methylmalonic Acidemia”[tiab] OR “Vitamin B12-unresponsive Methylmalonic Acidemia”[tiab] OR “Vitamin B12-unresponsive Methylmalonic Acidemia Type Mut-”[tiab] OR “Vitamin B12-unresponsive Methylmalonic Acidemia Type Mut0”[tiab] OR “Vitamin K Antagonist Embryofetopathy”[tiab] OR “Vitiligo, Progressive, With Mental Retardation And Urethral Duplication”[tiab] OR “Vps11-related Autosomal Recessive Hypomyelinating Leukodystrophy”[tiab] OR “Waardenburg Syndrome Type 3”[tiab] OR “Waardenburg Syndrome, Type 2e”[tiab] OR “Waardenburg Syndrome, Type 3”[tiab] OR “Wac-related Facial Dysmorphism-developmental Delay-behavioral Abnormalities Syndrome”[tiab] OR “Wagr 11p13 Deletion Syndrome”[tiab] OR “Wagr Syndrome”[tiab] OR “Waisman Syndrome”[tiab] OR “Walker-warburg Syndrome”[tiab] OR “Warburg Micro Syndrome 1”[tiab] OR “Warburg Micro Syndrome 2”[tiab] OR “Warburg Micro Syndrome 3”[tiab] OR “Warburg Micro Syndrome 4”[tiab] OR “Wars2-related Combined Oxidative Phosphorylation Defect”[tiab] OR “Warsaw Breakage Syndrome”[tiab] OR “Weaver Syndrome”[tiab] OR “Weaver-williams Syndrome”[tiab] OR “Weill-marchesani Syndrome”[tiab] OR “Weill-marchesani Syndrome 1”[tiab] OR “Weill-marchesani Syndrome 2, Dominant”[tiab] OR “Weismann-netter Syndrome”[tiab] OR “Weiss-kruszka Syndrome”[tiab] OR “White Matter Hypoplasia-corpus Callosum Agenesis-intellectual Disability Syndrome”[tiab] OR “White-kernohan Syndrome”[tiab] OR “White-sutton Syndrome”[tiab] OR “Wieacker-wolff Syndrome”[tiab] OR “Wiedemann-rautenstrauch Syndrome”[tiab] OR “Wiedemann-steiner Syndrome”[tiab] OR “Williams Syndrome”[tiab] OR “Williams-beuren Syndrome”[tiab] OR “Williams-beuren Syndrome (wbs)”[tiab] OR “Wilms Tumor, Aniridia, Genitourinary Anomalies, And Mental Retardationsyndrome”[tiab] OR “Wilson Disease”[tiab] OR “Wilson-turner Syndrome”[tiab] OR “Witteveen-kolk Syndrome”[tiab] OR “Wolcott-rallison Syndrome”[tiab] OR “Wolf-hirschhorn Syndrome”[tiab] OR “Wolfram Syndrome”[tiab] OR “Wolfram Syndrome 1”[tiab] OR “Wolfram Syndrome, Mitochondrial Form”[tiab] OR “Woodhouse-sakati Syndrome”[tiab] OR “Woods Syndrome”[tiab] OR “Wrinkly Skin Syndrome”[tiab] OR “Wyburn-mason Syndrome”[tiab] OR “X Small Rings”[tiab] OR “X-linked Acrogigantism”[tiab] OR “X-linked Adrenoleukodystrophy”[tiab] OR “X-linked Cerebral-cerebellar-coloboma Syndrome”[tiab] OR “X-linked Charcot-marie-tooth Disease Type 2”[tiab] OR “X-linked Charcot-marie-tooth Disease Type 4”[tiab] OR “X-linked Complicated Corpus Callosum Dysgenesis”[tiab] OR “X-linked Complicated Spastic Paraplegia Type 1”[tiab] OR “X-linked Creatine Transporter Deficiency”[tiab] OR “X-linked Dominant Chondrodysplasia, Chassaing-lacombe Type”[tiab] OR “X-linked Female Restricted Facial Dysmorphism-short Stature-choanal Atresia-intellectual Disability”[tiab] OR “X-linked Intellectual Disability Due To Gria3 Mutations”[tiab] OR “X-linked Intellectual Disability With Isolated Growth Hormone Deficiency”[tiab] OR “X-linked Intellectual Disability, Abidi Type”[tiab] OR “X-linked Intellectual Disability, Armfield Type”[tiab] OR “X-linked Intellectual Disability, Cabezas Type”[tiab] OR “X-linked Intellectual Disability, Cantagrel Type”[tiab] OR “X-linked Intellectual Disability, Cilliers Type”[tiab] OR “X-linked Intellectual Disability, Golabi-ito-hall Type”[tiab] OR “X-linked Intellectual Disability, Hedera Type”[tiab] OR “X-linked Intellectual Disability, Miles-carpenter Type”[tiab] OR “X-linked Intellectual Disability, Najm Type”[tiab] OR “X-linked Intellectual Disability, Nascimento Type”[tiab] OR “X-linked Intellectual Disability, Pai Type”[tiab] OR “X-linked Intellectual Disability, Porteous Type”[tiab] OR “X-linked Intellectual Disability, Schimke Type”[tiab] OR “X-linked Intellectual Disability, Seemanova Type”[tiab] OR “X-linked Intellectual Disability, Shashi Type”[tiab] OR “X-linked Intellectual Disability, Shrimpton Type”[tiab] OR “X-linked Intellectual Disability, Siderius Type”[tiab] OR “X-linked Intellectual Disability, Snyder Type”[tiab] OR “X-linked Intellectual Disability, Stevenson Type”[tiab] OR “X-linked Intellectual Disability, Stocco Dos Santos Type”[tiab] OR “X-linked Intellectual Disability, Stoll Type”[tiab] OR “X-linked Intellectual Disability, Sutherland-haan Type”[tiab] OR “X-linked Intellectual Disability, Van Esch Type”[tiab] OR “X-linked Intellectual Disability, Wilson Type”[tiab] OR “X-linked Intellectual Disability-acromegaly-hyperactivity Syndrome”[tiab] OR “X-linked Intellectual Disability-ataxia-apraxia Syndrome”[tiab] OR “X-linked Intellectual Disability-cardiomegaly-congestive Heart Failure Syndrome”[tiab] OR “X-linked Intellectual Disability-cerebellar Hypoplasia Syndrome”[tiab] OR “X-linked Intellectual Disability-cerebellar Hypoplasia-spondylo-epiphyseal Dysplasia Syndrome”[tiab] OR “X-linked Intellectual Disability-craniofacioskeletal Syndrome”[tiab] OR “X-linked Intellectual Disability-cubitus Valgus-dysmorphism Syndrome”[tiab] OR “X-linked Intellectual Disability-dysmorphism-cerebral Atrophy Syndrome”[tiab] OR “X-linked Intellectual Disability-epilepsy-progressive Joint Contractures-dysmorphism Syndrome”[tiab] OR “X-linked Intellectual Disability-global Development Delay-facial Dysmorphism-sacral Caudal Remnant Syndrome”[tiab] OR “X-linked Intellectual Disability-hypogammaglobulinemia-progressive Neurological Deterioration Syndrome”[tiab] OR “X-linked Intellectual Disability-hypogonadism-ichthyosis-obesity-short Stature Syndrome”[tiab] OR “X-linked Intellectual Disability-hypotonia-facial Dysmorphism-aggressive Behavior Syndrome”[tiab] OR “X-linked Intellectual Disability-hypotonia-movement Disorder Syndrome”[tiab] OR “X-linked Intellectual Disability-limb Spasticity-retinal Dystrophy-diabetes Insipidus Syndrome”[tiab] OR “X-linked Intellectual Disability-macrocephaly-macroorchidism Syndrome”[tiab] OR “X-linked Intellectual Disability-plagiocephaly Syndrome”[tiab] OR “X-linked Intellectual Disability-psychosis-macroorchidism Syndrome”[tiab] OR “X-linked Intellectual Disability-retinitis Pigmentosa Syndrome”[tiab] OR “X-linked Intellectual Disability-seizures-psoriasis Syndrome”[tiab] OR “X-linked Intellectual Disability-short Stature-overweight Syndrome”[tiab] OR “X-linked Lissencephaly With Abnormal Genitalia”[tiab] OR “X-linked Neurodegenerative Syndrome, Bertini Type”[tiab] OR “X-linked Neurodegenerative Syndrome, Hamel Type”[tiab] OR “X-linked Non-syndromic Intellectual Disability”[tiab] OR “X-linked Spastic Paraplegia Type 16”[tiab] OR “X-linked Spasticity-intellectual Disability-epilepsy Syndrome”[tiab] OR “Xeroderma Pigmentosum”[tiab] OR “Xeroderma Pigmentosum, Complementation Group A”[tiab] OR “Xeroderma Pigmentosum, Complementation Group B”[tiab] OR “Xeroderma Pigmentosum, Complementation Group D”[tiab] OR “Xeroderma Pigmentosum, Complementation Group F”[tiab] OR “Xeroderma Pigmentosum-cockayne Syndrome Complex”[tiab] OR “Xfe Progeroid Syndrome”[tiab] OR “Xia-gibbs Syndrome”[tiab] OR “Xp11.22 Microduplication Syndrome”[tiab] OR “Xp21 Deletion Syndrome”[tiab] OR “Xp22.13p22.2 Duplication Syndrome”[tiab] OR “Xq12-q13.3 Duplication Syndrome”[tiab] OR “Xq21 Deletion Syndrome”[tiab] OR “Xq21 Microdeletion Syndrome”[tiab] OR “Xq25 Duplication Syndrome”[tiab] OR “Xq25 Microduplication Syndrome”[tiab] OR “Xq27.3q28 Duplication Syndrome”[tiab] OR “Xq28 (mecp2) Duplication”[tiab] OR “Xy Type Gonadal Dysgenesis-associated Anomalies Syndrome”[tiab] OR “Xylt1-cdg”[tiab] OR “Yuan-harel-lupski Syndrome”[tiab] OR “Zaki Syndrome”[tiab] OR “Zechi-ceide Syndrome”[tiab] OR “Zellweger-like Syndrome Without Peroxisomal Anomalies”[tiab] OR “Zimmermann-laband Syndrome”[tiab] OR “Zimmermann-laband Syndrome 1”[tiab] OR “Zimmermann-laband Syndrome 2”[tiab] OR “Zimmermann-laband Syndrome 3”[tiab] OR “Zttk Syndrome”[tiab] OR “Zunich Neuroectodermal Syndrome”[tiab] |

| **Search** | **String (EMBASE)** |
| --- | --- |
| **ID** | Developmental Disabilities'/exp OR 'Intellectual Disability'/exp OR 'Learning Disabilities'/exp OR 'Persons with Mental Disabilities'/exp OR 'Development Deviation*' OR 'Development Disorder*' OR 'Developmental Academic Disabilit*' OR 'Developmental Academic Disorder*' OR 'Developmental Delay Disorder*' OR 'Developmental Disabilit*' OR 'Developmental Disorders of Scholastic Skills' OR 'Idiocy' OR 'Intellectual Development Disorder*' OR 'Intellectual Disabilit*' OR 'Learning Disabilit*' OR 'Learning Disturbance*' OR 'Mental Deficienc*' OR 'Mental Retardation' |
| **Cancer** | *Neoplasm/ or early cancer diagnosis/ or cancer registry/ or (Neoplas* or Tumor* or Tumour* or Cancer* or Malignan*).ti,kf. |
| **Incidence** | Epidemiology/ or case report/ or incidence/ or prevalence/ or (Epidemiologic Stud* or case Report* or case stud*OR Incidence* or prevalence*).ti,ab,kf. |
| **Chr, A** | ("10q22.3q23.3 microdeletion syndrome" or "10q22.3q23.3 microduplication syndrome" or "11p15.4 microduplication syndrome" or "11q22.2q22.3 microdeletion syndrome" or "12q14 microdeletion syndrome" or "13q12.3 microdeletion syndrome" or "14q11.2 microdeletion syndrome" or "14q24.1q24.3 microdeletion syndrome" or "15q11.2 microdeletion syndrome" or "15q11q13 microduplication syndrome" or "15q13.3 microdeletion syndrome" or "15q14 microdeletion syndrome" or "15q24 microdeletion syndrome" or "15q24 recurrent microdeletion syndrome" or "15q26 overgrowth syndrome" or "16p11.2p12.2 microdeletion syndrome" or "16p11.2-p12.2 microdeletion syndrome" or "16p11.2p12.2 microduplication syndrome" or "16p13.11 microdeletion syndrome" or "16p13.11 microduplication syndrome" or "16p13.2 microdeletion syndrome" or "16q24.3 microdeletion syndrome" or "17p11.2 microduplication syndrome" or "17q11 microdeletion syndrome" or "17q11.2 microduplication syndrome" or "17q12 microdeletion syndrome" or "17q12 microduplication syndrome" or "17q21.31 microdeletion syndrome" or "17q21.31 microduplication syndrome" or "17q21.31 recurrent microdeletion syndrome" or "17q24.2 microdeletion syndrome" or "19p13.13 microdeletion syndrome" or "19p13.3 microduplication syndrome" or "19q13.11 microdeletion syndrome" or "1p21.3 microdeletion syndrome" or "1p36 deletion syndrome" or "1p36 microdeletion syndrome" or "1q21.1 microdeletion syndrome" or "1q21.1 microduplication syndrome" or "1q41q42 microdeletion syndrome" or "1q44 microdeletion syndrome" or "20p13 microdeletion syndrome" or "20q11.2 microduplication syndrome" or "20q13.33 microdeletion syndrome" or "21q22.11q22.12 microdeletion syndrome" or "22q11 duplication syndrome" or "22q11.2 deletion syndrome" or "22q11.2 distal deletion syndrome" or "22q11.2 duplication syndrome" or "22q13 deletion syndrome" or "2p15-16.1 microdeletion syndrome" or "2p15p16.1 microdeletion syndrome" or "2p21 microdeletion syndrome" or "2q23.1 microdeletion syndrome" or "2q24 microdeletion syndrome" or "2q31.1 microdeletion syndrome" or "2q32q33 microdeletion syndrome" or "2q33.1 deletion syndrome" or "2q37 microdeletion syndrome" or "2q37 monosomy" or "3-methylglutaconic aciduria, type v" or "3c syndrome" or "3-hydroxy-3-methylglutaric aciduria" or "3mc syndrome" or "3-methylcrotonyl-coa carboxylase 1 deficiency" or "3-methylcrotonyl-coa carboxylase 2 deficiency" or "3-methylglutaconic aciduria type 3" or "3-methylglutaconic aciduria type 4" or "3-methylglutaconic aciduria type 7" or "3-methylglutaconic aciduria type 9" or "3-methylglutaconic aciduria with cataracts, neurologic involvement, and neutropenia" or "3-methylglutaconic aciduria with deafness, encephalopathy, and leigh-like syndrome" or "3-methylglutaconic aciduria, type ix" or "3-phosphoserine phosphatase deficiency" or "3q27.3 microdeletion syndrome" or "3q29 microdeletion syndrome" or "3q29 microduplication syndrome" or "45,x/46,xy mixed gonadal dysgenesis" or "47,xyy syndrome" or "48,xxxy syndrome" or "48,xxyy syndrome" or "48,xyyy syndrome" or "49,xxxxy syndrome" or "49,xxxyy syndrome" or "49,xyyyy syndrome" or "4q21 microdeletion syndrome" or "5q14.3 microdeletion syndrome" or "5q35 microduplication syndrome" or "6p22 microdeletion syndrome" or "6-pyruvoyl-tetrahydropterin synthase deficiency" or "6q terminal deletion syndrome" or "6q25 microdeletion syndrome" or "7q11.23 duplication syndrome" or "7q11.23 microduplication syndrome").ti,kf,ab. ("8p inverted duplication/deletion syndrome" or "8p11.2 deletion syndrome" or "8p23.1 deletion syndrome" or "8p23.1 duplication syndrome" or "8p23.1 microdeletion syndrome" or "8q21.11 microdeletion syndrome" or "8q24.3 microdeletion syndrome" or "9q subtelomeric deletion syndrome" or "9q31.1q31.3 microdeletion syndrome" or "9q33.3q34.11 microdeletion syndrome" or "aarskog-scott syndrome" or "abetal34v amyloidosis" or "absent eyebrows and eyelashes with mental retardation" or "achalasia-addisonianism-alacrima syndrome" or "achalasia-microcephaly syndrome" or "acquired partial lipodystrophy" or "acrocallosal syndrome" or "acrocardiofacial syndrome" or "acrodysostosis" or "acrofacial dysostosis, catania type" or "acrofrontofacionasal dysostosis" or "acrofrontofacionasal dysostosis 1" or "acrogeria" or "acromegaloid facial appearance syndrome" or "acromelic frontonasal dysostosis" or "acromelic frontonasal dysplasia" or "acropectoral syndrome" or "acropectorovertebral dysplasia" or "acute infantile liver failure-cerebellar ataxia-peripheral sensory motor neuropathy syndrome" or "adams-oliver syndrome" or "adams-oliver syndrome 1" or "adenylosuccinase deficiency" or "adenylosuccinate lyase deficiency" or "adult polyglucosan body disease" or "adult-onset autosomal recessive cerebellar ataxia" or "agenesis of cerebral white matter" or "agenesis of corpus callosum, cardiac, ocular, and genital syndrome" or "agenesis of the corpus callosum with peripheral neuropathy" or "ahdc1-related intellectual disability-obstructive sleep apnea-mild dysmorphism syndrome" or "aicar transformylase/imp cyclohydrolase deficiency" or "aicardi syndrome" or "aicardi-goutieres syndrome" or "aica-ribosiduria" or "al kaissi syndrome" or "alacrima, achalasia, and mental retardation syndrome" or "alacrimia-choreoathetosis-liver dysfunction syndrome" or "alagille syndrome" or "alazami syndrome" or "alazami-yuan syndrome" or "aldh18a1-related de barsy syndrome" or "alexander disease" or "alg11-cdg" or "alg12-cdg" or "alg1-cdg" or "alg2-cdg" or "alg9-cdg" or "alkuraya-kucinskas syndrome" or "allan-herndon-dudley syndrome" or "alobar holoprosencephaly" or "alopecia, neurologic defects, and endocrinopathy syndrome" or "alopecia, psychomotor epilepsy, pyorrhea, and mental subnormality" or "alopecia-contractures-dwarfism mental retardation syndrome" or "alopecia-contractures-dwarfism-intellectual disability syndrome" or "alopecia-epilepsy-oligophrenia syndrome of moynahan" or "alopecia-epilepsy-pyorrhea-intellectual disability syndrome" or "alopecia-intellectual disability syndrome" or "alopecia-intellectual disability-hypergonadotropic hypogonadism syndrome" or "alopecia-mental retardation syndrome " or "alpha-dystroglycan-related limb-girdle muscular dystrophy r16" or "alpha-mannosidosis" or "alpha-methylacetoacetic aciduria" or "alpha-n-acetylgalactosaminidase deficiency" or "alpha-thalassemia/mental retardation syndrome" or "alport syndrome - intellectual disability - midface hypoplasia - elliptocytosis" or "alport syndrome-intellectual disability-midface hypoplasia-elliptocytosis syndrome" or "alternating hemiplegia of childhood" or "amed syndrome" or "amelocerebrohypohidrotic syndrome" or "amino aciduria with mental deficiency, dwarfism, muscular dystrophy,osteoporosis, and acidosis" or "amyloidosis of gingiva and conjunctiva" or "amyotrophic dystonic paraplegia" or "anauxetic dysplasia" or "ane syndrome" or "angelman syndrome" or "aniridia, partial, with unilateral renal agenesis and psychomotorretardation" or "aniridia-cerebellar ataxia-intellectual disability syndrome" or "aniridia-intellectual disability syndrome" or "aniridia-ptosis-intellectual disability-familial obesity syndrome" or "ank3-related intellectual disability-sleep disturbance syndrome" or "anophthalmia/microphthalmia-esophageal atresia syndrome" or "antley-bixler syndrome" or "aortic arch anomaly with peculiar facies and mental retardation" or "aortic arch anomaly-facial dysmorphism-intellectual disability syndrome" or "apert syndrome" or "aphalangia, partial, with syndactyly and duplication of metatarsaliv" or "aphonia-deafness-retinal dystrophy-bifid halluces-intellectual disability syndrome" or "arachnodactyly-abnormal ossification-intellectual disability syndrome" or "arachnoid cyst" or "arboleda-tham syndrome" or "argininemia" or "argininosuccinic aciduria" or "arima syndrome" or "arterial tortuosity syndrome" or "arthrogryposis" or "arts syndrome" or "aspartylglucosaminuria" or "ataxia, deafness, and cardiomyopathy" or "ataxia, posterior column, with retinitis pigmentosa" or "ataxia, spastic, childhood-onset, autosomal recessive, with optic atrophy and mental retardation" or "ataxia-deafness-intellectual disability syndrome" or "ataxia-deafness-retardation syndrome" or "ataxia-intellectual disability-oculomotor apraxia-cerebellar cysts syndrome" or "ataxia-microcephaly-cataract syndrome" or "athyreosis" or "atkin-flaitz syndrome" or "atr-16 syndrome" or "attenuated ch diak-higashi syndrome" or "atypical juvenile parkinsonism" or "atypical rett syndrome" or "au-kline syndrome" or "aural atresia, multiple congenital anomalies, and mental retardation" or "aurocephalosyndactyly" or "autism spectrum disorder due to auts2 deficiency" or "autism spectrum disorder-epilepsy-arthrogryposis syndrome" or "autosomal dominant cerebellar ataxia" or "autosomal dominant charcot-marie-tooth disease type 2z" or "autosomal dominant deafness-onychodystrophy syndrome" or "autosomal dominant intellectual disability-craniofacial anomalies-cardiac defects syndrome" or "autosomal dominant nocturnal frontal lobe epilepsy" or "autosomal dominant non-syndromic intellectual disability" or "autosomal dominant robinow syndrome" or "autosomal dominant spastic paraplegia type 10" or "autosomal dominant spastic paraplegia type 4" or "autosomal recessive ataxia due to pex10 deficiency" or "autosomal recessive ataxia due to ubiquinone deficiency" or "autosomal recessive ataxia, beauce type" or "autosomal recessive axonal neuropathy with neuromyotonia" or "autosomal recessive centronuclear myopathy" or "autosomal recessive cerebellar ataxia due to cwf19l1 deficiency" or "autosomal recessive cerebellar ataxia with late-onset spasticity").ti,kf,ab. |
| **B, C, D** | ("autosomal recessive cerebellar ataxia-epilepsy-intellectual disability syndrome due to rubcn deficiency" or "autosomal recessive cerebellar ataxia-epilepsy-intellectual disability syndrome due to tud deficiency" or "autosomal recessive cerebellar ataxia-psychomotor delay syndrome" or "autosomal recessive cerebellar ataxia-pyramidal signs-nystagmus-oculomotor apraxia syndrome" or "autosomal recessive cerebelloparenchymal disorder type 3" or "autosomal recessive chorioretinopathy-microcephaly syndrome" or "autosomal recessive complex spastic paraplegia due to kennedy pathway dysfunction" or "autosomal recessive congenital cerebellar ataxia due to mglur1 deficiency" or "autosomal recessive cutis laxa type 1" or "autosomal recessive cutis laxa type 2, classic type" or "autosomal recessive cutis laxa type 2a" or "autosomal recessive distal osteolysis syndrome" or "autosomal recessive dopa-responsive dystonia" or "autosomal recessive hyperinsulinism due to kir6.2 deficiency" or "autosomal recessive non-syndromic intellectual disability" or "autosomal recessive omodysplasia" or "autosomal recessive primary microcephaly" or "autosomal recessive robinow syndrome" or "autosomal recessive spastic ataxia with leukoencephalopathy" or "autosomal recessive spastic paraplegia type" or "autosomal recessive spondylocostal dysostosis" or "autosomal spastic paraplegia type 58" or "axenfeld-rieger anomaly with partially absent eye muscles, distinctive face, hydrocephaly, and skeletal abnormalities" or "ayme-gripp syndrome" or "aym -gripp syndrome" or "bainbridge-ropers syndrome" or "baller-gerold syndrome" or "bamforth-lazarus syndrome" or "band heterotopia" or "band-like calcification with simplified gyration and polymicrogyria" or "bangstad syndrome" or "bannayan-riley-ruvalcaba syndrome" or "baraitser-winter cerebrofrontofacial syndrome" or "baraitser-winter syndrome" or "baralle-macken syndrome" or "barber-say syndrome" or "bardet-biedl syndrome" or "bartsocas-papas syndrome" or "bartter syndrome" or "basal cell nevus syndrome" or "basal ganglia calcification, idiopathic, childhood-onset" or "basel-vanagaite-smirin-yosef syndrome" or "basilicata-akhtar syndrome" or "beaulieu-boycott-innes syndrome" or "beck-fahrner syndrome" or "behr syndrome" or "benign adult familial myoclonic epilepsy" or "beta-ketothiolase deficiency" or "beta-mannosidosis" or "beta-mercaptolactate cysteine disulfiduria" or "beta-propeller protein-associated neurodegeneration" or "beta-ureidopropionase deficiency" or "biemond syndrome ii" or "biemond syndrome type 2" or "bifid nose" or "bilateral frontal polymicrogyria" or "bilateral frontoparietal polymicrogyria" or "bilateral generalized polymicrogyria" or "bilateral parasagittal parieto-occipital polymicrogyria" or "bilateral perisylvian polymicrogyria" or "bilateral polymicrogyria" or "congenital bile acid synthesis defect" or "biotinidase deficiency" or "birk-barel mental retardation dysmorphism syndrome" or "bjornstad syndrome" or "bj rnstad syndrome" or "blepharonasofacial malformation syndrome" or "blepharophimosis-intellectual disability syndrome" or "blepharophimosis with facial and genital anomalies and mental retardation" or "blepharophimosis with ptosis, syndactyly, and short stature" or "blepharophimosis-impaired intellectual development syndrome" or "blepharophimosis-ptosis-esotropia-syndactyly-short stature syndrome" or "bloom syndrome" or "body mass index quantitative trait locus 19" or "bohring-opitz syndrome" or "bonnemann-meinecke-reich syndrome" or "borjeson-forssman-lehmann syndrome" or "bosch-boonstra-schaaf optic atrophy syndrome" or "bosma arhinia microphthalmia syndrome" or "boucher-neuhauser syndrome" or "brachycephaly, deafness, cataract, microstomia, and mental retardation" or "brachydactyly, type a1" or "brachydactyly-nystagmus-cerebellar ataxia" or "brachydactyly-short stature-retinitis pigmentosa syndrome" or "brachymorphism-onychodysplasia-dysphalangism syndrome" or "brain malformations-musculoskeletal abnormalities-facial dysmorphism-intellectual disability syndrome" or "brain small vessel disease 1" or "brain-lung-thyroid syndrome" or "branched-chain ketoacid dehydrogenase kinase deficiency" or "branchiooculofacial syndrome" or "branchioskeletogenital syndrome" or "bresek syndrome" or "brooks-wisniewski-brown syndrome" or "brunner syndrome" or "bullous dystrophy, hereditary macular type" or "c syndrome" or "cahmr syndrome" or "camos syndrome" or "camptodactyly syndrome, guadalajara type 1" or "camptodactyly syndrome, guadalajara type 3" or "camptodactyly syndrome, guadalajara, type i" or "camptodactyly syndrome, guadalajara, type iii" or "camptodactyly, tall stature, and hearing loss syndrome" or "camptodactyly-fibrous tissue hyperplasia-skeletal dysplasia syndrome" or "carbamoyl phosphate synthetase i deficiency" or "cardiac-urogenital syndrome" or "cardiac-valvular ehlers-danlos syndrome" or "cardiocranial syndrome, pfeiffer type" or "cardiofaciocutaneous syndrome" or "carey-fineman-ziter syndrome" or "carnosinase deficiency" or "carnosinemia" or "carpenter syndrome" or "cat eye syndrome" or "cataract 11" or "cataract, ataxia, short stature, and mental retardation" or "cataract, microcephaly, failure to thrive, kyphoscoliosis syndrome" or "cataract-ataxia-deafness syndrome" or "cataract-ataxia-deafness-retardation syndrome" or "cataract-deafness-hypogonadism syndrome" or "cataract-hypertrichosis-intellectual disability syndrome" or "cataract-intellectual disability-anal atresia-urinary defects syndrome" or "cataract-intellectual disability-hypogonadism syndrome" or "cataract-nephropathy-encephalopathy syndrome" or "cat-eye syndrome" or "catifa syndrome" or "caudal appendage-deafness syndrome" or "cebalid syndrome" or "cednik syndrome" or "cephalin lipidosis" or "cerebellar ataxia, brain abnormalities, and cardiac conduction defects" or "cerebellar ataxia, mental retardation, and dysequilibrium syndrome " or "cerebellar ataxia, nonprogressive, with mental retardation" or "cerebellar atrophy, visual impairment, and psychomotor retardation" or "cerebellar-facial-dental syndrome" or "cerebellofaciodental syndrome" or "cerebral creatine deficiency syndrome 2" or "cerebral creatine deficiency syndrome 3" or "cerebral dysgenesis, neuropathy, ichthyosis, and palmoplantar keratoderma syndrome" or "cerebral pals spastic quadriplegic 2" or "cerebral visual impairment" or "cerebrocostomandibular syndrome" or "cerebrofacioarticular syndrome" or "cerebrofaciothoracic dysplasia" or "cerebrooculofacioskeletal syndrome 1" or "cerebrooculonasal syndrome" or "cerebrotendinous xanthomatosis" or "ceroid lipofuscinosis" or "chanarin-dorfman syndrome" or "char syndrome" or "charcot-marie-tooth disease" or "charge syndrome" or "chediak-higashi syndrome" or "ch diak-higashi syndrome" or "childhood absence epilepsy" or "childhood disintegrative disorder" or "childhood-onset motor and cognitive regression syndrome with extrapyramidal movement disorder" or "chime syndrome" or "chondrodysplasia punctata 2" or "chondrodysplasia with platyspondyly, distinctive brachydactyly, hydrocephaly, and microphthalmia" or "chondrodysplasia-disorder of sex development syndrome" or "chops syndrome" or "chorea, childhood-onset with psychomotor retardation" or "choroid plexus calcification and mental retardation" or "christianson syndrome" or "chromosome 10q26 deletion syndrome" or "chromosome 11p13 deletion syndrome" or "chromosome 13q14 deletion syndrome" or "chromosome 13q33-q34 deletion syndrome" or "chromosome 14q11-q22 deletion syndrome").ti,kf,ab. |
| **E, F, G, H** | ("chromosome 15q11.2 deletion syndrome" or "chromosome 15q11-q13 duplication syndrome" or "chromosome 15q13.3 microdeletion syndrome" or "chromosome 15q14 deletion syndrome" or "chromosome 15q26-qter deletion syndrome" or "chromosome 16p12.2-p11.2 deletion syndrome" or "chromosome 16p13.2 deletion syndrome" or "chromosome 16p13.3 deletion syndrome" or "chromosome 16p13.3 duplication syndrome" or "chromosome 17q11.2 deletion syndrome" or "chromosome 17q12 deletion syndrome" or "chromosome 17q12 duplication syndrome" or "chromosome 17q23.1-q23.2 deletion syndrome" or "chromosome 18p deletion syndrome" or "chromosome 18q deletion syndrome" or "chromosome 19p13.13 deletion syndrome" or "chromosome 19q13.11 deletion syndrome" or "chromosome 19q13.11 deletion syndrome" or "chromosome 1p35 deletion syndrome" or "chromosome 1p36 deletion syndrome" or "chromosome 1q21.1 deletion syndrome" or "chromosome 1q21.1 duplication syndrome" or "chromosome 1q41-q42 deletion syndrome" or "chromosome 20q11-q12 deletion syndrome" or "chromosome 22q11.2 duplication syndrome" or "chromosome 2p16.1-p15 deletion syndrome" or "chromosome 2q37 deletion syndrome" or "chromosome 3q29 deletion syndrome" or "chromosome 3q29 duplication syndrome" or "chromosome 5p13 duplication syndrome" or "chromosome 5q12 deletion syndrome" or "chromosome 6pter-p24 deletion syndrome" or "chromosome 6q25-q25 deletion syndrome" or "chromosome 8q21.11 deletion syndrome" or "chromosome 9p deletion syndrome" or "chromosome xp11.23-p11.22 duplication syndrome" or "chromosome xp11.3 deletion syndrome" or "chromosome xq27.3-q28 duplication syndrome" or "chronic bilirubin encephalopathy" or "chudley-mccullough syndrome" or "chylomicron retention disease" or "cimdag syndrome" or "cinca syndrome" or "citrullinemia type i" or "citrullinemia classic" or "ck syndrome" or "clark-baraitser syndrome" or "classic galactosemia" or "classic glucose transporter type 1 deficiency syndrome" or "classic homocystinuria" or "classic phenylketonuria" or "cleft lip/palate with abnormal thumbs and microcephaly" or "cleft lip/palate-ectodermal dysplasia syndrome" or "cleft palate, cardiac defect, genital anomalies, and ectrodactyly" or "cleft palate, cardiac defects, and mental retardation" or "cleft palate isolated" or "cleft palate-short stature-vertebral anomalies syndrome" or "cntnap2-related developmental and epileptic encephalopathy" or "coach syndrome 1" or "coach syndrome 2" or "cockayne syndrome" or "codas syndrome" or "coenzyme q10 deficiency" or "coffin-lowry syndrome" or "coffin-siris syndrome" or "cog1-cdg" or "cog2-cdg" or "cog5-cdg" or "cog8-cdg" or "cognitive impairment-coarse facies-heart defects-obesity-pulmonary involvement-short stature-skeletal dysplasia syndrome" or "cohen syndrome" or "cohen-gibson syndrome" or "coloboma-obesity-hypogenitalism-mental retardation syndrome" or "colobomatous microphthalmia-obesity-hypogenitalism-intellectual disability syndrome" or "combined immunodeficiency and megaloblastic anemia " or "combined immunodeficiency with faciooculoskeletal anomalies" or "combined oxidative phosphorylation defect type 23" or "combined oxidative phosphorylation defect type 27" or "combined oxidative phosphorylation deficiency 18" or "combined oxidative phosphorylation deficiency 24" or "combined oxidative phosphorylation deficiency 35" or "combined oxidative phosphorylation deficiency 36" or "cone-rod dystrophy 1" or "congenital cataracts, facial dysmorphism, and neuropathy" or "congenital cataracts-facial dysmorphism-neuropathy syndrome" or "congenital disorder of glycosylation" or "congenital generalized lipodystrophy" or "congenital heart defects, dysmorphic facial features, and intellectual developmental disorder" or "congenital hemidysplasia with ichthyosiform erythroderma and limb defects" or "congenital hydrocephalus" or "congenital hypothyroidism" or "congenital labioscrotal agenesis-cerebellar malformation-corneal dystrophy-facial dysmorphism syndrome" or "congenital muscular dystrophy with cerebellar involvement" or "congenital muscular dystrophy with intellectual disability" or "congenital muscular dystrophy, fukuyama type" or "congenital myasthenic syndrome" or "congenital progressive bone marrow failure-b-cell immunodeficiency-skeletal dysplasia syndrome" or "congenital rubella syndrome" or "congenital varicella syndrome" or "cono-spondylar dysplasia" or "cooper-jabs syndrome" or "corneal hypesthesia with retinal abnormalities, sensorineural deafness, unusual facies, persistent ductus arteriosus, and mental retardation" or "cornelia de lange syndrome" or "ageneis of corpus callosum" or "corpus callosum agenesis " or "corpus callosum, agenesis of" or "cortical blindness-intellectual disability-polydactyly syndrome" or "cortical dysgenesis with pontocerebellar hypoplasia due to tubb3 mutation" or "cortical dysplasia, complex, with other brain malformations " or "costello syndrome" or "cowchock syndrome" or "cowden syndrome" or "craniodiaphyseal dysplasia" or "craniodigital-intellectual disability syndrome" or "craniofacial dysmorphism, skeletal anomalies, and mental retardation syndrome" or "craniofacial dyssynostosis with short stature" or "craniofaciofrontodigital syndrome" or "craniofrontonasal dysplasia" or "craniopharyngioma" or "craniostenosis, sagittal, with congenital heart disease, mental deficiency, and mandibular ankylosis" or "craniosynostosis-anal anomalies-porokeratosis syndrome" or "craniosynostosis-dandy-walker malformation-hydrocephalus syndrome" or "craniosynostosis-hydrocephalus-arnold-chiari malformation type i-radioulnar synostosis syndrome" or "craniosynostosis-mental retardation syndrome of lin and gettig" or "craniosynostosis-mental retardation-clefting syndrome" or "creatine deficiency syndrome" or "cree mental retardation syndrome" or "cri du chat syndrome" or "cri-du-chat syndrome" or "crigler-najjar syndrome type 1" or "crisponi/cold-induced sweating syndrome 1" or "crome syndrome" or "crouzon syndrome" or "cryptorchidism-arachnodactyly-intellectual disability syndrome" or "ctcf-related neurodevelopmental disorder" or "cubitus valgus with mental retardation and unusual facies" or "curry-jones syndrome" or "cutaneous mastocytosis, conductive hearing loss, and microtia" or "cutis laxa, autosomal recessive, type iia" or "cutis laxa, autosomal recessive, type iiia" or "cutis laxa, autosomal recessive, type iiib" or "cutis verticis gyrata and mental deficiency" or "cutis verticis gyrata, thyroid aplasia, and mental retardation" or "cyclic vomiting syndrome" or "cystathioninuria" or "cysteine peptiduria" or "cystic fibrosis with helicobacter pylori gastritis, megaloblastic anemia, and mental retardation" or "cystic fibrosis-gastritis-megaloblastic anemia syndrome" or "cystinosis" or "d-2-hydroxyglutaric aciduria 1" or "dandy-walker malformation with mental retardation, macrocephaly, myopia, and brachytelephalangy" or "danon disease" or "darier-white disease" or "de sanctis-cacchione syndrome" or "deafness and myopia" or "deafness, autosomal recessive 119" or "deafness, conductive, with malformed external ear" or "deafness, congenital, with total albinism" or "deafness, dystonia, and cerebral hypomyelination" or "deafness, sensorineural, with pituitary dwarfism" or "deafness-enamel hypoplasia-nail defects syndrome" or "deafness-epiphyseal dysplasia-short stature syndrome" or "deafness-genital anomalies-metacarpal and metatarsal synostosis syndrome" or "deafness-intellectual disability syndrome, martin-probst type" or "degcags syndrome" or "delayed speech-facial asymmetry-strabismus-ear lobe creases syndrome" or "dent disease" or "dentinogenesis imperfecta-short stature-hearing loss-intellectual disability syndrome" or "dermatoleukodystrophy" or "dermotrichic syndrome" or "desbuquois dysplasia 1" or "desbuquois dysplasia 2" or "desbuquois syndrome" or "desmosterolosis" or "developmental and speech delay due to sox5 deficiency" or "developmental delay-facial dysmorphism syndrome due to med13l deficiency" or "developmental malformations-deafness-dystonia syndrome" or "dextrocardia with unusual facies and microphthalmia" or "d-glyceric aciduria" or "diabetes insipidus, nephrogenic, 2" or "diabetes insipidus, nephrogenic, x-linked" or "diamond-blackfan anemia 1" or "dibasic amino aciduria i" or "dicarboxylicamino aciduria" or "diets-jongmans syndrome" or "dihydropteridine reductase deficiency" or "dihydropyrimidine dehydrogenase deficiency" or "dihydropyrimidinuria" or "disorder of sex development-intellectual disability syndrome" or "distal 16p11.2 microdeletion syndrome" or "distal 22q11.2 microdeletion syndrome" or "distal 22q11.2 microduplication syndrome" or "distal 7q11.23 microdeletion syndrome" or "distal 7q11.23 microduplication syndrome" or "distal limb deficiencies-micrognathia syndrome" or "distal monosomy 10p" or "distal monosomy 10q" or "distal monosomy 12q" or "distal monosomy 19p13.3" or "distal monosomy 1q" or "distal monosomy 6p" or "distal monosomy 7q36" or "distal monosomy 9p" or "distal trisomy 15q" or "distal trisomy 17q" or "distal trisomy 5q" or "distal xq28 microduplication syndrome" or "dk1-cdg" or "donnai-barrow syndrome" or "doors syndrome" or "dopa-responsive dystonia due to sepiapterin reductase deficiency" or "double outlet right ventricle" or "down syndrome" or "down syndrometrisomy 21, included" or "dpagt1-cdg" or "dpm3-cdg" or "dubowitz syndrome" or "duchenne and becker muscular dystrophy" or "duchenne muscular dystrophy" or "duplication of the pituitary gland" or "dwarfism, low-birth-weight type, with unresponsiveness to growth hormone" or "dwarfism, mental retardation, and eye abnormality" or "dyggve-melchior-clausen disease" or "dyrk1a-related intellectual disability syndrome" or "dysautonomia-like disorder" or "dysequilibrium syndrome" or "dyskinesia, seizures, and intellectual developmental disorder" or "dysmorphism-cleft palate-loose skin syndrome" or "dysmorphism-short stature-deafness-disorder of sex development syndrome" or "dysmyelination with jaundice" or "dysosteosclerosis" or "dysspondyloenchondromatosis" or "dystonia 16" or "dystonia 30" or "dystonia, dopa-responsive, due to sepiapterin reductase deficiency" or "dystonia, juvenile-onset" or "dystonia-parkinsonism-hypermanganesemia syndrome" or "early infantile epileptic encephalopathy" or "early-onset autosomal dominant alzheimer disease" or "early-onset epileptic encephalopathy and intellectual disability due to grin2a mutation" or "early-onset epileptic encephalopathy-cortical blindness-intellectual disability-facial dysmorphism syndrome" or "early-onset parkinsonism-intellectual disability syndrome" or "early-onset progressive diffuse brain atrophy-microcephaly-muscle weakness-optic atrophy syndrome" or "early-onset progressive encephalopathy-spastic ataxia-distal spinal muscular atrophy syndrome" or "early-onset seizures-distal limb anomalies-facial dysmorphism-global developmental delay syndrome" or "early-onset spastic ataxia-myoclonic epilepsy-neuropathy syndrome" or "early-onset x-linked optic atrophy" or "ear-patella-short stature syndrome" or "east syndrome" or "ectodermal dysplasia with mental retardation and syndactyly" or "ectodermal dysplasia, hypohidrotic, with hypothyroidism and agenesisof the corpus callosum" or "ectodermal dysplasia-blindness syndrome" or "ectodermal dysplasia-intellectual disability-central nervous system malformation syndrome" or "ectrodactyly, ectodermal dysplasia, and cleft lip/palate syndrome 3" or "ectrodactyly, ectodermal dysplasia, and cleft lip/palate syndrome1" or "edinburgh malformation syndrome" or "eec syndrome" or "ehlers-danlos syndrome, beasley-cohen type" or "ehlers-danlos syndrome, musculocontractural type 1" or "elejalde disease" or "ellis van creveld syndrome" or "ellis-van creveld syndrome" or "elsahy-waters syndrome" or "emanuel syndrome" or "encephalocraniocutaneous lipomatosis" or "encephalomalacia, multilocular" or "encephalopathy due to sulfite oxidase deficiency" or "encephalopathy with intracranial calcification, growth hormone deficiency, microcephaly, and retinal degeneration" or "encephalopathy, acute, infection-induced (herpes-specific), susceptibility to, 2" or "encephalopathy, ethylmalonic" or "encephalopathy, neonatal severe, due to mecp2 mutations" or "encephalopathy, progressive, early-onset, with brain atrophy and thin corpus callosum" or "encephalopathy, progressive, with amyotrophy and optic atrophy" or "enlarged parietal foramina" or "epidermolysis bullosa, late-onset localized junctional, with mental retardation" or "epilepsy, early-onset, vitamin b6-dependent" or "epilepsy, familial adult myoclonic, 1" or "epilepsy, familial adult myoclonic, 2" or "epilepsy, familial focal, with variable foci" or "epilepsy, familial temporal lobe, 5" or "epilepsy, focal, with speech disorder" or "epilepsy, hearing loss, and mental retardation syndrome" or "epilepsy, idiopathic generalized, susceptibility to, 12" or "epilepsy, idiopathic generalized, susceptibility to, 18" or "epilepsy, nocturnal frontal lobe, 5" or "epilepsy, nocturnal frontal lobe, type 1" or "epilepsy, photogenic, with spastic diplegia and mental retardation" or "epilepsy, progressive myoclonic 1a (unverricht and lundborg)" or "epilepsy, progressive myoclonic 3" or "epilepsy, progressive myoclonic, 11" or "epilepsy, progressive myoclonic, 8" or "epilepsy-microcephaly-skeletal dysplasia syndrome" or "epilepsy-telangiectasia" or "early infantile epileptic encephalopathy").ti,kf,ab. |
| **I, J, K** | ("epileptic encephalopathy, infantile or early childhood, 1" or "epileptic encephalopathy, infantile or early childhood, 2" or "epileptic encephalopathy, infantile or early childhood, 3" or "epiphyseal dysplasia-hearing loss-dysmorphism syndrome" or "ermine phenotype" or "erythrokeratodermia variabilis" or "ethylmalonic encephalopathy" or "extrasystoles, multiform ventricular, with short stature, hyperpigmentationand microcephaly" or "facial abnormalities, kyphoscoliosis, and mental retardation" or "facial dysmorphism-developmental delay-behavioral abnormalities syndrome due to 10p11.21p12.31 microdeletion" or "facial dysmorphism-developmental delay-behavioral abnormalities syndrome due to wac point mutation" or "facial dysmorphism-macrocephaly-myopia-dandy-walker malformation syndrome" or "facial dysmorphism-shawl scrotum-joint laxity syndrome" or "faciocardiomelic syndrome" or "faciocardiorenal syndrome" or "faciodigitogenital syndrome, autosomal recessive" or "facioscapulohumeral muscular dystrophy 1" or "fallot complex with severe mental and growth retardation" or "fallot complex-intellectual disability-growth delay syndrome" or "familial acute necrotizing encephalopathy" or "familial adenomatous polyposis due to 5q22.2 microdeletion" or "familial congenital mirror movements" or "familial exudative vitreoretinopathy" or "familial focal epilepsy with variable foci" or "familial glucocorticoid deficiency" or "familial infantile bilateral striatal necrosis" or "familial infantile myoclonic epilepsy" or "familial lambdoid synostosis" or "familial multiple nevi flammei" or "familial or sporadic hemiplegic migraine" or "familial paroxysmal ataxia" or "familial primary hypomagnesemia with normocalciuria and normocalcemia" or "familial scaphocephaly syndrome, mcgillivray type" or "familial thyroid dyshormonogenesis" or "fanconi anemia" or "farber disease" or "farber lipogranulomatosis" or "fatal infantile lactic acidosis with methylmalonic aciduria" or "fatty acyl-coa reductase 1 deficiency" or "fbln1-related developmental delay-central nervous system anomaly-syndactyly syndrome" or "feingold syndrome" or "female restricted epilepsy with intellectual disability" or "fetal alcohol syndrome" or "fetal iodine deficiency disorder" or "fetal iodine syndrome" or "fetal trimethadione syndrome" or "fg syndrome 4" or "fg syndrome type 1" or "fibrodysplasia ossificans progressiva" or "congenital fibrosis of extraocular muscles" or "fibular hemimelia" or "filippi syndrome" or "floating-harbor syndrome" or "focal cortical dysplasia of taylor" or "focal dermal hypoplasia" or "focal segmental glomerulosclerosis and neurodevelopmental syndrome" or "folate malabsorption, hereditary" or "formiminoglutamic aciduria" or "formiminotransferase deficiency" or "fountain syndrome" or "foxg1 syndrome due to 14q12 microdeletion" or "fragile x mental retardation syndrome" or "fragile x syndrome" or "fraser syndrome" or "fraxe intellectual disability" or "free sialic acid storage disease" or "fried syndrome" or "frontometaphyseal dysplasia" or "frontonasal dysplasia" or "frontoocular syndrome" or "fructose intolerance, hereditary" or "fructose-1,6-bisphosphatase deficiency" or "fryns macrocephaly" or "fryns syndrome" or "fryns-smeets-thiry syndrome" or "fucosidosis" or "fumarase deficiency" or "gabriele-de vries syndrome" or "galactokinase deficiency" or "galactose epimerase deficiency" or "galactosemia" or "galactosialidosis" or "galloway-mowat syndrome 1" or "galloway-mowat syndrome 2" or "galloway-mowat syndrome 3" or "galloway-mowat syndrome 7" or "gapo syndrome" or "gardner syndrome" or "gaucher disease-ophthalmoplegia-cardiovascular calcification syndrome" or "gaze palsy, familial horizontal, with progressive scoliosis" or "gemignani syndrome" or "generalized epilepsy with febrile seizures plus, type 10" or "generalized epilepsy-paroxysmal dyskinesia syndrome" or "genitopatellar syndrome" or "german syndrome" or "geroderma osteodysplastica" or "geroderma osteodysplasticum" or "giant axonal neuropathy" or "gillespie syndrome" or "glass syndrome" or "global developmental delay, absent or hypoplastic corpus callosum, and dysmorphic facies" or "global developmental delay-neuro-ophthalmological abnormalities-seizures-intellectual disability syndrome" or "global developmental delay-visual anomalies-progressive cerebellar atrophy-truncal hypotonia syndrome" or "glut1 deficiency syndrome 1" or "glutamate-cysteine ligase deficiency" or "glutathione synthetase deficiency" or "glutathionuria" or "glycerol kinase deficiency" or "glycine encephalopathy" or "glycogen storage disease due to acid maltase deficiency, infantile onset" or "glycogen storage disease due to aldolase a deficiency" or "glycogen storage disease due to glycogen debranching enzyme deficiency" or "glycogen storage disease due to lamp-2 deficiency" or "glycogen storage disease due to liver phosphorylase kinase deficiency" or "glycogen storage disease due to phosphoglycerate kinase 1 deficiency" or "glycogen storage disease xii" or "glycosylphosphatidylinositol biosynthesis defect 11" or "glycosylphosphatidylinositol biosynthesis defect 15" or "glycosylphosphatidylinositol biosynthesis defect 16" or "gm1 gangliosidosis type 1" or "gm1-gangliosidosis, type i" or "gm1-gangliosidosis, type iii" or "gmppb-related limb-girdle muscular dystrophy r19" or "gms syndrome" or "gnb5-related intellectual disability-cardiac arrhythmia syndrome" or "goldberg-shprintzen megacolon syndrome" or "goldberg-shprintzen syndrome" or "gombo syndrome" or "g mez-l pez-hern ndez syndrome" or "gonadal dysgenesis, xy type" or "gorlin syndrome" or "gorlin-chaudhry-moss syndrome" or "grange syndrome" or "greig cephalopolysyndactyly syndrome" or "griscelli syndrome" or "growth delay due to insulin-like growth factor i resistance" or "growth delay due to insulin-like growth factor type 1 deficiency" or "growth delay-intellectual disability-hepatopathy syndrome" or "growth failure, microcephaly, mental retardation, cataracts, largejoint contractures, osteoporosis, cortical dysplasia, and cerebellaratrophy" or "growth hormone insensitivity syndrome" or "growth retardation, deafness, femoral epiphyseal dysplasia, and lacrimal duct obstruction" or "growth retardation, impaired intellectual development, hypotonia, and hepatopathy" or "guanidinoacetate methyltransferase deficiency" or "gurrieri syndrome" or "h syndrome" or "haddad syndrome" or "hadziselimovic syndrome" or "hair defect with photosensitivity and mental retardation" or "hallermann-streiff syndrome" or "hall-riggs mental retardation syndrome" or "hall-riggs syndrome" or "hamel cerebro-palato-cardiac syndrome" or "hand and foot deformity with flat facies" or "harel-yoon syndrome" or "harrod syndrome" or "hartnup disease" or "heart-hand syndrome type 2" or "helsmoortel-van der aa syndrome" or "hemifacial microsomia" or "hemihyperplasia, isolated" or "hemimegalencephaly" or "hemolytic anemia, nonspherocytic, due to glucose phosphate isomerasedeficiency" or "hengel-maroofian-schols syndrome" or "hennekam lymphangiectasia-lymphedema syndrome" or "hennekam syndrome" or "hennekam-beemer syndrome" or "hepatic fibrosis-renal cysts-intellectual disability syndrome" or "hereditary bullous dystrophy, macular type" or "hereditary cryohydrocytosis with reduced stomatin" or "hereditary hyperekplexia" or "hereditary methemoglobinemia" or "hereditary sensory and autonomic neuropathy due to tecpr2 mutation" or "hereditary sensory and autonomic neuropathy type 4" or "hereditary sensory and autonomic neuropathy type 5" or "hermansky-pudlak syndrome 2" or "heterotopia, periventricular, x-linked dominant" or "hiatt-neu-cooper neurodevelopmental syndrome" or "hidrotic ectodermal dysplasia, halal type" or "hirschsprung disease" or "hirschsprung disease-deafness-polydactyly syndrome" or "hirsutism, skeletal dysplasia, and mental retardation" or "histidinemia" or "histidinuria due to a renal tubular defect" or "histidinuria-renal tubular defect syndrome" or "hnf1b-related autosomal dominant tubulointerstitial kidney disease" or "holoprosencephaly 1" or "holoprosencephaly 13, x-linked" or "holoprosencephaly 2" or "holoprosencephaly 3" or "holoprosencephaly 5" or "holoprosencephaly, recurrent infections, and monocytosis" or "homocarnosinosis" or "homocystinuria due to cystathionine beta-synthase deficiency" or "homocystinuria due to methylene tetrahydrofolate reductase deficiency" or "homocystinuria-megaloblastic anemia, cbl e type" or "homocystinuria-megaloblastic anemia, cblg complementation type" or "hooft disease" or "hoyeraal-hreidarsson syndrome" or "hsd10 disease" or "hsd10 mitochondrial disease" or "hunter-mcalpine craniosynostosis syndrome" or "hurler syndrome" or "hutterite cerebroosteonephrodysplasia syndrome" or "hydrocephalus due to congenital stenosis of aqueduct of sylvius" or "hydrocephalus with stenosis of the aqueduct of sylvius" or "hydrocephalus, congenital, 2" or "hydrocephalus, nonsyndromic, autosomal recessive 1" or "hydrocephalus, skeletal anomalies, and mental disturbance" or "hydrocephalus-costovertebral dysplasia-sprengel anomaly syndrome" or "hydrocephalus-obesity-hypogonadism syndrome" or "hydrocephaly-cerebellar agenesis syndrome" or "hydroxykynureninuria" or "hydroxylysinuria" or "hydroxyprolinemia" or "hypercalcemia, infantile, 1" or "hyperinsulinemic hypoglycemia, familial, 1" or "hyperinsulinemic hypoglycemia, familial, 3" or "hyperinsulinemic hypoglycemia, familial, 4" or "hyperinsulinemic hypoglycemia, familial, 6" or "hyperinsulinism due to hnf4a deficiency" or "hyperinsulinism-hyperammonemia syndrome" or "hyperleucine-isoleucinemia" or "hyperlysinemia" or "hyperlysinuria with hyperammonemia" or "hypermanganesemia with dystonia 2" or "hypermethioninemia with s-adenosylhomocysteine hydrolase deficiency" or "hyperornithinemia-hyperammonemia-homocitrullinuria syndrome" or "hyperphenylalaninemia" or "hyperphosphatasia with mental retardation" or "hyperphosphatasia-intellectual disability syndrome" or "hyperprolinemia type 2" or "hyperprolinemia, type i" or "hyperprolinemia, type ii" or "hypertelorism and tetralogy of fallot" or "hypertelorism-microtia-facial clefting syndrome" or "hyperthyroidism, nonautoimmune" or "hypertrichosis cubiti" or "hypertrichosis-acromegaloid facial appearance syndrome" or "hypertrichotic osteochondrodysplasia" or "hypertryptophanemia" or "hyperuricemia, infantile, with abnormal behavior and normal hypoxanthineguanine phosphoribosyltransferase" or "hypo- and hypermelanotic cutaneous macules-retarded growth-intellectual disability syndrome" or "hypochondroplasia" or "hypoglossia-hypodactyly syndrome" or "hypoglycemia of infancy, leucine-sensitive" or "hypogonadism with low-grade mental deficiency and microcephaly" or "hypogonadism-mitral valve prolapse-intellectual disability syndrome" or "hypogonadotropic hypogonadism 10" or "hypogonadotropic hypogonadism 2 " or "hypohidrosis-enamel hypoplasia-palmoplantar keratoderma-intellectual disability syndrome" or "hypomagnesemia 4, renal" or "hypomagnesemia, seizures, and mental retardation" or "hypomelanosis of ito" or "hypomyelination with brainstem and spinal cord involvement and legspasticity" or "hypomyelination-congenital cataract syndrome" or "hypoparathyroidism-retardation-dysmorphism syndrome" or "hypospadias-intellectual disability, goldblatt type syndrome" or "hypospadias-mental retardation syndrome" or "hypotonia, ataxia, and delayed development syndrome" or "hypotonia, ataxia, developmental delay, and tooth enamel defect syndrome" or "hypotonia, hypoventilation, impaired intellectual development, dysautonomia, epilepsy, and eye abnormalities" or "hypotonia, infantile, with psychomotor retardation and characteristic facies 2" or "hypotonia-cystinuria syndrome" or "hypotonia-speech impairment-severe cognitive delay syndrome" or "hypotrichosis-intellectual disability, lopes type" or "hypoxanthine guanine phosphoribosyltransferase partial deficiency" or "icf syndrome" or "ichthyosiform erythroderma, corneal involvement, and deafness" or "ichthyosis and male hypogonadism" or "ichthyosis follicularis-alopecia-photophobia syndrome" or "ichthyosis with alopecia, eclabion, ectropion, and mental retardation" or "ichthyosis, congenital, autosomal recessive 2" or "ichthyosis, mental retardation, dwarfism, and renal impairment" or "ichthyosis, spastic quadriplegia, and mental retardation" or "ichthyosis, split hairs, and amino aciduria" or "ichthyosis-alopecia-eclabion-ectropion-intellectual disability syndrome" or "ichthyosis-intellectual disability-dwarfism-renal impairment syndrome" or "ichthyosis-mental retardation syndrome with large keratohyalin granules in the skin" or "ifap syndrome" or "imagawa-matsumoto syndrome" or "iminoglycinuria" or "immunodeficiency 23" or "immunodeficiency 47" or "immunodeficiency 49" or "immunodeficiency due to purine nucleoside phosphorylase deficiency" or "immunodeficiency, developmental delay, and hypohomocysteinemia" or "immunodeficiency-centromeric instability-facial anomalies syndrome" or "immunodeficiency-centromeric instability-facial anomalies syndrome 3" or "immunodeficiency-centromeric instability-facial anomalies syndrome 4" or "immunodeficiency-centromeric instability-facial anomalies syndrome 2" or "immunoskeletal dysplasia with neurodevelopmental abnormalities" or "inclusion body myopathy with paget disease of bone and frontotemporal dementia" or "incontinentia pigmenti" or "indolylacroyl glycinuria with mental retardation" or "infantile cerebellar-retinal degeneration" or "infantile choroidocerebral calcification syndrome" or "infantile multisystem neurologic-endocrine-pancreatic disease" or "insensitivity to pain, congenital, with anhidrosis" or "insulin-like growth factor i deficiency" or "insulin-like growth factor i, resistance to" or "intellectual developmental disorder" or "intellectual disability syndrome due to a dyrk1a point mutation" or "intellectual disability, buenos-aires type" or "intellectual disability, wolff type" or "intellectual disability-alacrima-achalasia syndrome" or "intellectual disability-autism-speech apraxia-craniofacial dysmorphism syndrome" or "intellectual disability-balding-patella luxation-acromicria syndrome" or "intellectual disability-brachydactyly-pierre robin syndrome" or "intellectual disability-cardiac anomalies-short stature-joint laxity syndrome" or "intellectual disability-cataracts-calcified pinnae-myopathy syndrome" or "intellectual disability-cataracts-kyphosis syndrome" or "intellectual disability-coarse face-macrocephaly-cerebellar hypotrophy syndrome" or "intellectual disability-craniofacial dysmorphism-cryptorchidism syndrome" or "intellectual disability-developmental delay-contractures syndrome" or "intellectual disability-dysmorphism-hypogonadism-diabetes mellitus syndrome" or "intellectual disability-epilepsy-extrapyramidal syndrome" or "intellectual disability-expressive aphasia-facial dysmorphism syndrome" or "intellectual disability-facial dysmorphism syndrome due to setd5 haploinsufficiency" or "intellectual disability-facial dysmorphism-hand anomalies syndrome" or "intellectual disability-hyperkinetic movement-truncal ataxia syndrome" or "intellectual disability-hypoplastic corpus callosum-preauricular tag syndrome" or "intellectual disability-macrocephaly-hypotonia-behavioral abnormalities syndrome" or "intellectual disability-muscle weakness-short stature-facial dysmorphism syndrome" or "intellectual disability-myopathy-short stature-endocrine defect syndrome" or "intellectual disability-obesity-brain malformations-facial dysmorphism syndrome" or "intellectual disability-obesity-prognathism-eye and skin anomalies syndrome" or "intellectual disability-polydactyly-uncombable hair syndrome" or "intellectual disability-seizures-abnormal gait-facial dysmorphism syndrome" or "intellectual disability-seizures-hypophosphatasia-ophthalmic-skeletal anomalies syndrome" or "intellectual disability-seizures-macrocephaly-obesity syndrome" or "intellectual disability-severe speech delay-mild dysmorphism syndrome" or "intellectual disability-short stature-hypertelorism syndrome" or "intellectual disability-spasticity-ectrodactyly syndrome" or "intellectual disability-strabismus syndrome" or "irida syndrome" or "isolated brachycephaly" or "isolated focal cortical dysplasia" or "isolated glycerol kinase deficiency" or "isolated hemihyperplasia" or "isolated lissencephaly type 1 without known genetic defects" or "isolated oxycephaly" or "isolated permanent neonatal diabetes mellitus" or "isolated plagiocephaly" or "jaberi-elahi syndrome" or "jacobsen syndrome" or "japanese encephalitis" or "jawad syndrome" or "jeavons syndrome" or "johanson-blizzard syndrome" or "johnson neuroectodermal syndrome" or "joubert syndrome" or "juberg-hayward syndrome" or "jung syndrome" or "juvenile polyposis of infancy" or "juvenile polyposis syndrome" or "juvenile sialidosis type 2" or "juvenile-onset diabetes mellitus-central and peripheral neurodegeneration syndrome" or "kabuki syndrome").ti,kf,ab. |
| **M, N, O, P, Q, R** | ("kagami-ogata syndrome" or "kahrizi syndrome" or "kallmann syndrome-heart disease syndrome" or "kanzaki disease" or "kapur-toriello syndrome" or "kaufman oculocerebrofacial syndrome" or "kbg syndrome" or "kcnq2-related epileptic encephalopathy" or "keipert syndrome" or "kennerknecht syndrome" or "keppen-lubinsky syndrome" or "keutel syndrome" or "kifafa seizure disorder" or "kilquist syndrome" or "kinsship syndrome" or "kleefstra syndrome" or "klippel-tr naunay syndrome" or "klippel-trenaunay-weber syndrome" or "kohlschutter-tonz syndrome" or "koolen-de vries syndrome" or "kufor-rakeb syndrome" or "kyphoscoliosis-lateral tongue atrophy-hereditary spastic paraplegia syndrome" or "l1 syndrome" or "l-2-hydroxyglutaric aciduria" or "lactic aciduria due to d-lactic acid" or "lambert syndrome" or "lamb-shaffer syndrome" or "laminin subunit alpha 2-related congenital muscular dystrophy" or "laron syndrome" or "larsen syndrome" or "laryngeal abductor paralysis" or "lathosterolosis" or "laurence-moon syndrome" or "laurin-sandrow syndrome" or "lead poisoning" or "leber congenital amaurosis" or "leber optic atrophy and dystonia" or "leigh syndrome" or "lelis syndrome" or "lennox-gastaut syndrome" or "lentiginosis, centrofacial neurodysraphic" or "lenz-majewski hyperostotic dwarfism" or "leopard syndrome 1" or "leopard syndrome 3" or "leprechaunism" or "lesch-nyhan phenotype with normal hgprt" or "lesch-nyhan syndrome" or "lessel-kreienkamp syndrome" or "lethal ataxia with deafness and optic atrophy" or "leukocyte adhesion deficiency" or "leukocyte adhesion deficiency type ii" or "leukodystrophy and acquired microcephaly" or "leukodystrophy, hypomyelinating, 11" or "leukodystrophy, hypomyelinating, 12" or "leukodystrophy, hypomyelinating, 14" or "leukodystrophy, hypomyelinating, 16" or "leukodystrophy, hypomyelinating, 17" or "leukodystrophy, hypomyelinating, 22" or "leukodystrophy, hypomyelinating, 4" or "leukodystrophy, hypomyelinating, 5" or "leukodystrophy, hypomyelinating, 6" or "leukodystrophy, hypomyelinating, 8" or "leukodystrophy, hypomyelinating, 9" or "leukodystrophy, progressive, early childhood-onset" or "leukoencephalopathy with bilateral anterior temporal lobe cysts" or "leukoencephalopathy with brain stem and spinal cord involvement-high lactate syndrome" or "leukoencephalopathy with metaphyseal chondrodysplasia" or "leukoencephalopathy-spondyloepimetaphyseal dysplasia syndrome" or "leukomelanoderma, infantilism, mental retardation, hypodontia, hypotrichosis" or "leukomelanoderma-infantilism-intellectual disability-hypodontia-hypotrichosis syndrome" or "liberfarb syndrome" or "li-campeau syndrome" or "lig4 syndrome" or "limb defects, distal transverse, with mental retardation and spasticity" or "linear nevus sebaceus syndrome" or "lipodystrophy, congenital generalized, type 2" or "lipodystrophy, generalized, with mental retardation, deafness, short stature, and slender bones" or "lipodystrophy-intellectual disability-deafness syndrome" or "lissencephaly 1" or "lissencephaly 10" or "lissencephaly 3" or "lissencephaly 4" or "lissencephaly 5" or "lissencephaly 8" or "lissencephaly due to lis1 mutation" or "lissencephaly syndrome, norman-roberts type" or "lissencephaly, x-linked, 1" or "listeriosis" or "lobar holoprosencephaly" or "loeys-dietz syndrome 1" or "loeys-dietz syndrome 2" or "long chain 3-hydroxyacyl-coa dehydrogenase deficiency" or "lopes-maciel-rodan syndrome" or "lowe syndrome" or "lowry-maclean syndrome" or "lowry-wood syndrome" or "lujan-fryns syndrome" or "luo-schoch-yamamoto syndrome" or "luscan-lumish syndrome" or "lysine malabsorption syndrome" or "lysinuric protein intolerance" or "macrocephaly and epileptic encephalopathy" or "macrocephaly, dysmorphic facies, and psychomotor retardation" or "macrocephaly/autism syndrome" or "macrocephaly/megalencephaly syndrome, autosomal recessive" or "macrocephaly-developmental delay syndrome" or "macrocephaly-intellectual disability-left ventricular non compaction syndrome" or "macrocephaly-intellectual disability-neurodevelopmental disorder-small thorax syndrome" or "macrocephaly-spastic paraplegia-dysmorphism syndrome" or "macrothrombocytopenia-lymphedema-developmental delay-facial dysmorphism-camptodactyly syndrome" or "magel2-related prader-willi-like syndrome" or "malan overgrowth syndrome" or "male hypergonadotropic hypogonadism-intellectual disability-skeletal anomalies syndrome" or "male pseudohermaphroditism/mental retardation syndrome, verloes type" or "malonyl-coa decarboxylase deficiency" or "malouf syndrome" or "man1b1-cdg" or "mandibulofacial dysostosis with mental retardation" or "mandibulofacial dysostosis-microcephaly syndrome" or "mannosidosis, beta a, lysosomal" or "maple syrup urine disease" or "marden-walker syndrome" or "marfanoid habitus with microcephaly and glomerulonephritis" or "marfanoid mental retardation syndrome, autosomal" or "marinesco-sjogren syndrome" or "marinesco-sj gren syndrome" or "marshall-smith syndrome" or "martsolf syndrome 1" or "martsolf syndrome 2" or "masa syndrome" or "maternal phenylketonuria" or "maternal uniparental disomy of chromosome 4" or "maternal uniparental disomy of chromosome 6" or "maternal uniparental disomy of chromosome x" or "matthew-wood syndrome" or "mcdonough syndrome" or "mckusick-kaufman syndrome" or "meckel syndrome 13" or "mednik syndrome" or "megalencephalic leukoencephalopathy with subcortical cysts 1" or "megalencephalic leukoencephalopathy with subcortical cysts 2a" or "megalencephalic leukoencephalopathy with subcortical cysts 2b" or "megalencephaly" or "megalocornea-intellectual disability syndrome" or "megalocornea-mental retardation syndrome" or "mehmo syndrome" or "meier-gorlin syndrome 1" or "meier-gorlin syndrome 6" or "mend syndrome" or "menke-hennekam syndrome 1" or "menke-hennekam syndrome 2" or "menkes disease" or "mental and growth retardation with amblyopia" or "mental retardation and distinctive facial features" or "mental retardation and microcephaly with pontine and cerebellar hypoplasia" or "mental retardation associated with psoriasis" or "mental retardation syndrome" or "mercaptolactate-cysteine disulfiduria" or "mesangial sclerosis, diffuse renal, with ocular abnormalities" or "mesoaxial hexadactyly and cardiac malformation" or "mesomelic dysplasia, nievergelt type" or "mesomelic dysplasia, savarirayan type" or "metabolic encephalomyopathic crises, recurrent, with rhabdomyolysis, cardiac arrhythmias, and neurodegeneration" or "metachromatic leukodystrophy" or "metaphyseal acroscyphodysplasia" or "metaphyseal dysostosis, mental retardation, and conductive deafness" or "metaphyseal dysostosis-intellectual disability-conductive deafness syndrome" or "methemoglobinemia due to deficiency of methemoglobin reductase" or "methionine adenosyltransferase i/iii deficiency" or "methionine malabsorption syndrome" or "methylcobalamin deficiency type cble" or "methylmalonic acidemia and homocysteinemia, cblx type" or "methylmalonic acidemia with homocystinuria" or "methylmalonic aciduria and homocystinuria, cblc type" or "methylmalonic aciduria and homocystinuria, cbld type" or "mevalonic aciduria" or "micro syndrome" or "microbrachycephaly-ptosis-cleft lip syndrome" or "microcephalic cortical malformations-short stature due to rttn deficiency" or "microcephalic osteodysplastic primordial dwarfism" or "microcephalic primordial dwarfism, dauber type" or "microcephalic primordial dwarfism, montreal type" or "microcephalic primordial dwarfism, toriello type" or "autosomal recessive primary microcephaly " or "autosomal dominant primary microcephaly " or "primary autosomal recessive microcephaly " or "autosomal recessive microcephaly and chorioretinopathy" or "microcephaly with cervical spine fusion anomalies" or "microcephaly, cerebellar hypoplasia, and cardiac conduction defect syndrome" or "microcephaly, congenital cataract, and psoriasiform dermatitis" or "microcephaly, developmental delay, and brittle hair syndrome" or "microcephaly, epilepsy, and diabetes syndrome" or "microcephaly, seizures, and developmental delay" or "microcephaly, seizures, spasticity, and brain calcifications" or "microcephaly, short stature, and impaired glucose metabolism" or "microcephaly, short stature, and limb abnormalities" or "microcephaly, short stature, and polymicrogyria" or "microcephaly-brachydactyly-kyphoscoliosis syndrome" or "microcephaly-cardiomyopathy" or "microcephaly-cervical spine fusion anomalies syndrome" or "microcephaly-cleft palate-abnormal retinal pigmentation syndrome" or "microcephaly-corpus callosum and cerebellar vermis hypoplasia-facial dysmorphism-intellectual disability syndrome" or "microcephaly-corpus callosum hypoplasia-intellectual disability-facial dysmorphism syndrome" or "microcephaly-deafness syndrome" or "microcephaly-deafness-intellectual disability syndrome" or "microcephaly-glomerulonephritis-marfanoid habitus syndrome" or "microcephaly-intellectual disability-sensorineural hearing loss-epilepsy-abnormal muscle tone syndrome" or "microcephaly-lymphedema-chorioretinopathy syndrome" or "microcephaly-microcornea syndrome, seemanova type" or "microcephaly-seizures-intellectual disability-heart disease syndrome" or "microcephaly-thin corpus callosum-intellectual disability syndrome" or "microduplication xp11.22p11.23 syndrome" or "microform holoprosencephaly" or "micrognathia-recurrent infections-behavioral abnormalities-mild intellectual disability syndrome" or "microhydranencephaly" or "microlissencephaly" or "microphthalmia with limb anomalies" or "microphthalmia with linear skin defects syndrome" or "microphthalmia, isolated, with coloboma 9" or "microphthalmia, lenz type" or "syndromic microphthalmia" or "microphthalmia/coloboma and skeletal dysplasia syndrome" or "microphthalmia-ankyloblepharon-intellectual disability syndrome" or "microtriplication 11q24.1" or "midface hypoplasia, hearing impairment, elliptocytosis, and nephrocalcinosis" or "midline interhemispheric variant of holoprosencephaly" or "mietens syndrome" or "migraine, familial hemiplegic, 2" or "miller-dieker lissencephaly syndrome" or "mirror movements 1" or "mitochondrial complex i deficiency, nuclear type 16" or "mitochondrial complex iii deficiency, nuclear type 1" or "mitochondrial complex iii deficiency, nuclear type 4" or "mitochondrial complex iii deficiency, nuclear type 8" or "mitochondrial complex iv deficiency" or "mitochondrial complex iv deficiency, nuclear type 17" or "mitochondrial complex iv deficiency, nuclear type 8" or "mitochondrial complex v (atp synthase) deficiency, nuclear type 3" or "mitochondrial dna depletion syndrome" or "mitochondrial dna-related progressive external ophthalmoplegia" or "mitochondrial myopathy and sideroblastic anemia" or "mitochondrial myopathy-cerebellar ataxia-pigmentary retinopathy syndrome" or "mitochondrial neurogastrointestinal encephalomyopathy" or "mmep syndrome" or "moebius syndrome" or "molybdenum cofactor deficiency, complementation group a" or "momo syndrome" or "monilethrix" or "monocarboxylate transporter 1 deficiency" or "monosomy 13q14" or "monosomy 13q34" or "monosomy 18p" or "monosomy 18q" or "monosomy 22" or "monosomy 22q13.3" or "monosomy 5p" or "monosomy 9p" or "monosomy 9q22.3" or "morbid obesity and spermatogenic failure" or "morm syndrome" or "morquio syndrome c" or "mosaic trisomy 1" or "mosaic trisomy 14" or "mosaic trisomy 8" or "mosaic trisomy 9" or "mosaic variegated aneuploidy syndrome" or "mowat-wilson syndrome" or "moyamoya disease" or "moynahan syndrome" or "mpdu1-cdg" or "mucolipidosis iii" or "mucolipidosis iv" or "mucolipidosis type iv" or "mucopolysaccharidosis" or "muenke syndrome" or "mulibrey nanism" or "multicentric osteolysis-nodulosis-arthropathy spectrum" or "multicore myopathy with mental retardation, short stature, and hypogonadotropichypogonadism" or "multiple benign circumferential skin creases on limbs" or "multiple congenital anomalies-hypotonia-seizures syndrome 1" or "multiple congenital anomalies-neurodevelopmental syndrome, x-linked" or "multiple epiphyseal dysplasia, al-gazali type" or "multiple mitochondrial dysfunctions syndrome 6" or "multiple sulfatase deficiency" or "muscle-eye-brain disease with bilateral multicystic leucodystrophy" or "muscular dystrophy, congenital, due to integrin alpha-7 deficiency" or "muscular dystrophy, congenital, megaconial type" or "muscular dystrophy, congenital, merosin deficient or partially deficient" or "muscular dystrophy, congenital, with cataracts and intellectual disability" or "muscular dystrophy, limb-girdle, autosomal recessive 18" or "muscular dystrophy, limb-girdle, autosomal recessive 27" or "congenital muscular dystrophy-dystroglycanopathy" or "muscular dystrophy-dystroglycanopathy (limb-girdle), type c" or "mycophenolate mofetil embryopathy" or "myh7-related late-onset scapuloperoneal muscular dystrophy" or "myhre syndrome" or "myoclonic epilepsy of infancy" or "myoclonic-astatic epilepsy" or "myoclonic-atonic epilepsy" or "myopathy and diabetes mellitus" or "myopathy, centronuclear, 2" or "myopathy, congenital, bailey-bloch" or "myopathy, lactic acidosis, and sideroblastic anemia 1" or "myopathy, mitochondrial, and ataxia" or "myotonia permanens" or "myotonia with skeletal abnormalities and mental retardation" or "myotonic dystrophy 1" or "n syndrome" or "nabais sa-de vries syndrome" or "nance-horan syndrome" or "narp syndrome" or "native american myopathy" or "nephrogenic diabetes insipidus-intracranial calcification-facial dysmorphism syndrome" or "nephronophthisis 18" or "nephronophthisis-like nephropathy 1" or "nephrosialidosis" or "nephrotic syndrome, type 8" or "netherton syndrome" or "neuraminidase deficiency" or "neurocutaneous melanocytosis" or "neurodegeneration due to cerebral folate transport deficiency" or "neurodegeneration with brain iron accumulation 2a" or "neurodegeneration with brain iron accumulation 5" or "neurodegeneration, childhood-onset, with brain atrophy" or "neurodevelopmental delay-seizures-ophthalmic anomalies-osteopenia-cerebellar atrophy syndrome" or "neurodevelopmental disorder-craniofacial dysmorphism-cardiac defect-skeletal anomalies syndrome" or "neurodevelopmental, jaw, eye, and digital syndrome" or "neurodevelopmental-craniofacial syndrome with variable renal and cardiac abnormalities" or "neuroectodermal melanolysosomal disease" or "neurofaciodigitorenal syndrome" or "neurofacioskeletal syndrome" or "neurofibromatosis type 1" or "neurofibromatosis, type i" or "neurofibromatosis-noonan syndrome" or "neurologic disease, infantile multisystem, with osseous fragility" or "neurologic, endocrine, and pancreatic disease, multisystem, infantile-onset" or "neuromuscular disease and ocular or auditory anomalies" or "neuroocular syndrome" or "neurooculocardiogenitourinary syndrome" or "neuropathy, hereditary motor and sensory, with deafness, mental retardation,and absent sensory large myelinated fibers" or "neuropathy, hereditary sensory and autonomic, type v" or "neutral lipid storage myopathy" or "neutropenia, severe congenital 3, autosomal recessive" or "nf1-microdeletion syndrome" or "nicolaides-baraitser syndrome" or "niemann-pick disease" or "nijmegen breakage syndrome" or "nizon-isidor syndrome" or "nkx6-2-related autosomal recessive hypomyelinating leukodystrophy" or "non-insulinoma pancreatogenous hypoglycemia syndrome" or "non-progressive cerebellar ataxia with intellectual disability" or "non-progressive predominantly posterior cavitating leukoencephalopathy with peripheral neuropathy" or "non-specific early-onset epileptic encephalopathy" or "non-specific syndromic intellectual disability" or "noonan syndrome" or "norrie disease" or "null syndrome" or "obesity, hyperphagia, and developmental delay" or "occipital horn syndrome" or "ocular anomalies-axonal neuropathy-developmental delay syndrome" or "oculocerebral hypopigmentation syndrome, cross type" or "oculocerebral hypopigmentation syndrome, preus type" or "oculocerebral syndrome with hypopigmentation" or "oculocerebrocutaneous syndrome" or "oculocerebrofacial syndrome, kaufman type" or "oculocerebrorenal syndrome of lowe" or "oculodentodigital dysplasia" or "oculofaciocardiodental syndrome" or "oculopalatocerebral syndrome" or "oculo-palato-cerebral syndrome" or "oculorenocerebellar syndrome" or "ohdo syndrome" or "okamoto syndrome" or "okur-chung neurodevelopmental syndrome" or "oliver syndrome" or "oliver-mcfarlane syndrome" or "ondontochondrodysplasia 2 with hearing loss and diabetes" or "onychodystrophy, osteodystrophy, impaired intellectual development, and seizures syndrome" or "onychotrichodysplasia and neutropenia" or "ophthalmoplegia, progressive, with scrotal tongue and mental deficiency" or "ophthalmoplegia-intellectual disability-lingua scrotalis syndrome" or "opitz gbbb syndrome" or "opitz-kaveggia syndrome" or "optic atrophy 10" or "optic atrophy 11" or "optic atrophy 2" or "optic atrophy-intellectual disability syndrome" or "ornithine transcarbamylase deficiency" or "orofaciodigital syndrome" or "osteogenesis imperfecta-retinopathy-seizures-intellectual disability syndrome" or "osteoglosphonic dysplasia" or "recessive osteolysis syndrome" or "osteopathia striata with cranial sclerosis" or "osteopathia striata-cranial sclerosis syndrome" or "osteopenia and sparse hair" or "osteopetrosis with renal tubular acidosis" or "osteopetrosis, autosomal recessive 3" or "osteoporosis-macrocephaly-blindness-joint hyperlaxity syndrome" or "osteoporosis-pseudoglioma syndrome" or "osteosclerotic metaphyseal dysplasia" or "otofaciocervical syndrome" or "otoonychoperoneal syndrome" or "otopalatodigital syndrome type 1" or "otopalatodigital syndrome type 2" or "otopalatodigital syndrome, type i" or "otopalatodigital syndrome, type ii" or "overgrowth-macrocephaly-facial dysmorphism syndrome" or "pachygyria-intellectual disability-epilepsy syndrome" or "palant cleft palate syndrome" or "pallister w syndrome" or "pallister-hall syndrome" or "pallister-killian syndrome" or "pancreatic hypoplasia-diabetes-congenital heart disease syndrome" or "pantothenate kinase-associated neurodegeneration" or "papillorenal syndrome" or "paraplegia-intellectual disability-hyperkeratosis syndrome" or "paris-trousseau thrombocytopenia" or "parkinson disease 19a, juvenile-onset" or "paroxysmal dystonic choreathetosis with episodic ataxia and spasticity" or "paroxysmal exertion-induced dyskinesia" or "partial deletion of the short arm of chromosome 7" or "partial trisomy/tetrasomy of the short arm of chromosome 9" or "partington syndrome" or "paternal 20q13.2q13.3 microdeletion syndrome" or "paternal uniparental disomy of chromosome x" or "patterson pseudoleprechaunism syndrome" or "pcna-related progressive neurodegenerative photosensitivity syndrome" or "pde4d haploinsufficiency syndrome" or "peho syndrome" or "peho-like syndrome" or "pelger-huet anomaly" or "pelizaeus-merzbacher disease" or "pendred syndrome" or "pentasomy x" or "perioral myoclonia with absences" or "peripheral demyelinating neuropathy, central dysmyelination, waardenburg syndrome, and hirschsprung disease" or "peripheral demyelinating neuropathy-central dysmyelinating leukodystrophy-waardenburg syndrome-hirschsprung disease" or "periventricular heterotopia with microcephaly, autosomal recessive" or "periventricular nodular heterotopia 7" or "periventricular nodular heterotopia 9" or "perlman syndrome" or "peroxisomal acyl-coa oxidase deficiency").ti,kf,ab. |
| **S, T, U, V, W, X, Y, Z** | ("peroxisomal fatty acyl-coa reductase 1 disorder" or "peroxisome biogenesis disorder 14b" or "peroxisome biogenesis disorder 1a (zellweger)" or "peroxisome biogenesis disorder 2a (zellweger)" or "peroxisome biogenesis disorder 2b" or "peroxisome biogenesis disorder 3b" or "peroxisome biogenesis disorder 4b" or "peroxisome biogenesis disorder 5a (zellweger)" or "peroxisome biogenesis disorder 9b" or "peters plus syndrome" or "peters-plus syndrome" or "pettigrew syndrome" or "pfeiffer syndrome" or "pfeiffer-palm-teller syndrome" or "pgm3-cdg" or "phelan-mcdermid syndrome" or "phenobarbital embryopathy" or "phenylketonuria" or "phosphoglycerate dehydrogenase deficiency" or "phosphoglycerate kinase 1 deficiency" or "phosphoribosylpyrophosphate synthetase superactivity" or "phosphoserine phosphatase deficiency" or "piebald trait with neurologic defects" or "piebald trait-neurologic defects syndrome" or "piebaldism" or "pierpont syndrome" or "pilarowski-bjornsson syndrome" or "pitt-hopkins syndrome" or "pitt-hopkins-like syndrome" or "pituitary hormone deficiency, combined, 1" or "pituitary stalk interruption syndrome" or "plaa-associated neurodevelopmental disorder" or "pmm2-cdg" or "poirier-bienvenu neurodevelopmental syndrome" or "polyendocrine-polyneuropathy syndrome" or "polyhydramnios, megalencephaly, and symptomatic epilepsy" or "polymicrogyria due to tubb2b mutation" or "polymicrogyria, bilateral frontoparietal" or "polymicrogyria, bilateral perisylvian" or "polyneuropathy-intellectual disability-acromicria-premature menopause syndrome" or "polyvalvular heart disease syndrome" or "pomgnt2-related limb-girdle muscular dystrophy r24" or "pomt1-related limb-girdle muscular dystrophy r11" or "pontine tegmental cap dysplasia" or "pontocerebellar hypoplasia type 10" or "pontocerebellar hypoplasia type 1a" or "pontocerebellar hypoplasia type 7" or "pontocerebellar hypoplasia, type 11" or "pontocerebellar hypoplasia, type 14" or "pontocerebellar hypoplasia, type 15" or "pontocerebellar hypoplasia, type 2d" or "pontocerebellar hypoplasia, type 2e" or "pontocerebellar hypoplasia, type 2f" or "pontocerebellar hypoplasia, type 8" or "porencephaly" or "porphyria due to ala dehydratase deficiency" or "posterior column ataxia-retinitis pigmentosa syndrome" or "postnatal microcephaly-infantile hypotonia-spastic diplegia-dysarthria-intellectual disability syndrome" or "potocki-lupski syndrome" or "potocki-shaffer syndrome" or "prader-willi habitus, osteopenia, and camptodactyly" or "prader-willi syndrome" or "prader-willi-like syndrome" or "preaxial polydactyly-colobomata-intellectual disability syndrome" or "presynaptic congenital myasthenic syndromes" or "prieto syndrome" or "primary hyperaldosteronism-seizures-neurological abnormalities syndrome" or "primary hypergonadotropic hypogonadism-partial alopecia syndrome" or "primary microcephaly-mild intellectual disability-young-onset diabetes syndrome" or "primary non-essential cutis verticis gyrata" or "primrose syndrome" or "progeria-short stature-pigmented nevi syndrome" or "progeroid facial appearance with hand anomalies" or "progeroid short stature with pigmented nevi" or "progressive epilepsy-intellectual disability syndrome, finnish type" or "progressive essential tremor-speech impairment-facial dysmorphism-intellectual disability-abnormal behavior syndrome" or "progressive external ophthalmoplegia-myopathy-emaciation syndrome" or "progressive myoclonic epilepsy type 1" or "progressive myoclonic epilepsy type 3" or "progressive spondyloepimetaphyseal dysplasia-short stature-short fourth metatarsals-intellectual disability syndrome" or "prolactin deficiency with obesity and enlarged testes" or "prolidase deficiency" or "propionic acidemia" or "proteasome-associated autoinflammatory syndrome 1 and digenic forms" or "proteus syndrome" or "proteus-like syndrome" or "proximal 16p11.2 microdeletion syndrome" or "proximal 16p11.2 microduplication syndrome" or "proximal renal tubular acidosis" or "proximal xq28 duplication syndrome" or "prune belly syndrome with pulmonic stenosis, mental retardation, and deafness" or "pseudoaminopterin syndrome" or "pseudohypoparathyroidism type 1a" or "pseudohypoparathyroidism type 1c" or "pseudohypoparathyroidism with albright hereditary osteodystrophy" or "pseudoleprechaunism syndrome, patterson type" or "pseudoprogeria syndrome" or "pseudopseudohypoparathyroidism" or "pseudouridinuria and mental defect" or "pten hamartoma tumor syndrome" or "pterygia, mental retardation, and distinctive craniofacial features" or "pterygium colli and mental retardation with facial and digital anomalies" or "pterygium colli-intellectual disability-digital anomalies syndrome" or "pura-related severe neonatal hypotonia-seizures-encephalopathy syndrome" or "purine nucleoside phosphorylase deficiency" or "pycr2-related microcephaly-progressive leukoencephalopathy" or "pyridoxine-dependent epilepsy" or "pyruvate carboxylase deficiency" or "pyruvate dehydrogenase e1-alpha deficiency" or "pyruvate dehydrogenase e2 deficiency" or "pyruvate dehydrogenase e3-binding protein deficiency" or "pyruvate dehydrogenase phosphatase deficiency" or "rabson-mendenhall syndrome" or "radio-tartaglia syndrome" or "radioulnar synostosis with amegakaryocytic thrombocytopenia 2" or "radioulnar synostosis-microcephaly-scoliosis syndrome" or "rafiq syndrome" or "rahman syndrome" or "ramon syndrome" or "ramos-arroyo syndrome" or "rapid-onset childhood obesity-hypothalamic dysfunction-hypoventilation-autonomic dysregulation syndrome" or "rare non-syndromic intellectual disability" or "rars-related autosomal recessive hypomyelinating leukodystrophy" or "raynaud-claes syndrome" or "recessive intellectual disability-motor dysfunction-multiple joint contractures syndrome" or "recombinant 8 syndrome" or "recombinant chromosome 8 syndrome" or "recurrent metabolic encephalomyopathic crises-rhabdomyolysis-cardiac arrhythmia-intellectual disability syndrome" or "refsum disease" or "renal and mullerian duct hypoplasia" or "renal tubular acidosis, proximal, with ocular abnormalities and mental retardation" or "renpenning syndrome" or "rere-related neurodevelopmental syndrome" or "retinal dystrophy" or "retinitis pigmentosa" or "retinopathy, pigmentary, and mental retardation" or "rett syndrome" or "rhizomelic chondrodysplasia punctata" or "rhombencephalosynapsis" or "riboflavin transporter deficiency" or "richards-rundle syndrome" or "richieri costa-da silva syndrome" or "richieri-costa/guion-almeida syndrome" or "ring chromosome 10 syndrome" or "ring chromosome 13 syndrome" or "ring chromosome 14 syndrome" or "ring chromosome 21 syndrome" or "ring chromosome 8 syndrome" or "ritscher-schinzel syndrome 3" or "ritscher-schinzel syndrome 4" or "rnf13-related severe early-onset epileptic encephalopathy" or "roberts syndrome" or "robin sequence, distinctive facial appearance, and brachydactyly" or "rodrigues blindness" or "roifman syndrome" or "rolandic epilepsy" or "rothmund-thomson syndrome" or "rubinstein-taybi syndrome" or "ruvalcaba syndrome" or "sabinas brittle hair syndrome" or "saccharopinuria" or "saethre-chotzen syndrome" or "salla disease" or "sanjad-sakati syndrome" or "sarcosinemia" or "satb2" or "scarf syndrome" or "schaaf-yang syndrome" or "schilbach-rott syndrome" or "schimke x-linked mental retardation syndrome" or "schimmelpenning-feuerstein-mims syndrome" or "schindler disease, type i" or "schinzel-giedion midface-retraction syndrome" or "schizencephaly" or "scholte syndrome" or "schuurs-hoeijmakers syndrome" or "schwartz-jampel syndrome" or "seckel syndrome" or "seizures, sensorineural deafness, ataxia, mental retardation, and electrolyte imbalance" or "seizures-intellectual disability due to hydroxylysinuria syndrome" or "seizures-scoliosis-macrocephaly syndrome" or "semilobar holoprosencephaly" or "senior-loken syndrome 7" or "septo-optic dysplasia spectrum" or "septopreoptic holoprosencephaly" or "severe achondroplasia-developmental delay-acanthosis nigricans syndrome" or "severe growth deficiency-strabismus-extensive dermal melanocytosis-intellectual disability syndrome" or "severe intellectual disability and progressive spastic paraplegia" or "severe intellectual disability-corpus callosum agenesis-facial dysmorphism-cerebellar ataxia syndrome" or "severe intellectual disability-epilepsy-anal anomalies-distal phalangeal hypoplasia" or "severe intellectual disability-hypotonia-strabismus-coarse face-planovalgus syndrome" or "severe intellectual disability-poor language-strabismus-grimacing face-long fingers syndrome" or "severe intellectual disability-progressive postnatal microcephaly-midline stereotypic hand movements syndrome" or "severe intellectual disability-progressive spastic diplegia syndrome" or "severe intellectual disability-short stature-behavioral abnormalities-facial dysmorphism syndrome" or "severe oculo-renal-cerebellar syndrome" or "severe phosphoribosylpyrophosphate synthetase superactivity" or "shaheen syndrome" or "shashi-pena syndrome" or "short stature-brachydactyly-obesity-global developmental delay syndrome" or "short stature-craniofacial anomalies-genital hypoplasia syndrome" or "short stature-webbed neck-heart disease syndrome" or "short ulna-dysmorphism-hypotonia-intellectual disability syndrome" or "short-rib thoracic dysplasia 10" or "shprintzen-goldberg craniosynostosis syndrome" or "shprintzen-goldberg syndrome" or "shwachman-diamond syndrome" or "sialidosis type 1" or "sialuria" or "siderius x-linked mental retardation syndrome" or "sifrim-hitz-weiss syndrome" or "silver-russell syndrome" or "simha syndrome" or "simpson-golabi-behmel syndrome" or "sin3a-related intellectual disability syndrome due to a point mutation" or "sjogren-larsson syndrome" or "sj gren-larsson syndrome" or "skeletal defects, genital hypoplasia, and mental retardation" or "skeletal dysplasia-epilepsy-short stature syndrome" or "skeletal dysplasia-t-cell immunodeficiency-developmental delay syndrome" or "skin creases, congenital symmetric circumferential, 1" or "skin creases, congenital symmetric circumferential, 2" or "skraban-deardorff syndrome" or "slc35a2-cdg" or "slc39a8-cdg" or "smith-kingsmore syndrome" or "smith-lemli-opitz syndrome" or "smith-magenis syndrome" or "snijders blok-fisher syndrome" or "solitary median maxillary central incisor" or "sonoda syndrome" or "sotos syndrome" or "spastic ataxia, charlevoix-saguenay type" or "spastic diplegia, infantile type" or "spastic paraplegia 11, autosomal recessive" or "spastic paraplegia 14, autosomal recessive" or "spastic paraplegia 15, autosomal recessive" or "spastic paraplegia 16, x-linked" or "spastic paraplegia 18, autosomal recessive" or "spastic paraplegia 2, x-linked" or "spastic paraplegia 20, autosomal recessive" or "spastic paraplegia 26, autosomal recessive" or "spastic paraplegia 3, autosomal dominant" or "spastic paraplegia 32, autosomal recessive" or "spastic paraplegia 35, autosomal recessive" or "spastic paraplegia 4, autosomal dominant" or "spastic paraplegia 45, autosomal recessive" or "spastic paraplegia 46, autosomal recessive" or "spastic paraplegia 47, autosomal recessive" or "spastic paraplegia 48, autosomal recessive" or "spastic paraplegia 50, autosomal recessive" or "spastic paraplegia 51, autosomal recessive" or "spastic paraplegia 52, autosomal recessive" or "spastic paraplegia 54, autosomal recessive" or "spastic paraplegia 55, autosomal recessive" or "spastic paraplegia 64, autosomal recessive" or "spastic paraplegia 81, autosomal recessive" or "spastic paraplegia 82, autosomal recessive" or "spastic paraplegia 9b, autosomal recessive" or "spastic paraplegia and psychomotor retardation" or "spastic paraplegia type 2" or "spastic paraplegia, ataxia, and mental retardation" or "spastic paraplegia, epilepsy, and mental retardation" or "spastic paraplegia, intellectual disability, nystagmus, and obesity" or "spastic paraplegia, sensorineural deafness, mental retardation, and" or "spastic paraplegia-glaucoma-intellectual disability syndrome" or "spastic paraplegia-intellectual disability-nystagmus-obesity syndrome" or "spastic paraplegia-nephritis-deafness syndrome" or "spastic paraplegia-precocious puberty syndrome" or "spastic paraplegia-severe developmental delay-epilepsy syndrome" or "spastic paresis, glaucoma, and mental retardation" or "spastic quadriplegia, retinitis pigmentosa, and mental retardation" or "spastic tetraplegia, thin corpus callosum, and progressive microcephaly" or "spastic tetraplegia-retinitis pigmentosa-intellectual disability syndrome" or "spastic tetraplegia-thin corpus callosum-progressive postnatal microcephaly syndrome" or "spectrin-associated autosomal recessive cerebellar ataxia" or "spinal muscular atrophy with mental retardation" or "spinal muscular atrophy with microcephaly and mental subnormality" or "spinal muscular atrophy-progressive myoclonic epilepsy syndrome" or "spinocerebellar ataxia 13" or "spinocerebellar ataxia 21" or "spinocerebellar ataxia 27" or "spinocerebellar ataxia 29" or "spinocerebellar ataxia 42, early-onset, severe, with neurodevelopmental deficits" or "spinocerebellar ataxia 47" or "spinocerebellar ataxia type 13" or "spinocerebellar ataxia type 21" or "spinocerebellar ataxia type 27" or "spinocerebellar ataxia type 35" or "spinocerebellar ataxia, autosomal recessive 10" or "spinocerebellar ataxia, autosomal recessive 12" or "spinocerebellar ataxia, autosomal recessive 13" or "spinocerebellar ataxia, autosomal recessive 15" or "spinocerebellar ataxia, autosomal recessive 17" or "spinocerebellar ataxia, autosomal recessive 18" or "spinocerebellar ataxia, autosomal recessive 2" or "spinocerebellar ataxia, autosomal recessive 21" or "spinocerebellar ataxia, autosomal recessive 22" or "spinocerebellar ataxia, autosomal recessive 23" or "spinocerebellar ataxia, autosomal recessive 28" or "spinocerebellar ataxia, autosomal recessive 29" or "spinocerebellar ataxia, autosomal recessive 30" or "spinocerebellar ataxia, autosomal recessive 4" or "spinocerebellar degeneration and corneal dystrophy" or "spinocerebellar degeneration-corneal dystrophy syndrome" or "split-hand/foot malformation 1" or "split-hand/foot malformation 3" or "sponastrime dysplasia" or "spondylocostal dysostosis-hypospadias-intellectual disability syndrome" or "spondyloenchondrodysplasia" or "spondyloepimetaphyseal dysplasia with joint laxity" or "spondyloepimetaphyseal dysplasia, faden-alkuraya type" or "spondyloepimetaphyseal dysplasia, genevieve type" or "spondyloepimetaphyseal dysplasia, sponastrime type" or "spondyloepimetaphyseal dysplasia, x-linked, with mental deterioration" or "spondyloepiphyseal dysplasia tarda with mental retardation" or "spondyloepiphyseal dysplasia tarda, kohn type" or "spondylometaphyseal dysplasia, x-linked" or "spondylo-ocular syndrome" or "sporadic fetal brain disruption sequence" or "srd5a3-cdg" or "ssr4-cdg" or "stag1-related intellectual disability-facial dysmorphism-gastroesophageal reflux syndrome" or "stankiewicz-isidor syndrome" or "stargardt macular degeneration, absent or hypoplastic corpus callosum, mental retardation, and dysmorphic features" or "steinert myotonic dystrophy" or "stevenson-carey syndrome" or "stickler syndrome type 1" or "stimmler syndrome" or "stocco dos santos x-linked mental retardation syndrome" or "stomatin-deficient cryohydrocytosis with neurologic defects" or "striatal degeneration, autosomal dominant 2" or "striatonigral degeneration, infantile" or "structural brain anomalies with impaired intellectual development and craniosynostosis" or "stt3a-cdg" or "stt3b-cdg" or "sturge-weber syndrome" or "subaortic stenosis-short stature syndrome" or "succinic semialdehyde dehydrogenase deficiency" or "sucrosuria, hiatus hernia and mental retardation" or "summitt syndrome" or "suprabulbar paresis, congenital" or "symptomatic form of fragile x syndrome in female carriers" or "synaptic congenital myasthenic syndromes" or "syndromic diarrhea" or "syndromic recessive x-linked ichthyosis" or "syngap1-related developmental and epileptic encephalopathy" or "takenouchi-kosaki syndrome" or "tall stature-intellectual disability-renal anomalies syndrome" or "tarp syndrome" or "tatton-brown-rahman syndrome" or "telecanthus" or "telo2-related intellectual disability-neurodevelopmental disorder" or "temple syndrome" or "temple-baraitser syndrome" or "temtamy preaxial brachydactyly syndrome" or "temtamy syndrome" or "tenorio syndrome" or "tetra-amelia with ectodermal dysplasia and lacrimal duct abnormalities" or "tetramelic deficiencies, ectodermal dysplasia, deformed ears, andother abnormalities" or "tetrasomy 12p" or "tetrasomy 18p" or "thanatophoric dysplasia" or "thauvin-robinet-faivre syndrome" or "thoc6-related developmental delay-microcephaly-facial dysmorphism syndrome" or "thoracic dysplasia-hydrocephalus syndrome" or "thrombocytopenia, paris-trousseau type" or "thrombocytopenia-absent radius syndrome" or "thumb deformity and alopecia" or "thumb deformity-alopecia-pigmentation anomaly syndrome" or "thumb stiffness-brachydactyly-intellectual disability syndrome" or "thumbs, stiff, with brachydactyly type a1 and developmental delay" or "thyroid ectopia" or "genetic defect in thyroid hormonogenesis" or "thyroid hypoplasia" or "thyrotropin-releasing hormone deficiency" or "timothy syndrome" or "tmem70-related mitochondrial encephalo-cardio-myopathy" or "tonne-kalscheuer syndrome" or "townes-brocks syndrome" or "transcobalamin ii deficiency" or "transketolase deficiency" or "trappc11-related limb-girdle muscular dystrophy r18" or "treacher collins-franceschetti syndrome" or "tremor-ataxia-central hypomyelination syndrome" or "trichorhinophalangeal syndrome type 2" or "trichorhinophalangeal syndrome, type ii" or "trichothiodystrophy" or "triglyceride deposit cardiomyovasculopathy" or "trigonocephaly with short stature and developmental delay" or "trigonocephaly-short stature-developmental delay syndrome" or "trisomy 10p" or "trisomy 12p" or "trisomy 13" or "trisomy 17p" or "trisomy 18p" or "trisomy 5p" or "trisomy 8p" or "trisomy 9p" or "tryptophanuria with dwarfism" or "t-substance anomaly" or "tuberous sclerosis complex" or "tuberous sclerosis-1" or "tuberous sclerosis-2" or "turnpenny-fry syndrome" or "tyrosine transaminase deficiency" or "tyrosinemia type 2" or "tyrosinemia, type iii" or "tyshchenko syndrome" or "ulna hypoplasia-intellectual disability syndrome" or "ulnar hypoplasia with mental retardation" or "unilateral focal polymicrogyria" or "unilateral hemispheric polymicrogyria" or "unilateral polymicrogyria" or "urban-rogers-meyer syndrome" or "urocanase deficiency" or "usher syndrome type 1" or "usmani-riazuddin syndrome" or "uveal coloboma-cleft lip and palate-intellectual disability" or "vacterl with hydrocephalus" or "van bogaert-hozay syndrome" or "van den bosch syndrome" or "van esch-o'driscoll syndrome" or "van maldergem syndrome 1" or "van maldergem syndrome 2" or "velocardiofacial syndrome" or "ventricular extrasystoles with syncopal episodes-perodactyly-robin sequence syndrome" or "vertebral, cardiac, renal, and limb defects syndrome 1" or "ververi-brady syndrome" or "vici syndrome" or "viss syndrome" or "vissers-bodmer syndrome" or "vitamin b12-responsive methylmalonic acidemia" or "vitamin b12-unresponsive methylmalonic acidemia" or "vitamin b12-unresponsive methylmalonic acidemia type mut-" or "vitamin b12-unresponsive methylmalonic acidemia type mut0" or "vitamin k antagonist embryofetopathy" or "vitiligo, progressive, with mental retardation and urethral duplication" or "vps11-related autosomal recessive hypomyelinating leukodystrophy" or "waardenburg syndrome type 3" or "waardenburg syndrome, type 2e" or "waardenburg syndrome, type 3" or "wac-related facial dysmorphism-developmental delay-behavioral abnormalities syndrome" or "wagr 11p13 deletion syndrome" or "wagr syndrome" or "waisman syndrome" or "walker-warburg syndrome" or "warburg micro syndrome" or "wars2-related combined oxidative phosphorylation defect" or "warsaw breakage syndrome" or "weaver syndrome" or "weaver-williams syndrome" or "weill-marchesani syndrome" or "weismann-netter syndrome" or "weiss-kruszka syndrome" or "white matter hypoplasia-corpus callosum agenesis-intellectual disability syndrome" or "white-kernohan syndrome" or "white-sutton syndrome" or "wieacker-wolff syndrome" or "wiedemann-rautenstrauch syndrome" or "wiedemann-steiner syndrome" or "williams syndrome" or "williams-beuren syndrome" or "wilms tumor, aniridia, genitourinary anomalies, and mental retardation syndrome" or "wilson disease" or "wilson-turner syndrome" or "witteveen-kolk syndrome" or "wolcott-rallison syndrome" or "wolf-hirschhorn syndrome" or "wolfram syndrome" or "woodhouse-sakati syndrome" or "woods syndrome" or "wrinkly skin syndrome" or "wyburn-mason syndrome" or "x small rings" or "xeroderma pigmentosum" or "xeroderma pigmentosum-cockayne syndrome complex" or "xfe progeroid syndrome" or "xia-gibbs syndrome" or "x-linked acrogigantism" or "x-linked adrenoleukodystrophy" or "x-linked cerebral-cerebellar-coloboma syndrome" or "x-linked charcot-marie-tooth disease type 2" or "x-linked charcot-marie-tooth disease type 4" or "x-linked complicated corpus callosum dysgenesis" or "x-linked complicated spastic paraplegia type 1" or "x-linked creatine transporter deficiency" or "x-linked dominant chondrodysplasia, chassaing-lacombe type" or "x-linked female restricted facial dysmorphism-short stature-choanal atresia-intellectual disability" or "x-linked lissencephaly with abnormal genitalia" or "x-linked neurodegenerative syndrome, bertini type").ti,kf,ab. ("x-linked neurodegenerative syndrome, hamel type" or "x-linked non-syndromic intellectual disability" or "x-linked spastic paraplegia type 16" or "x-linked spasticity-intellectual disability-epilepsy syndrome" or "xp21 deletion syndrome" or "xp22.13p22.2 duplication syndrome" or "xq12-q13.3 duplication syndrome" or "xq21 deletion syndrome" or "xq21 microdeletion syndrome" or "xq25 duplication syndrome" or "xq25 microduplication syndrome" or "xq27.3q28 duplication syndrome" or "xq28 (mecp2) duplication" or "xy type gonadal dysgenesis-associated anomalies syndrome" or "xylt1-cdg" or "yuan-harel-lupski syndrome" or "zechi-ceide syndrome" or "zellweger-like syndrome without peroxisomal anomalies" or "zimmermann-laband syndrome" or "zttk syndrome" or "zunich neuroectodermal syndrome" or "14q12 microdeletion syndrome" or "22q11.2 microduplication syndrome" or "3-phosphoserine phosphatase deficiency" or "carpenter-waziri syndrome" or "chudley-lowry-hoar syndrome" or "clapo syndrome" or "classic congenital adrenal hyperplasia due to 21-hydroxylase deficiency" or "coenzyme q10 deficiency" or "congenital cerebellar ataxia due to rnu12 mutation" or "goldenhar syndrome" or "hair defect-photosensitivity-intellectual disability syndrome" or "holmes-gang syndrome" or "hutchinson-gilford progeria syndrome" or "hyperinsulinism due to hnf1a deficiency" or "hypothyroidism due to deficient transcription factors involved in pituitary development or function" or "intellectual disability-feeding difficulties-developmental delay-microcephaly syndrome" or "intellectual disability-microcephaly-strabismus-behavioral abnormalities syndrome" or "intellectual disability-sparse hair-brachydactyly syndrome" or "isolated spina bifida" or "juberg-marsidi syndrome" or "juvenile myoclonic epilepsy" or "kid syndrome" or "leukoencephalopathy-spondylometaphyseal dysplasia syndrome" or "lissencephaly type 1 due to doublecortin gene mutation" or "methylmalonic acidemia without homocystinuria" or "mgat2-cdg" or "microcephalic primordial dwarfism due to rttn deficiency" or "microcephalic primordial dwarfism, alazami type" or "mucopolysaccharidosis type 2" or "nakajo-nishimura syndrome" or "nephrogenic diabetes insipidus-intracranial calcification syndrome" or "neuroferritinopathy" or "neuronal ceroid lipofuscinosis" or "opitz g/bbb syndrome" or "osteopenia-myopia-hearing loss-intellectual disability-facial dysmorphism syndrome" or "osteopetrosis and related disorders" or "otomandibular syndrome" or "peripheral hypothyroidism" or "pitt-hopkins-like syndrome" or "renier-gabreels-jasper syndrome" or "salt-and-pepper syndrome" or "severe canavan disease" or "severe feeding difficulties-failure to thrive-microcephaly due to asxl3 deficiency syndrome" or "severe intellectual disability-epilepsy-cataract syndrome due to fatty acyl-coa reductase 1 deficiency" or "smith-fineman-myers syndrome" or "spinocerebellar ataxia type 42" or "spinocerebellar ataxia with axonal neuropathy type 1" or "tall stature-intellectual disability-facial dysmorphism syndrome" or "tay-sachs disease" or "thyrocerebrorenal syndrome" or "transient neonatal diabetes mellitus" or "trisomy xq28" or "undetermined early-onset epileptic encephalopathy" or "unverricht-lundborg disease" or "x-linked charcot-marie-tooth disease type 3" or "xp21 microdeletion syndrome" or "zlotogora-ogur syndrome" or "x-linked intellectual disability").ti,kf,ab. “Sabinas Brittle Hair Syndrome”[tiab] OR “Saccharopinuria”[tiab] OR “Saethre-chotzen Syndrome”[tiab] OR “Salla Disease”[tiab] OR “Sanjad-sakati Syndrome”[tiab] OR “Sarcosinemia”[tiab] OR “Satb2-associated Syndrome Due To A Chromosomal Rearrangement”[tiab] OR “Satb2-associated Syndrome Due To A Pathogenic Variant”[tiab] OR “Scarf Syndrome”[tiab] OR “Schaaf-yang Syndrome”[tiab] OR “Schilbach-rott Syndrome”[tiab] OR “Schimke X-linked Mental Retardation Syndrome”[tiab] OR “Schimmelpenning-feuerstein-mims Syndrome”[tiab] OR “Schindler Disease, Type I”[tiab] OR “Schinzel-giedion Midface-retraction Syndrome”[tiab] OR “Schizencephaly”[tiab] OR “Scholte Syndrome”[tiab] OR “Schuurs-hoeijmakers Syndrome”[tiab] OR “Schwartz-jampel Syndrome”[tiab] OR “Schwartz-jampel Syndrome, Type 1”[tiab] OR “Seckel Syndrome”[tiab] OR “Seckel Syndrome 1”[tiab] OR “Seckel Syndrome 5”[tiab] OR “Seckel Syndrome 6”[tiab] OR “Seckel Syndrome 7”[tiab] OR “Seckel Syndrome 8”[tiab] OR “Seizures, Sensorineural Deafness, Ataxia, Mental Retardation, And Electrolyte Imbalance”[tiab] OR “Seizures-intellectual Disability Due To Hydroxylysinuria Syndrome”[tiab] OR “Seizures-scoliosis-macrocephaly Syndrome”[tiab] OR “Semilobar Holoprosencephaly”[tiab] OR “Senior-loken Syndrome 7”[tiab] OR “Septo-optic Dysplasia Spectrum”[tiab] OR “Septopreoptic Holoprosencephaly”[tiab] OR “Severe Achondroplasia-developmental Delay-acanthosis Nigricans Syndrome”[tiab] OR “Severe Growth Deficiency-strabismus-extensive Dermal Melanocytosis-intellectual Disability Syndrome”[tiab] OR “Severe Intellectual Disability And Progressive Spastic Paraplegia”[tiab] OR “Severe Intellectual Disability-corpus Callosum Agenesis-facial Dysmorphism-cerebellar Ataxia Syndrome”[tiab] OR “Severe Intellectual Disability-epilepsy-anal Anomalies-distal Phalangeal Hypoplasia”[tiab] OR “Severe Intellectual Disability-hypotonia-strabismus-coarse Face-planovalgus Syndrome”[tiab] OR “Severe Intellectual Disability-poor Language-strabismus-grimacing Face-long Fingers Syndrome”[tiab] OR “Severe Intellectual Disability-progressive Postnatal Microcephaly-midline Stereotypic Hand Movements Syndrome”[tiab] OR “Severe Intellectual Disability-progressive Spastic Diplegia Syndrome”[tiab] OR “Severe Intellectual Disability-short Stature-behavioral Abnormalities-facial Dysmorphism Syndrome”[tiab] OR “Severe Neonatal Hypotonia-seizures-encephalopathy Syndrome Due To 5q31.3 Microdeletion”[tiab] OR “Severe Neurodevelopmental Disorder With Feeding Difficulties-stereotypic Hand Movement-bilateral Cataract”[tiab] OR “Severe Oculo-renal-cerebellar Syndrome”[tiab] OR “Severe Phosphoribosylpyrophosphate Synthetase Superactivity”[tiab] OR “Severe X-linked Intellectual Disability, Gustavson Type”[tiab] OR “Shaheen Syndrome”[tiab] OR “Shashi-pena Syndrome”[tiab] OR “Short Stature, Brachydactyly, Intellectual Developmental Disability, And Seizures”[tiab] OR “Short Stature, Developmental Delay, And Congenital Heart Defects”[tiab] OR “Short Stature, Facial Dysmorphism, And Skeletal Anomalies With Or Without Cardiac Anomalies”[tiab] OR “Short Stature, Hearing Loss, Retinitis Pigmentosa, And Distinctive Facies”[tiab] OR “Short Stature, Rhizomelic, With Microcephaly, Micrognathia, And Developmental Delay”[tiab] OR “Short Stature-brachydactyly-obesity-global Developmental Delay Syndrome”[tiab] OR “Short Stature-craniofacial Anomalies-genital Hypoplasia Syndrome”[tiab] OR “Short Stature-delayed Bone Age Due To Thyroid Hormone Metabolism Deficiency”[tiab] OR “Short Stature-webbed Neck-heart Disease Syndrome”[tiab] OR “Short Ulna-dysmorphism-hypotonia-intellectual Disability Syndrome”[tiab] OR “Short-rib Thoracic Dysplasia 10 With Or Without Polydactyly”[tiab] OR “Shprintzen-goldberg Craniosynostosis Syndrome”[tiab] OR “Shprintzen-goldberg Syndrome”[tiab] OR “Shwachman-diamond Syndrome”[tiab] OR “Shwachman-diamond Syndrome 1”[tiab] OR “Sialidosis Type 1”[tiab] OR “Sialuria”[tiab] OR “Siderius X-linked Mental Retardation Syndrome”[tiab] OR “Sifrim-hitz-weiss Syndrome”[tiab] OR “Silver-russell Syndrome”[tiab] OR “Silver-russell Syndrome Due To 7p11.2p13 Microduplication”[tiab] OR “Silver-russell Syndrome Due To An Imprinting Defect Of 11p15”[tiab] OR “Sim1-related Prader-willi-like Syndrome”[tiab] OR “Simha Syndrome”[tiab] OR “Simpson-golabi-behmel Syndrome”[tiab] OR “Simpson-golabi-behmel Syndrome, Type 2”[tiab] OR “Sin3a-related Intellectual Disability Syndrome Due To A Point Mutation”[tiab] OR “Sjogren-larsson Syndrome”[tiab] OR “Sjögren-larsson Syndrome”[tiab] OR “Skeletal Defects, Genital Hypoplasia, And Mental Retardation”[tiab] OR “Skeletal Dysplasia-epilepsy-short Stature Syndrome”[tiab] OR “Skeletal Dysplasia-t-cell Immunodeficiency-developmental Delay Syndrome”[tiab] OR “Skin Creases, Congenital Symmetric Circumferential, 1”[tiab] OR “Skin Creases, Congenital Symmetric Circumferential, 2”[tiab] OR “Skraban-deardorff Syndrome”[tiab] OR “Slc35a2-cdg”[tiab] OR “Slc39a8-cdg”[tiab] OR “Smith-kingsmore Syndrome”[tiab] OR “Smith-lemli-opitz Syndrome”[tiab] OR “Smith-magenis Syndrome”[tiab] OR “Snijders Blok-fisher Syndrome”[tiab] OR “Solitary Median Maxillary Central Incisor”[tiab] OR “Sonoda Syndrome”[tiab] OR “Sotos Syndrome”[tiab] OR “Spastic Ataxia 9, Autosomal Recessive”[tiab] OR “Spastic Ataxia, Charlevoix-saguenay Type”[tiab] OR “Spastic Diplegia, Infantile Type”[tiab] OR “Spastic Paraplegia 11, Autosomal Recessive”[tiab] OR “Spastic Paraplegia 14, Autosomal Recessive”[tiab] OR “Spastic Paraplegia 15, Autosomal Recessive”[tiab] OR “Spastic Paraplegia 16, X-linked”[tiab] OR “Spastic Paraplegia 18, Autosomal Recessive”[tiab] OR “Spastic Paraplegia 2, X-linked”[tiab] OR “Spastic Paraplegia 20, Autosomal Recessive”[tiab] OR “Spastic Paraplegia 26, Autosomal Recessive”[tiab] OR “Spastic Paraplegia 3, Autosomal Dominant”[tiab] OR “Spastic Paraplegia 32, Autosomal Recessive”[tiab] OR “Spastic Paraplegia 35, Autosomal Recessive”[tiab] OR “Spastic Paraplegia 4, Autosomal Dominant”[tiab] OR “Spastic Paraplegia 45, Autosomal Recessive”[tiab] OR “Spastic Paraplegia 46, Autosomal Recessive”[tiab] OR “Spastic Paraplegia 47, Autosomal Recessive”[tiab] OR “Spastic Paraplegia 48, Autosomal Recessive”[tiab] OR “Spastic Paraplegia 50, Autosomal Recessive”[tiab] OR “Spastic Paraplegia 51, Autosomal Recessive”[tiab] OR “Spastic Paraplegia 52, Autosomal Recessive”[tiab] OR “Spastic Paraplegia 54, Autosomal Recessive”[tiab] OR “Spastic Paraplegia 55, Autosomal Recessive”[tiab] OR “Spastic Paraplegia 56, Autosomal Recessive”[tiab] OR “Spastic Paraplegia 61, Autosomal Recessive”[tiab] OR “Spastic Paraplegia 63, Autosomal Recessive”[tiab] OR “Spastic Paraplegia 64, Autosomal Recessive”[tiab] OR “Spastic Paraplegia 81, Autosomal Recessive”[tiab] OR “Spastic Paraplegia 82, Autosomal Recessive”[tiab] OR “Spastic Paraplegia 9b, Autosomal Recessive”[tiab] OR “Spastic Paraplegia And Psychomotor Retardation With Or Without Seizures”[tiab] OR “Spastic Paraplegia Type 2”[tiab] OR “Spastic Paraplegia, Ataxia, And Mental Retardation”[tiab] OR “Spastic Paraplegia, Epilepsy, And Mental Retardation”[tiab] OR “Spastic Paraplegia, Intellectual Disability, Nystagmus, And Obesity”[tiab] OR “Spastic Paraplegia, Sensorineural Deafness, Mental Retardation, And”[tiab] OR “Spastic Paraplegia-glaucoma-intellectual Disability Syndrome”[tiab] OR “Spastic Paraplegia-intellectual Disability-nystagmus-obesity Syndrome”[tiab] OR “Spastic Paraplegia-nephritis-deafness Syndrome”[tiab] OR “Spastic Paraplegia-precocious Puberty Syndrome”[tiab] OR “Spastic Paraplegia-severe Developmental Delay-epilepsy Syndrome”[tiab] OR “Spastic Paresis, Glaucoma, And Mental Retardation”[tiab] OR “Spastic Quadriplegia, Retinitis Pigmentosa, And Mental Retardation”[tiab] OR “Spastic Tetraplegia, Thin Corpus Callosum, And Progressive Microcephaly”[tiab] OR “Spastic Tetraplegia-retinitis Pigmentosa-intellectual Disability Syndrome”[tiab] OR “Spastic Tetraplegia-thin Corpus Callosum-progressive Postnatal Microcephaly Syndrome”[tiab] OR “Spectrin-associated Autosomal Recessive Cerebellar Ataxia”[tiab] OR “Spinal Muscular Atrophy With Mental Retardation”[tiab] OR “Spinal Muscular Atrophy With Microcephaly And Mental Subnormality”[tiab] OR “Spinal Muscular Atrophy-progressive Myoclonic Epilepsy Syndrome”[tiab] OR “Spinocerebellar Ataxia 13”[tiab] OR “Spinocerebellar Ataxia 21”[tiab] OR “Spinocerebellar Ataxia 27”[tiab] OR “Spinocerebellar Ataxia 29, Congenital Nonprogressive”[tiab] OR “Spinocerebellar Ataxia 42, Early-onset, Severe, With Neurodevelopmental Deficits”[tiab] OR “Spinocerebellar Ataxia 47”[tiab] OR “Spinocerebellar Ataxia Type 13”[tiab] OR “Spinocerebellar Ataxia Type 21”[tiab] OR “Spinocerebellar Ataxia Type 27”[tiab] OR “Spinocerebellar Ataxia Type 35”[tiab] OR “Spinocerebellar Ataxia, Autosomal Recessive 10”[tiab] OR “Spinocerebellar Ataxia, Autosomal Recessive 12”[tiab] OR “Spinocerebellar Ataxia, Autosomal Recessive 13”[tiab] OR “Spinocerebellar Ataxia, Autosomal Recessive 15”[tiab] OR “Spinocerebellar Ataxia, Autosomal Recessive 17”[tiab] OR “Spinocerebellar Ataxia, Autosomal Recessive 18”[tiab] OR “Spinocerebellar Ataxia, Autosomal Recessive 2”[tiab] OR “Spinocerebellar Ataxia, Autosomal Recessive 21”[tiab] OR “Spinocerebellar Ataxia, Autosomal Recessive 22”[tiab] OR “Spinocerebellar Ataxia, Autosomal Recessive 23”[tiab] OR “Spinocerebellar Ataxia, Autosomal Recessive 28”[tiab] OR “Spinocerebellar Ataxia, Autosomal Recessive 29”[tiab] OR “Spinocerebellar Ataxia, Autosomal Recessive 30”[tiab] OR “Spinocerebellar Ataxia, Autosomal Recessive 4”[tiab] OR “Spinocerebellar Degeneration And Corneal Dystrophy”[tiab] OR “Spinocerebellar Degeneration-corneal Dystrophy Syndrome”[tiab] OR “Split-hand/foot Malformation 1”[tiab] OR “Split-hand/foot Malformation 3”[tiab] OR “Sponastrime Dysplasia”[tiab] OR “Spondylo-ocular Syndrome”[tiab] OR “Spondyloenchondrodysplasia”[tiab] OR “Spondyloenchondrodysplasia With Immune Dysregulation”[tiab] OR “Spondyloepimetaphyseal Dysplasia With Joint Laxity”[tiab] OR “Spondyloepimetaphyseal Dysplasia, Faden-alkuraya Type”[tiab] OR “Spondyloepimetaphyseal Dysplasia, Genevieve Type”[tiab] OR “Spondyloepimetaphyseal Dysplasia, Sponastrime Type”[tiab] OR “Spondyloepimetaphyseal Dysplasia, X-linked, With Mental Deterioration”[tiab] OR “Spondyloepiphyseal Dysplasia Tarda With Mental Retardation”[tiab] OR “Spondyloepiphyseal Dysplasia Tarda, Kohn Type”[tiab] OR “Spondylometaphyseal Dysplasia, X-linked”[tiab] OR “Sporadic Fetal Brain Disruption Sequence”[tiab] OR “Srd5a3-cdg”[tiab] OR “Ssr4-cdg”[tiab] OR “Stag1-related Intellectual Disability-facial Dysmorphism-gastroesophageal Reflux Syndrome”[tiab] OR “Stankiewicz-isidor Syndrome”[tiab] OR “Stargardt Macular Degeneration, Absent Or Hypoplastic Corpus Callosum,mental Retardation, And Dysmorphic Features”[tiab] OR “Steinert Myotonic Dystrophy”[tiab] OR “Stevenson-carey Syndrome”[tiab] OR “Stickler Syndrome Type 1”[tiab] OR “Stimmler Syndrome”[tiab] OR “Stocco Dos Santos X-linked Mental Retardation Syndrome”[tiab] OR “Stomatin-deficient Cryohydrocytosis With Neurologic Defects”[tiab] OR “Striatal Degeneration, Autosomal Dominant 2”[tiab] OR “Striatonigral Degeneration, Infantile”[tiab] OR “Striatonigral Degeneration, Infantile, Mitochondrial”[tiab] OR “Structural Brain Anomalies With Impaired Intellectual Development And Craniosynostosis”[tiab] OR “Stt3a-cdg”[tiab] OR “Stt3b-cdg”[tiab] OR “Sturge-weber Syndrome”[tiab] OR “Subaortic Stenosis--short Stature Syndrome”[tiab] OR “Subaortic Stenosis-short Stature Syndrome”[tiab] OR “Succinic Semialdehyde Dehydrogenase Deficiency”[tiab] OR “Sucrosuria, Hiatus Hernia And Mental Retardation”[tiab] OR “Sulfite Oxidase Deficiency”[tiab] OR “Summitt Syndrome”[tiab] OR “Suprabulbar Paresis, Congenital”[tiab] OR “Symptomatic Form Of Fragile X Syndrome In Female Carriers”[tiab] OR “Synaptic Congenital Myasthenic Syndromes”[tiab] OR “Syndromic Diarrhea”[tiab] OR “Syndromic Recessive X-linked Ichthyosis”[tiab] OR “Syndromic X-linked Intellectual Disability 7”[tiab] OR “Syngap1-related Developmental And Epileptic Encephalopathy”[tiab] OR “T-substance Anomaly”[tiab] OR “Takenouchi-kosaki Syndrome”[tiab] OR “Tall Stature-intellectual Disability-renal Anomalies Syndrome”[tiab] OR “Tarp Syndrome”[tiab] OR “Tatton-brown-rahman Syndrome”[tiab] OR “Telecanthus”[tiab] OR “Telo2-related Intellectual Disability-neurodevelopmental Disorder”[tiab] OR “Temple Syndrome”[tiab] OR “Temple Syndrome Due To Maternal Uniparental Disomy Of Chromosome 14”[tiab] OR “Temple Syndrome Due To Paternal 14q32.2 Hypomethylation”[tiab] OR “Temple Syndrome Due To Paternal 14q32.2 Microdeletion”[tiab] OR “Temple-baraitser Syndrome”[tiab] OR “Temtamy Preaxial Brachydactyly Syndrome”[tiab] OR “Temtamy Syndrome”[tiab] OR “Tenorio Syndrome”[tiab] OR “Tetra-amelia With Ectodermal Dysplasia And Lacrimal Duct Abnormalities”[tiab] OR “Tetramelic Deficiencies, Ectodermal Dysplasia, Deformed Ears, Andother Abnormalities”[tiab] OR “Tetrasomy 12p”[tiab] OR “Tetrasomy 18p”[tiab] OR “Thanatophoric Dysplasia”[tiab] OR “Thanatophoric Dysplasia Type 1”[tiab] OR “Thanatophoric Dysplasia, Type I”[tiab] OR “Thauvin-robinet-faivre Syndrome”[tiab] OR “Thoc6-related Developmental Delay-microcephaly-facial Dysmorphism Syndrome”[tiab] OR “Thoracic Dysplasia-hydrocephalus Syndrome”[tiab] OR “Thrombocytopenia, Paris-trousseau Type”[tiab] OR “Thrombocytopenia-absent Radius Syndrome”[tiab] OR “Thumb Deformity And Alopecia”[tiab] OR “Thumb Deformity-alopecia-pigmentation Anomaly Syndrome”[tiab] OR “Thumb Stiffness-brachydactyly-intellectual Disability Syndrome”[tiab] OR “Thumbs, Stiff, With Brachydactyly Type A1 And Developmental Delay”[tiab] OR “Thyroid Ectopia”[tiab] OR “Thyroid Hormonogenesis, Genetic Defect In, 1”[tiab] OR “Thyroid Hormonogenesis, Genetic Defect In, 3”[tiab] OR “Thyroid Hormonogenesis, Genetic Defect In, 4”[tiab] OR “Thyroid Hormonogenesis, Genetic Defect In, 5”[tiab] OR “Thyroid Hypoplasia”[tiab] OR “Thyrotropin-releasing Hormone Deficiency”[tiab] OR “Timothy Syndrome”[tiab] OR “Tmem70-related Mitochondrial Encephalo-cardio-myopathy”[tiab] OR “Tonne-kalscheuer Syndrome”[tiab] OR “Townes-brocks Syndrome”[tiab] OR “Townes-brocks Syndrome 1”[tiab] OR “Transcobalamin Ii Deficiency”[tiab] OR “Transketolase Deficiency”[tiab] OR “Trappc11-related Limb-girdle Muscular Dystrophy R18”[tiab] OR “Treacher Collins Syndrome 1”[tiab] OR “Tremor-ataxia-central Hypomyelination Syndrome”[tiab] OR “Trichorhinophalangeal Syndrome Type 2”[tiab] OR “Trichorhinophalangeal Syndrome, Type Ii”[tiab] OR “Trichothiodystrophy 1, Photosensitive”[tiab] OR “Trichothiodystrophy 2, Photosensitive”[tiab] OR “Trichothiodystrophy 3, Photosensitive”[tiab] OR “Trichothiodystrophy 4, Nonphotosensitive”[tiab] OR “Trichothiodystrophy 5, Nonphotosensitive”[tiab] OR “Trichothiodystrophy 6, Nonphotosensitive”[tiab] OR “Trichothiodystrophy 9, Nonphotosensitive”[tiab] OR “Triglyceride Deposit Cardiomyovasculopathy”[tiab] OR “Trigonocephaly With Short Stature And Developmental Delay”[tiab] OR “Trigonocephaly-short Stature-developmental Delay Syndrome”[tiab] OR “Trisomy 10p”[tiab] OR “Trisomy 12p”[tiab] OR “Trisomy 13”[tiab] OR “Trisomy 17p”[tiab] OR “Trisomy 18p”[tiab] OR “Trisomy 5p”[tiab] OR “Trisomy 8p”[tiab] OR “Trisomy 9p”[tiab] OR “Tryptophanuria With Dwarfism”[tiab] OR “Tuberous Sclerosis Complex”[tiab] OR “Tuberous Sclerosis-1”[tiab] OR “Tuberous Sclerosis-2”[tiab] OR “Turnpenny-fry Syndrome”[tiab] OR “Tyrosine Transaminase Deficiency”[tiab] OR “Tyrosinemia Type 2”[tiab] OR “Tyrosinemia, Type Iii”[tiab] OR “Tyshchenko Syndrome”[tiab] OR “Ulna Hypoplasia-intellectual Disability Syndrome”[tiab] OR “Ulnar Hypoplasia With Mental Retardation”[tiab] OR “Unilateral Focal Polymicrogyria”[tiab] OR “Unilateral Hemispheric Polymicrogyria”[tiab] OR “Unilateral Polymicrogyria”[tiab] OR “Urban-rogers-meyer Syndrome”[tiab] OR “Urocanase Deficiency”[tiab] OR “Usher Syndrome Type 1”[tiab] OR “Usmani-riazuddin Syndrome, Autosomal Dominant”[tiab] OR “Usmani-riazuddin Syndrome, Autosomal Recessive”[tiab] OR “Uveal Coloboma-cleft Lip And Palate-intellectual Disability”[tiab] OR “Vacterl With Hydrocephalus”[tiab] OR “Van Bogaert-hozay Syndrome”[tiab] OR “Van Den Bosch Syndrome”[tiab] OR “Van Esch-o'driscoll Syndrome”[tiab] OR “Van Maldergem Syndrome 1”[tiab] OR “Van Maldergem Syndrome 2”[tiab] OR “Velocardiofacial Syndrome”[tiab] OR “Ventricular Extrasystoles With Syncopal Episodes-perodactyly-robin Sequence Syndrome”[tiab] OR “Vertebral, Cardiac, Renal, And Limb Defects Syndrome 1”[tiab] OR “Ververi-brady Syndrome”[tiab] OR “Vici Syndrome”[tiab] OR “Viss Syndrome”[tiab] OR “Vissers-bodmer Syndrome”[tiab] OR “Vitamin B12-responsive Methylmalonic Acidemia”[tiab] OR “Vitamin B12-unresponsive Methylmalonic Acidemia”[tiab] OR “Vitamin B12-unresponsive Methylmalonic Acidemia Type Mut-”[tiab] OR “Vitamin B12-unresponsive Methylmalonic Acidemia Type Mut0”[tiab] OR “Vitamin K Antagonist Embryofetopathy”[tiab] OR “Vitiligo, Progressive, With Mental Retardation And Urethral Duplication”[tiab] OR “Vps11-related Autosomal Recessive Hypomyelinating Leukodystrophy”[tiab] OR “Waardenburg Syndrome Type 3”[tiab] OR “Waardenburg Syndrome, Type 2e”[tiab] OR “Waardenburg Syndrome, Type 3”[tiab] OR “Wac-related Facial Dysmorphism-developmental Delay-behavioral Abnormalities Syndrome”[tiab] OR “Wagr 11p13 Deletion Syndrome”[tiab] OR “Wagr Syndrome”[tiab] OR “Waisman Syndrome”[tiab] OR “Walker-warburg Syndrome”[tiab] OR “Warburg Micro Syndrome 1”[tiab] OR “Warburg Micro Syndrome 2”[tiab] OR “Warburg Micro Syndrome 3”[tiab] OR “Warburg Micro Syndrome 4”[tiab] OR “Wars2-related Combined Oxidative Phosphorylation Defect”[tiab] OR “Warsaw Breakage Syndrome”[tiab] OR “Weaver Syndrome”[tiab] OR “Weaver-williams Syndrome”[tiab] OR “Weill-marchesani Syndrome”[tiab] OR “Weill-marchesani Syndrome 1”[tiab] OR “Weill-marchesani Syndrome 2, Dominant”[tiab] OR “Weismann-netter Syndrome”[tiab] OR “Weiss-kruszka Syndrome”[tiab] OR “White Matter Hypoplasia-corpus Callosum Agenesis-intellectual Disability Syndrome”[tiab] OR “White-kernohan Syndrome”[tiab] OR “White-sutton Syndrome”[tiab] OR “Wieacker-wolff Syndrome”[tiab] OR “Wiedemann-rautenstrauch Syndrome”[tiab] OR “Wiedemann-steiner Syndrome”[tiab] OR “Williams Syndrome”[tiab] OR “Williams-beuren Syndrome”[tiab] OR “Williams-beuren Syndrome (wbs)”[tiab] OR “Wilms Tumor, Aniridia, Genitourinary Anomalies, And Mental Retardationsyndrome”[tiab] OR “Wilson Disease”[tiab] OR “Wilson-turner Syndrome”[tiab] OR “Witteveen-kolk Syndrome”[tiab] OR “Wolcott-rallison Syndrome”[tiab] OR “Wolf-hirschhorn Syndrome”[tiab] OR “Wolfram Syndrome”[tiab] OR “Wolfram Syndrome 1”[tiab] OR “Wolfram Syndrome, Mitochondrial Form”[tiab] OR “Woodhouse-sakati Syndrome”[tiab] OR “Woods Syndrome”[tiab] OR “Wrinkly Skin Syndrome”[tiab] OR “Wyburn-mason Syndrome”[tiab] OR “X Small Rings”[tiab] OR “X-linked Acrogigantism”[tiab] OR “X-linked Adrenoleukodystrophy”[tiab] OR “X-linked Cerebral-cerebellar-coloboma Syndrome”[tiab] OR “X-linked Charcot-marie-tooth Disease Type 2”[tiab] OR “X-linked Charcot-marie-tooth Disease Type 4”[tiab] OR “X-linked Complicated Corpus Callosum Dysgenesis”[tiab] OR “X-linked Complicated Spastic Paraplegia Type 1”[tiab] OR “X-linked Creatine Transporter Deficiency”[tiab] OR “X-linked Dominant Chondrodysplasia, Chassaing-lacombe Type”[tiab] OR “X-linked Female Restricted Facial Dysmorphism-short Stature-choanal Atresia-intellectual Disability”[tiab] OR “X-linked Intellectual Disability Due To Gria3 Mutations”[tiab] OR “X-linked Intellectual Disability With Isolated Growth Hormone Deficiency”[tiab] OR “X-linked Intellectual Disability, Abidi Type”[tiab] OR “X-linked Intellectual Disability, Armfield Type”[tiab] OR “X-linked Intellectual Disability, Cabezas Type”[tiab] OR “X-linked Intellectual Disability, Cantagrel Type”[tiab] OR “X-linked Intellectual Disability, Cilliers Type”[tiab] OR “X-linked Intellectual Disability, Golabi-ito-hall Type”[tiab] OR “X-linked Intellectual Disability, Hedera Type”[tiab] OR “X-linked Intellectual Disability, Miles-carpenter Type”[tiab] OR “X-linked Intellectual Disability, Najm Type”[tiab] OR “X-linked Intellectual Disability, Nascimento Type”[tiab] OR “X-linked Intellectual Disability, Pai Type”[tiab] OR “X-linked Intellectual Disability, Porteous Type”[tiab] OR “X-linked Intellectual Disability, Schimke Type”[tiab] OR “X-linked Intellectual Disability, Seemanova Type”[tiab] OR “X-linked Intellectual Disability, Shashi Type”[tiab] OR “X-linked Intellectual Disability, Shrimpton Type”[tiab] OR “X-linked Intellectual Disability, Siderius Type”[tiab] OR “X-linked Intellectual Disability, Snyder Type”[tiab] OR “X-linked Intellectual Disability, Stevenson Type”[tiab] OR “X-linked Intellectual Disability, Stocco Dos Santos Type”[tiab] OR “X-linked Intellectual Disability, Stoll Type”[tiab] OR “X-linked Intellectual Disability, Sutherland-haan Type”[tiab] OR “X-linked Intellectual Disability, Van Esch Type”[tiab] OR “X-linked Intellectual Disability, Wilson Type”[tiab] OR “X-linked Intellectual Disability-acromegaly-hyperactivity Syndrome”[tiab] OR “X-linked Intellectual Disability-ataxia-apraxia Syndrome”[tiab] OR “X-linked Intellectual Disability-cardiomegaly-congestive Heart Failure Syndrome”[tiab] OR “X-linked Intellectual Disability-cerebellar Hypoplasia Syndrome”[tiab] OR “X-linked Intellectual Disability-cerebellar Hypoplasia-spondylo-epiphyseal Dysplasia Syndrome”[tiab] OR “X-linked Intellectual Disability-craniofacioskeletal Syndrome”[tiab] OR “X-linked Intellectual Disability-cubitus Valgus-dysmorphism Syndrome”[tiab] OR “X-linked Intellectual Disability-dysmorphism-cerebral Atrophy Syndrome”[tiab] OR “X-linked Intellectual Disability-epilepsy-progressive Joint Contractures-dysmorphism Syndrome”[tiab] OR “X-linked Intellectual Disability-global Development Delay-facial Dysmorphism-sacral Caudal Remnant Syndrome”[tiab] OR “X-linked Intellectual Disability-hypogammaglobulinemia-progressive Neurological Deterioration Syndrome”[tiab] OR “X-linked Intellectual Disability-hypogonadism-ichthyosis-obesity-short Stature Syndrome”[tiab] OR “X-linked Intellectual Disability-hypotonia-facial Dysmorphism-aggressive Behavior Syndrome”[tiab] OR “X-linked Intellectual Disability-hypotonia-movement Disorder Syndrome”[tiab] OR “X-linked Intellectual Disability-limb Spasticity-retinal Dystrophy-diabetes Insipidus Syndrome”[tiab] OR “X-linked Intellectual Disability-macrocephaly-macroorchidism Syndrome”[tiab] OR “X-linked Intellectual Disability-plagiocephaly Syndrome”[tiab] OR “X-linked Intellectual Disability-psychosis-macroorchidism Syndrome”[tiab] OR “X-linked Intellectual Disability-retinitis Pigmentosa Syndrome”[tiab] OR “X-linked Intellectual Disability-seizures-psoriasis Syndrome”[tiab] OR “X-linked Intellectual Disability-short Stature-overweight Syndrome”[tiab] OR “X-linked Lissencephaly With Abnormal Genitalia”[tiab] OR “X-linked Neurodegenerative Syndrome, Bertini Type”[tiab] OR “X-linked Neurodegenerative Syndrome, Hamel Type”[tiab] OR “X-linked Non-syndromic Intellectual Disability”[tiab] OR “X-linked Spastic Paraplegia Type 16”[tiab] OR “X-linked Spasticity-intellectual Disability-epilepsy Syndrome”[tiab] OR “Xeroderma Pigmentosum”[tiab] OR “Xeroderma Pigmentosum, Complementation Group A”[tiab] OR “Xeroderma Pigmentosum, Complementation Group B”[tiab] OR “Xeroderma Pigmentosum, Complementation Group D”[tiab] OR “Xeroderma Pigmentosum, Complementation Group F”[tiab] OR “Xeroderma Pigmentosum-cockayne Syndrome Complex”[tiab] OR “Xfe Progeroid Syndrome”[tiab] OR “Xia-gibbs Syndrome”[tiab] OR “Xp11.22 Microduplication Syndrome”[tiab] OR “Xp21 Deletion Syndrome”[tiab] OR “Xp22.13p22.2 Duplication Syndrome”[tiab] OR “Xq12-q13.3 Duplication Syndrome”[tiab] OR “Xq21 Deletion Syndrome”[tiab] OR “Xq21 Microdeletion Syndrome”[tiab] OR “Xq25 Duplication Syndrome”[tiab] OR “Xq25 Microduplication Syndrome”[tiab] OR “Xq27.3q28 Duplication Syndrome”[tiab] OR “Xq28 (mecp2) Duplication”[tiab] OR “Xy Type Gonadal Dysgenesis-associated Anomalies Syndrome”[tiab] OR “Xylt1-cdg”[tiab] OR “Yuan-harel-lupski Syndrome”[tiab] OR “Zaki Syndrome”[tiab] OR “Zechi-ceide Syndrome”[tiab] OR “Zellweger-like Syndrome Without Peroxisomal Anomalies”[tiab] OR “Zimmermann-laband Syndrome”[tiab] OR “Zimmermann-laband Syndrome 1”[tiab] OR “Zimmermann-laband Syndrome 2”[tiab] OR “Zimmermann-laband Syndrome 3”[tiab] OR “Zttk Syndrome”[tiab] OR “Zunich Neuroectodermal Syndrome”[tiab] |
