# Supplementary material for: Cancer risks related to intellectual disabilities: A systematic review
Source: Cancer Med. 2024 Apr 30;13(9):e7210. doi: 10.1002/cam4.7210 (PMC11058689; doi:10.1002/cam4.7210)
Supplement: Supplementary file 3 — Appendix S3: xxx. [file CAM4-13-e7210-s001.docx]

| Study | Sample size | Study population / Target population | Identification of intellectual disabilities | Cancer diagnosis | Statistical analysis | Conclusion |
| --- | --- | --- | --- | --- | --- | --- |
| Liu,Q ’21 | + | +/- | +/- | + | +/- | 7 |
| Patja,K ´01 | + | + | + | + | + | 10 |
| Satgé,D ‘20 | + | + | + | + | + | 10 |
| Sullivan ‘04 | + | + | + | + | + | 10 |
| Cuypers ‘20 | + | + | + | +/- | + | 9 |
| Trétarre, B '17 | + | +/- | +/- | - | + | 7 |
| Sullivan’03 | + | + | + | + | + | 10 |
|  |  |  |  |  |  |  |
| Bjorge,T | + | +/- | + | + | + | 9 |
| Boker, L ‘01 | + | +/- | + | + | + | 9 |
| Choi,Y ‘22 | + | + | + | + | + | 10 |
| Goldrace,A ‘04 | + | + | + | + | + | 10 |
| Hasle, H ‘16 | + | + | + | + | + | 10 |
| Hasle,H ‘00 | + | + | + | + | + | 10 |
| Hill,D ‘03 | + | + | + | + | + | 10 |
| Marlow,E ‘21 | + | + | + | + | + | 10 |
| Patje, K ‘06 | + | + | + | + | + | 10 |
| Sullivan,S ‘07 | + | + | + | + | + | 10 |
| Agha,M ‘04 | + | + | +/- | + | + | 9 |
| Kutler,D ‘03 | + | + | + | + | + | 10 |
| Rosenberg,P ‘03 | + | + | + | - | + | 9 |
| Rosenberg,P ‘08 | + | +/- | +/- | +/- | + | 7 |
| Dutzmann,C ‘22 | + | +/- | + | + | + | 9 |
| Sugranes,T ‘22 | + | + | + | + | + | 10 |
| Madanikia,S ‘12 | + | + | + | + | + | 10 |
| Marjanska,A 20 | + | + | + | +/- | - | 8 |
| Landry, P ‘21 | + | + | + | + | + | 10 |
| Seminong,O ‘15 | + | + | + | + | + | 10 |
| Uusitalo,E ‘17 | + | + | + | - | +/- | 8 |
| Walker, L ‘06 | + | + | + | + | + | 10 |
| Wang,X ‘12 | + | +/- | + | + | + | 9 |
| Uusitalo, E ’16 | + | + | + | - | + | 9 |
| Schultz-Pederson. A. ’01 | + | +/- | - | - | + | 7 |
| Sund, R ’09 | + | + | +/- | +/- | + | 8 |
| Boot,M ‘18 | + | +/- | + | + | - | 8 |
| Davies,H ‘03 | + | +/- | +/- | - | + | 6 |
| Greene,A ‘04 | + | +/- | +/- | +/- | +/- | 6 |
| Sahlin,J ‘07 | + | +/- | + | +/- | +/- | 7 |
| Heald,B ‘10 | + | +/- | +/- | + | + | 8 |
| Kratz,C ‘15 | + | +/- | + | + | +/- | 8 |
| Peron,A ‘16 | + | +/- | + | + | - | 8 |
|  |  |  |  |  |  |  |
| Jiang, J‘16 | + | + | +/- | + | + | 9 |
| Roznoski,K ‘08 | + | +/- | + | + | + | 9 |
| Bogdanova,N ‘08 | + | + | + | +/- | + | 9 |
| Lawania,S ‘17 | + | + | + | + | + | 10 |
| Paszkowska-Szczur, K ‘12 | + | + | + | + | + | 10 |
| Shao,J ‘07 | + | + | + | + | + | 10 |
| Xie,Y ‘21 | + | + | + | + | + | 10 |
| Yang, Z ‘08’ | + | + | + | + | + | 10 |
| Yuan,L ‘11 | + | + | + | + | + | 10 |
| Samson,M ‘11 | + | + | + | + | + | 10 |
| Jorgensen,T ’06 | + | + | + | + | +/- | 9 |
| Chang, C ‘09 | + | + | + | + | + | 10 |
| Cui,Y ‘06 | + | + | + | + | + | 10 |
| Jeon, H ‘03 | + | + | + | + | + | 10 |
| Miller,K ‘06 | + | +/- | + | + | + | 9 |

**Legenda**

For each specific study type a critical appraisal checklist was used from the JBI.

Scoring is based on the three-point scale, a + is equal to 2 points, +/- is given a score of 1 point and – is scored 0 points. The quality assessment can be assessed max 10 points.

Sufficient: 10-8
Moderate: 7-6
Insufficient: < 5

Explanation of the following topics:

**Sample size**: Assessed according to the total of study-participants within the research, at least more than 50 included.

**Study population (target population):** Assessment is based on the presence of a control group (general population), and a description regarding the demographics, clinical information etc. of the study participants.

**Identification of intellectual disability:** grading for this topic was determined by the presence of a detailed description regarding the methods used for detecting ID (or genetic mutations), e.g. data-systems, coding-systems etc.

**Cancer diagnosis:** Was scored according to clarity of cancer diagnosis in the study population.

**Statistical analysis:** If statistical analysis was described and used appropriately.

For all topics, if the retrieval or the description of the specific topic was unclear it was scored a +/-. If no description was given, it was immediately scored as insufficient (-).
